# Sulfoximines-Assisted Rh(III)-Catalyzed C–H Activation and Intramolecular Annulation for the Synthesis of Fused Isochromeno-1,2-Benzothiazines Scaffolds under Room Temperature

**DOI:** 10.3390/molecules25112515

**Published:** 2020-05-28

**Authors:** Bao Wang, Xu Han, Jian Li, Chunpu Li, Hong Liu

**Affiliations:** 1 State Key Laboratory of Drug Research and CAS Key Laboratory of Receptor Research, Shanghai Institute of Materia Medica, Chinese Academy of Sciences, 555 Zu Chong Zhi Road, Shanghai 201203, China; wangbao@shanghaitech.edu.cn (B.W.); 201718012342004@simm.ac.cn (X.H.); jianl@simm.ac.cn (J.L.); 2 University of Chinese Academy of Sciences, No.19A Yuquan Road, Beijing 100049, China; 3 School of Life Science and Technology, ShanghaiTech University, 100 Haike Road, Shanghai 201210, China

**Keywords:** sulfoximide, C–H activation, benzothiazine, rhodium

## Abstract

A mild and facile Cp*Rh(III)-catalyzed C–H activation and intramolecular cascade annulation protocol has been proposed for the furnishing of highly fused isochromeno-1,2-benzothiazines scaffolds using *S*-phenylsulfoximides and 4-diazoisochroman-3-imine as substrates under room temperature. This method features diverse substituents and functional groups tolerance and relatively mild reaction conditions with moderate to excellent yields. Additionally, retentive configuration of sulfoximides in the conversion has been verified.

## 1. Introduction

**Figure 1:**
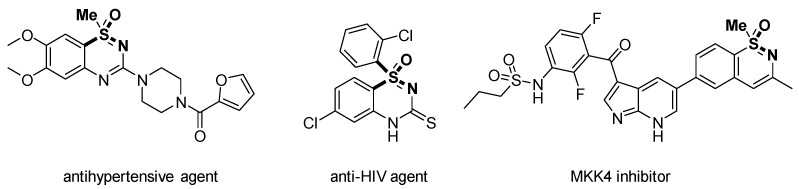
Representatives of biologically active compounds containing sulfoximines moiety.

Over the past decade, sulfoximines moiety has gained an increasing attention in organic chemistry [1,2,3,4,5,6,7,8,9] and pharmaceutical industries for their interesting properties such as multiple hydrogen-bond acceptor/donor functionalities, structural diversity, and favorable physicochemical properties [10,11,12,13]. For instances, sulfoximines along with benzothiazines scaffold-containing compounds possess diversified biologically active molecules such as antihypertensive activity *α*-adrenergic receptor blocker [14], anti-HIV nonnucleoside reverse transcriptase inhibitors [15], and hepatocytes protective mitogen-activated protein kinase kinase 4 (MKK4) inhibitor (Figure 1).

**Scheme 1:**
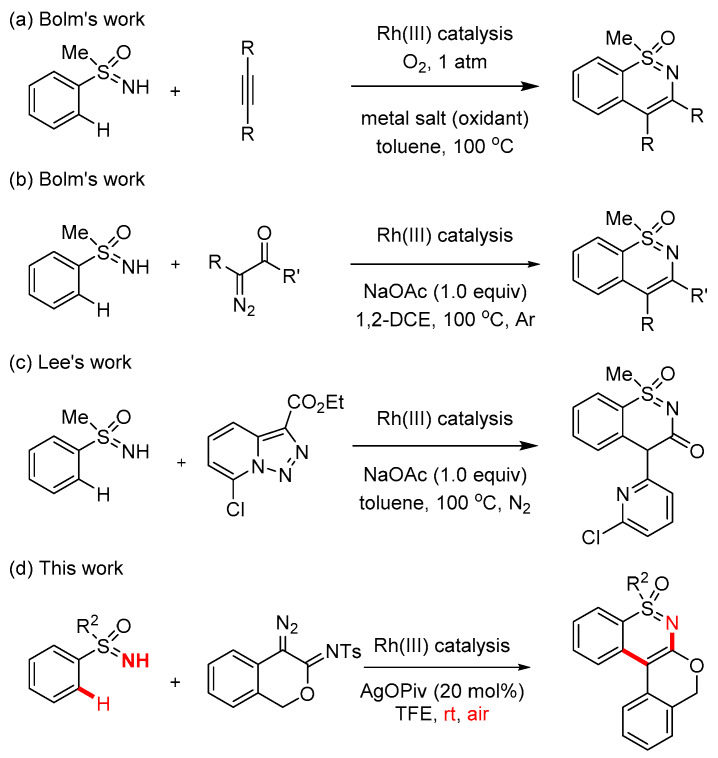
Synthetic methods access to sulfoximines. (**a**) Bolm’s work; (**b**) Bolm’s work; (**c**) Lee’s work; (**d**) This work.

Considering the significance of the sulfoximines motif as a pharmacophore in medicinal chemistry, synthetic methods accessing to this moiety have been increasingly studied. The typical route approach to the sulfoximines starting from commercially available sulfides requires two steps include oxidation and successively imination [6,16,17]. Sometimes, in order to produce the *N*-free sulfoximines, an additional step should be involved to dissociate the protecting group. Thus, these traditional methods have drawbacks such as requiring relatively harsh reaction conditions and poor step-economy for constructing cyclic sulfoximines. In recent years, transition-metal-catalyzed direct C–H functionalization has been broadly investigated, and these strategies have been proven to be an efficient tool for the rapid construction of C–C, C–O, C–N, or C–S bonds [18,19,20,21,22,23,24,25,26,27,28,29,30]. Additionally, owing to the high efficiency and easy accessibility of Rh(III) catalysis, the construction of 1,2-benzothiazines through Rh(III)-catalyzed C–H bond activation has attracted attention and been extensively studied [31,32,33]. For instance, in 2013, Bolm and coworkers developed a Rh(III)-catalyzed C–H functionalization for the synthesis of 1,2-benzothiazines starting from *N*-*H*-sulfoximines and alkynes (Scheme 1a) [34]. This process had been accomplished under 1 atm O_2_ at 100 °C, and the desired products could be yielded in good to excellent yields but with limited structural diversity. Later in 2015, Bolm et al. has disclosed another strategy approaching the 1,2-benzothiazines via sulfoximines-assisted Rh(III)-catalyzed C–H functionalization and coupling reaction with diazo compounds (Scheme 1b) [35]. It is noteworthy that the dizao coupling partners should be electro-withdrawing groups-incorporated moieties, which lead to a limited versatility, and the process was carried out under argon at 100 °C. Recently, in Lee’s group, an Rh(III)-catalyzed domino C–H activation/cyclization strategy has been reported by mixing sulfoximine and pyridotriazole compounds to build the 1,2-benzothiazines skeleton (Scheme 1c) [36]. In the methodology, the target products were 1,2-benzothiazines bearing pyridyl motifs as well as carbonyl groups and the process was conducted at a high reaction temperature. However, it is of note that most of these strategies have to be conducted at harsh reaction conditions with a limited reaction versatility and structural diversity. Only in 2019, Wu et al. reported a mild protocol to synthesize 1,2-benzothiazines derivatives [37]. Moreover, the constructed 1,2-benzothiazines scaffolds were bicyclic moieties, and to the best of our knowledge, only limited examples have been disclosed for the synthesis of fused 1,2-benzothiazines motif under harsh reaction conditions [38].

In continuation of our studies on the establishment of fused ring heterocyclic compounds via Rh(III)-catalyzed C-H functionalization [39,40,41], we envisioned that a fused 1,2-benzothiazines scaffold could be achieved via an Rh(III)-catalyzed C–H functionalization/annulation between sulfoximines and 4-diazoisochroman-3-imines. To our delight, the fused isochromeno-1,2-benzothiazines were successfully accomplished in good to excellent yields through a redox-neutral process, and our strategy could be carried out in the air under room temperature with broad generality and versatility. We herein described our results in detail.

## 2. Results and Discussion

Based on our previous work and relevant reports, we initially focused the studies on the Rh(III)-catalyzed coupling of sulfoximide (**1a**) and 4-diazoisochroman-3-imine (**2a**) (Table 1). In the presence of 10 mol% [Cp*RhCl_2_]_2_ and 40 mol% AgSbF_6_ in dichloroethane (DCE) at 80 °C, the desired product **3aa** was produced in 16% yield, and its structure was further confirmed by ^1^H NMR spectroscopy (Table 1, Entry 2). We further assessed the reaction temperature in different solvents (Table 1, Entries 1–5), and the results demonstrated that when applying hexafluoro-isopropanol (HFIP) as the solvent, the reaction could be preceded by producing the **3aa** in 47% yield under room temperature. This result leads us to realize that the polar alcohols would more facilitate the reaction at mild conditions rather than alkane such as DCE, which might retarded the process (Table 1, Entries 4 and 5). Inspired by this result, the other reaction solvents were screened under room temperature (Table 1, Entries 6–9). After a series of solvents was examined, the trifluoroethanol (TFE) was found to be the optimal in which **3aa** was obtained in 75% yield (Table 1, Entry 6), and the structure of the desired product was further verified by X-ray crystallography (CCDC 1988905). We next investigated the effect of additives and gratifyingly, AgOPiv emerged to be the most effective additive among all those examined ones (Table 1, entries 10–13) to afford the desired product in an excellent isolated yield of 92%. Afterward, the ratio of the catalyst and additive was subsequently conducted (Table 1, Entries 14–16). The results disclosed that the 1:4 ratio of catalyst and additive was necessary for the full conversion of the starting material **1a** and **2a** (Table 1, Entries 13–17). In addition, further exploration proved that there was no obvious discrepancy of the yield of **3aa** when the reaction was moved to an argon atmosphere (Table 1, Entry 18). However, when the reaction time was shortened to 12 h, the yield of **3aa** was slightly reduced (Table 1, Entry 19). Moreover, we also tested different transition-metal catalysts such as the cost-effective ruthenium (II) or iridium (III) complex, and **3aa** could be only detected in 85% yield when treated with 10 mol% [Cp*lrCl_2_]_2_ as catalyst (Table 1, entries 20–21). Notably, **3aa** could not be detected when in the absence of [Cp*RhCl_2_]_2_, which indicated the [Cp*RhCl_2_]_2_ catalyst is indispensable for this transformation (Table 1, Entry 22). Therefore, the standard conditions for this Rh(III)-catalyzed coupling reaction is 5 mol %[Cp*RhCl_2_]_2_ and 20 mol %AgOPiv in TFE at room temperature for 18 h in the air.

After establishing the optimal reaction conditions, we next turned to investigate the scope of sulfoximide derivatives. As illustrated in Table 2, this Rh-catalyzed coupling reaction could proceed smoothly with substituted *S*-aryl sulfoximine substrates bearing electron-donating or electron-withdrawing substituents, and the corresponding products could be yielded in moderate to good yields. Sulfoximines with substituents including methyl, methoxyl, halogen, nitro, etc. installed at the *para*-position of the benzene ring coupled with **2a** smoothly to afford the isochromeno-benzothiazines products in good yields (**3ca**–**3ha**), albeit methyl substituted product (**3ba**) was obtained in only 47% yield. It should be mentioned that functional groups such as carbonyl, ester, or carbamate were all viable under the standard reaction conditions to give the desired products in 66% (**3ia**), 82% (**3ja**), and 89% (**3ka**) yields, respectively. This result indicated that the strategy could be broadly extended for further transformation. The *ortho*-methyl-incorporated sulfoximines also gave a relatively low yield of the desired product (**3la**, 58% yield). Notably, the substituents of sulfoximine substrates at different positions of its benzene ring did not alter the reaction efficiency, as for the halogenated substrates (compare **3ga**, **3ma**, and **3na**) provided the desired products in similar yields (72% to 74%). Interestingly, when an electron-withdrawing group such as bromo was installed at the *meta*-position of the benene ring in **1a**, only the *o*-C-H bond of sulfoximine located on the less hindered site was activated, which led to a production of **3na** as a single isomer in 72% yield. On the contrary, if an electron-donating group was incorporated on the meta-position, the products were isolated as a regioisomer (**3oa** and **3oa’**) in a total yield of 50%. In addition, naphthalene-fused sulfoximide was also coupled with **2a** smoothly, and the desired product **3pa** was obtained as a single isomer through activation/annulation on the less hindered site of sulfoximine in 73% yield. 

We also evaluated a variety of *S*-substituted phenylsulfoximine substrates (**3qa**–**3va**), and the results revealed that alkyl, halogen, hydroxyl, and aryl substituents on sulfur substrates were also compatible under the standard conditions and generated the corresponding product in moderate to good yields varying from 40% to 90%. In particularly, the cyclopropyl with huge steric hindrance effect exhibited no impact on the reaction efficiency and showed the best reactivity with 90% yield. Additionally, pyridine sulfoximine substrate could not generate the desired product (**3wa**), which might be caused by the strong coordination effect of the nitrogen atom in pyridine, which ceased the reaction process.

Subsequently, we investigated the scope of 4-diazoisochroman-3-imine coupling partner (Table 3). The introduction of both electron-donating or electron-withdrawing substituents including methyl, methoxy, Cl, F, and trifluoromethyl at the 6- or 7- positions of isochroman were all tolerated in this coupling reaction (**3ab**–**3aj**) and the yields were varying from 53% to 76%. Among all the substituents, the electron-deficient trifluoromethyl isochroman substrates exhibited good reactivity and independently furnished the products **3aj** and **3ai** in 76% and 74% yields. Besides, the nitro group-substituted substrate was not compatible for this coupling reaction, and no desired product (**3ak**) was achieved. Substituents at different positions has no obvious influence on the reaction efficiency, as the yields of the 6- and 7- substituted substrates are basically the same.

**Scheme 2:**
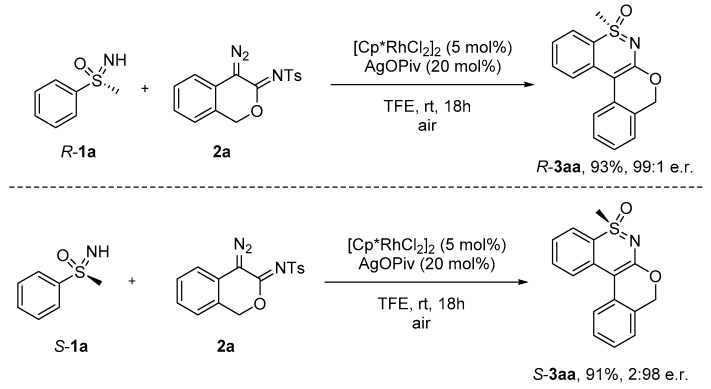
Conversion of stereoisomer substrates.

The chirality of the sufoximine group is important [42,43,44,45,46]. In order to verify the stereospecificity in the whole reaction process, optically pure *R*- and *S*-configured **1a** were parallelly coupled with **2a** under the standard condition (Scheme 2). The results demonstrated that the corresponding products were obtained with a retention of configuration with no erosion of the enantiopurity of the sulfoximine occurred, which indicated that the current coupling protocol possesses a potential utility on asymmetric synthesis.

**Scheme 3:**
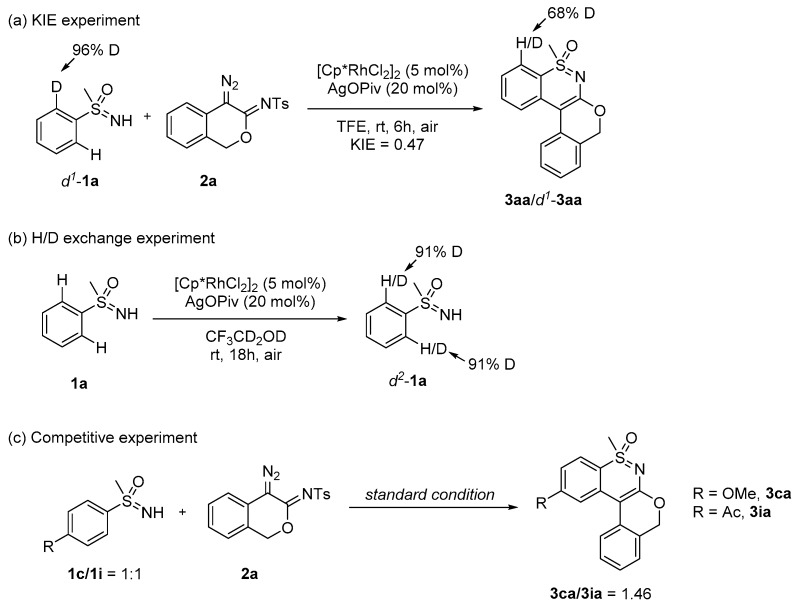
Preliminary mechanistic experiments. (**a**) KIE experiment; (**b**) H/D exchange experiment; (**c**) Competitive experiment.

Next, we conducted a series of control experiments to explore the preliminary reaction mechanism (Scheme 3). First, the kinetic isotope effect (KIE) experiment in intramolecular between *d*^1^-**1a** and **2a** were performed and a small KIE value (*k_H_/k_D_*) was measured as 0.47, which indicated that the aryl Csp^2^-H bond cleavage was not the rate-limiting step (Scheme 3a) [47]. Next, in the H/D exchange study, phenylsulfoximine **1a** was conducted under the standard conditions in deuterium TFE in the absence of **2a**, and the deuterated *d*^2^-**1a** recovered in 91% deuterium at the *ortho*-position of benzene, which suggested that the C–H bond activation process was reversible (Scheme 3b). Finally, an intermolecular competitive experiment between electron-rich and electron-deficient substrates (**1c**/**1i** = 1:1) lead to the products **3ca**/**3ia** with a ratio of 1.46, implying that an electrophilic rhodation of C–H bond activation probably involved in the catalytic cycle (Scheme 3c).

**Scheme 4:**
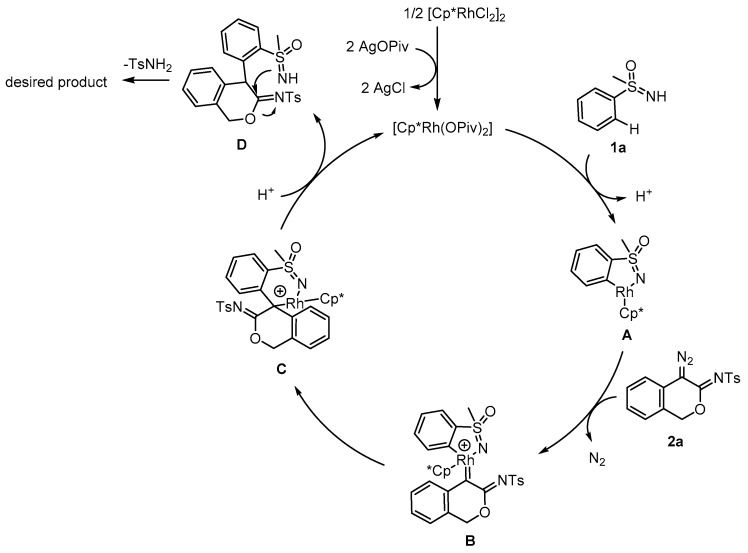
Proposed mechanism.

A plausible mechanism has been proposed based on the preliminary mechanistic experiments and previous reports [33,38,41,48]. As shown in Scheme 4, initially the five-membered rhodacycle intermediate **A** was formed via coordination of the activated rhodium catalyst with the nitrogen of the sulfoximine moiety and undergoes electrophilic C–H bond cleavage at the benzene *ortho*-position of substrate **1a**. Then, intermediate **A** coordinated with **2a** generates rhodium carbenoid intermediate **B**, followed by intramolecular carbene migratory insertion and produced a six-membered rhodacycle intermediate **C**. Finally, the intermediate **C** protonation to yield compound **D** and regenerated the active cationic Cp*Rh(III) species for the next catalytic cycle. Compound **D** could easily undergo intramolecular nucleophilic attack of imine and elimination of the *p*-toluenesulfonamide (TsNH_2_) process to produce the desired isochromeno-1,2-benzothiazines product **3aa**.

## 3. Materials and Methods 

### 3.1. General Information 

All reagents and solvents were purchased from commercial sources (J&K Scientific Co., Ltd., Beijing, China; TCI Development Co., Ltd., Shanghai, China; Adamas Reagent, Co., Ltd., Shanghai, China.) and used without further purification. The analytical thin layer chromatography (TLC) was HSGF 254 (0.15–0.2 mm thickness). All products were characterized by their NMR and MS spectra. ^1^H and ^13^C nuclear magnetic resonance spectra (NMR) were acquired on a Bruker 400 MHz or 500 MHz or 600 MHz NMR spectrometer (Billerica, MA, USA). Chemical shifts were reported in parts per million (ppm, δ) downfield from tetramethylsilane, and the coupling constants (*J*) were indicated in Hz. Proton coupling patterns are described as singlet (s), doublet (d), triplet (t), quartet (q), multiplet (m), doublet of doublets (dd), and broad (br). Low-resolution mass spectra (LRMS) data were measured on Agilent 1260 Infinity II (Palo Alto, CA, USA) with Electrospray Ionization (ESI). High-resolution mass spectra (HRMS) data were measured on an Agilent G6520 Q-TOF (Palo Alto, CA, USA) with Electrospray Ionization (ESI). AgOPiv was prepared according to the reported literature method [49].

### 3.2. Experimental Part Method

#### 3.2.1. General Procedure A for the Synthesis of Substrates **1a**–**1w**

To a stirred solution of sulfide (1 mmol) in MeOH (10 mL) was added (NH_4_)_2_CO_3_ (1.5 equiv.). Subsequently, PhI(OAc)_2_ (2.3 equiv.) was added, and the solution was stirred at rt for 10 min. The solvent was removed under reduced pressure, and the crude product was purified by flash column chromatography eluted with dichloromethane (DCM)/MeOH from 30:1 to 10:1 to give the desired product **1**. Compounds **1e**, **1f**, **1q**, *R*-**1a,** and *S*-**1a** were purchased from commercial sources and used without further purification. Compounds **1a**–**1c**, **1g**-**1i**, **1l**–**1p**, **1r**–**1w** are known compounds.

[4-(*S*-Methylsulfonimidoyl)phenyl]methanol (**1d**): white solid; m.p.: 108–109 °C; ^1^H NMR (500 MHz, DMSO-*d*_6_): *δ* 7.88 (d, *J* = 8.3 Hz, 2H), 7.55–7.49 (m, 2H), 5.41 (t, *J* = 5.7 Hz, 1H), 4.59 (d, *J* = 5.6 Hz, 2H), 4.15 (s, 1H), 3.04 (s, 3H); ^13^C NMR (126 MHz, DMSO-*d*_6_): *δ* 147.5, 142.3, 127.2, 126.6, 62.2, 46.0; LRMS (ESI): *m*/*z* 186.0 [M + H]^+^; HRMS (ESI): calculated for C_8_H_12_NO_2_S [M + H]^+^: 186.0583, found: 186.0584.

Ethyl [4-(*S*-methylsulfonimidoyl)phenyl]acetate (**1j**): colorless oil (207.5 mg, 86% yield); ^1^H NMR (600 MHz, DMSO-*d*_6_): *δ* 7.88 (d, *J* = 8.3 Hz, 2H), 7.49 (d, *J* = 8.4 Hz, 2H), 4.18 (s, 1H), 4.09 (q, *J* = 7.1 Hz, 2H), 3.80 (s, 2H), 3.05 (s, 3H), 1.19 (t, *J* = 7.1 Hz, 3H); ^13^C NMR (126 MHz, CDCl_3_): δ 170.6, 142.5, 139.8, 130.4, 128.1, 61.4, 46.3, 41.2, 14.3; LRMS (ESI): *m*/*z* 242.1 [M + H]^+^; HRMS (ESI): calculated for C_11_H_16_NO_3_S [M + H]^+^: 242.0845, found: 242.0841.

*tert*-Butyl (4-(*S*-methylsulfonimidoyl)phenyl)carbamate (**1k**): white solid; m.p.: 155–156 °C; ^1^H NMR (600 MHz, DMSO-*d*_6_): *δ* 9.78 (s, 1H), 7.80 (d, *J* = 8.7 Hz, 2H), 7.63 (d, *J* = 8.5 Hz, 2H), 4.01 (s, 1H), 3.00 (s, 3H), 1.49 (s, 9H); ^13^C NMR (126 MHz, DMSO-*d*_6_): *δ* 152.5, 143.3, 136.7, 128.4, 117.5, 79.8, 46.2, 28.0; LRMS (ESI) *m*/*z*: 271.1 [M + H]^+^; HRMS (ESI) *m*/*z*: calculated for C_12_H_18_N_2_O_3_S [M + H]^+^: 271.1111, found: 271.1115.

#### 3.2.2. General Procedure for the Synthesis of Substrates **2a**–**2k**

To a two-neck round bottom flask was successively added (2-ethynylphenyl)methanols (5.0 mmol, 1.0 equiv.), CuBr (0.5 mmol, 0.1 equiv.), and Et_3_N (10.0 mmol, 2.0 equiv.) in anhydrous MeCN (50 mL, 0.1 M). The mixture was evacuated and refilled with Ar 3 times. To the resulting mixture was slowly added *p*-toluenesulfonyl azide (75% in EA solution, 11.0 mmol, 2.2 equiv.) over 10 min under Ar, and the reaction was processed under room temperature for 4–6 h. Then, the mixture was filtered through a pad of celite and washed with DCM (30 mL × 3). The combined organic layer was washed with saturated NaHCO_3_, dried over anhydrous Na_2_SO_4_, filtered, and concentrated under reduced pressure. The crude residue was purified by flash chromatography eluted with hexane/EA/DCM = 5:1:2 to give the desired product **2** as a yellow solid. Compounds **2a**–**2k** are known compounds. 

#### 3.2.3. General Procedure for the Synthesis of Compounds **3**

To a 15 mL vial was added sulfoximines **1** (0.15 mmol), 4-diazoisochroman-3-imines **2** (0.165 mmol), [Cp*RhCl_2_]_2_ (5 mol%), and AgOPiv (20 mol%) under air. Trifluoroethanol (TFE, 2.5 mL) was added subsequently. The resulting mixture was stirred at ambient temperature for 18 h. Upon completion of the reaction, the mixture was filtered through a celite pad and washed with DCM (10 mL × 3). The combined organic layer was concentrated under vacuo, and the residue was purified by silica gel chromatography eluting with DCM/MeOH from 50:1 to 10:1 to give the desired isochromeno-1,2-benzothiazines product **3**.

#### 3.2.4. Characterization of the Products

5-Methyl-8H-5λ^4^-isochromeno[3,4-*c*][1,2]benzothiazine 5-oxide (**3aa**): yellow-green solid; m.p.: 182–184 °C; ^1^H NMR (500 MHz, CDCl_3_): *δ* 8.07 (d, *J* = 9.6 Hz, 1H), 7.78 (dd, *J* = 8.0, 1.3 Hz, 1H), 7.62–7.54 (m, 2H), 7.38–7.26 (m, 2H), 7.21–7.11 (m, 2H), 5.20–4.99 (m, 2H), 3.49 (s, 3H); ^13^C NMR (126 MHz, CDCl_3_): *δ* 157.3, 135.0, 132.9, 131.3, 128.8, 128.2, 125.0, 124.8, 124.5, 124.4, 123.2, 122.2, 120.0, 91.9, 70.3, 43.0; LRMS (ESI): *m*/*z* 284.1 [M + H]^+^; HRMS (ESI): calculated for C_16_H_14_NO_2_S [M + H]^+^: 284.0740, found: 284.0745.

2,5-Dimethyl-8H-5λ^4^-isochromeno[3,4-*c*][1,2]benzothiazine 5-oxide (**3ba**): yellow solid; m.p.: 101–103 °C; ^1^H NMR (600 MHz, CDCl_3_): *δ* 7.87 (s, 1H), 7.67 (d, *J* = 8.2 Hz, 1H), 7.61 (d, *J* = 8.8 Hz, 1H), 7.32 (t, *J* = 6.8 Hz, 1H), 7.20 (d, *J* = 7.4 Hz, 1H), 7.18–7.13 (m, 2H), 5.14–5.00 (m, 2H), 3.46 (s, 3H), 2.44 (s, 3H); ^13^C NMR (151 MHz, CDCl_3_): *δ* 157.3, 143.7, 135.2, 131.4, 128.8, 128.2, 125.7, 125.0, 124.7, 124.2, 123.2, 122.2, 117.6, 91.6, 70.2, 43.1, 22.2; LRMS (ESI): *m*/*z* 298.0 [M + H]^+^; HRMS (ESI): calculated for C_17_H_16_NO_2_S [M + H]^+^: 298.0896, found: 298.0900.

2-Methoxy-5-methyl-8H-5λ^4^-isochromeno[3,4-*c*][1,2]benzothiazine 5-oxide (**3ca**): pale yellow solid; m.p.: 105–106 °C; ^1^H NMR (400 MHz, CDCl_3_): *δ* 7.70 (d, *J* = 8.9 Hz, 1H), 7.64 (d, *J* = 7.8 Hz, 1H), 7.47 (d, *J* = 2.4 Hz, 1H), 7.35–7.27 (m, 1H), 7.20 (d, *J* = 5.9 Hz, 1H), 7.14 (t, *J* = 7.3 Hz, 1H), 6.88 (dd, *J* = 8.9, 2.4 Hz, 1H), 5.07 (q, *J* = 12.2 Hz, 2H), 3.86 (s, 3H), 3.42 (s, 3H); ^13^C NMR (126 MHz, CDCl_3_): *δ* 163.3, 157.9, 137.5, 131.5, 128.9, 128.2, 125.5, 125.1, 124.6, 121.8, 112.9, 112.8, 106.7, 91.5, 70.1, 55.7, 43.7; LRMS (ESI): *m*/*z* 314.0 [M + H]^+^; HRMS (ESI): calculated for C_17_H_16_NO_3_S [M + H]^+^: 314.0845, found: 314.0844.

(5-Methyl-5-oxido-8H-5λ^4^-isochromeno[3,4-*c*][1,2]benzothiazin-2-yl)methanol (**3da**): orange solid; m.p.: 65–67 °C; ^1^H NMR (600 MHz, CDCl_3_): *δ* 7.99 (s, 1H), 7.66 (d, *J* = 8.2 Hz, 1H), 7.54 (d, *J* = 7.8 Hz, 1H), 7.29–7.23 (m, 2H), 7.13 (d, *J* = 6.8 Hz, 2H), 5.01–4.93 (m, 2H), 4.73 (s, 2H), 3.40 (s, 3H), 2.97 (s, 1H); ^13^C NMR (126 MHz, CDCl_3_): *δ* 157.2, 146.6, 135.0, 131.1, 128.7, 128.3, 125.0, 124.8, 123.4, 122.8, 122.1, 121.7, 118.6, 92.0, 70.1, 64.5, 42.9; LRMS (ESI): *m*/*z* 314.0 [M + H]^+^; HRMS (ESI): calculated for C_17_H_16_NO_3_S [M + H]^+^: 314.0845, found: 314.0846.

2-Fluoro-5-methyl-8H-5λ^4^-isochromeno[3,4-*c*][1,2]benzothiazine 5-oxide (**3ea**): yellow solid; m.p.: 149–151 °C; ^1^H NMR (600 MHz, CDCl_3_): *δ* 7.79 (dd, *J* = 8.9, 5.5 Hz, 1H), 7.70 (dd, *J* = 11.3, 2.5 Hz, 1H), 7.57 (d, *J* = 7.6 Hz, 1H), 7.33 (td, *J* = 7.5, 1.7 Hz, 1H), 7.22–7.13 (m, 2H), 7.07–6.99 (m, 1H), 5.15–5.02 (m, 2H), 3.47 (s, 3H); ^13^C NMR (151 MHz, CDCl_3_): *δ* 157.8, 134.5, 133.5, 132.2, 131.9, 131.1–130.2 (m), 125.5, 125.0, 124.0, 123.4, 121.5–121.3 (m), 120.2, 118.6 (q, *J* = 3.8 Hz), 91.3, 69.7, 43.0; LRMS (ESI): *m*/*z* 302.0 [M + H]^+^; HRMS (ESI): calculated for C_16_H_13_FNO_2_S [M + H]^+^: 302.0646, found: 302.0650.

2-Chloro-5-methyl-8H-5λ^4^-isochromeno[3,4-*c*][1,2]benzothiazine 5-oxide (**3fa**): yellow-green solid; m.p.: 210–211 °C; ^1^H NMR (600 MHz, CDCl_3_): *δ* 8.04 (d, *J* = 2.0 Hz, 1H), 7.70 (d, *J* = 8.6 Hz, 1H), 7.57 (d, *J* = 7.8 Hz, 1H), 7.39–7.30 (m, 1H), 7.28 (d, *J* = 1.9 Hz, 1H), 7.22–7.15 (m, 2H), 5.15–5.02 (m, 2H), 3.48 (s, 3H); ^13^C NMR (151 MHz, CDCl_3_): *δ* 157.6, 139.2, 136.0, 130.2, 128.2, 127.9, 124.6, 124.6, 124.3, 124.0, 123.2, 121.5, 117.3, 91.1, 69.8, 42.6; LRMS (ESI): *m*/*z* 318.0 [M + H]^+^; HRMS (ESI): calculated for C_16_H_13_ClNO_2_S [M + H]^+^: 318.0350, found: 318.0350.

2-Bromo-5-methyl-8H-5λ^4^-isochromeno[3,4-*c*][1,2]benzothiazine 5-oxide (**3ga**): yellow-green solid; m.p.: 93–94 °C; ^1^H NMR (500 MHz, CDCl_3_): *δ* 8.21 (d, *J* = 1.8 Hz, 1H), 7.62 (d, *J* = 8.5 Hz, 1H), 7.56 (d, *J* = 7.8 Hz, 1H), 7.42 (dd, *J* = 8.5, 1.8 Hz, 1H), 7.37–7.33 (m, 1H), 7.22 – 7.15 (m, 2H), 5.16–5.02 (m, 2H), 3.48 (s, 3H).; ^13^C NMR (126 MHz, CDCl_3_): *δ* 157.6, 136.1, 130.1, 128.2, 128.0, 127.8, 126.8, 126.3, 124.6, 124.6, 124.2, 121.5, 117.7, 91.0, 69.8, 42.5; LRMS (ESI): *m*/*z* 361.0 [M + H]^+^; HRMS (ESI): calculated for C_16_H_13_BrNO_2_S [M + H]^+^: 361.9845, found: 361.9850.

5-Methyl-2-nitro-8H-5λ^4^-isochromeno[3,4-*c*][1,2]benzothiazine 5-oxide (**3ha**): brown solid; m.p.: 179–181 °C; ^1^H NMR (600 MHz, CDCl_3_): *δ* 8.92 (d, *J* = 2.2 Hz, 1H), 8.04 (dd, *J* = 8.7, 2.1 Hz, 1H), 7.91 (d, *J* = 8.7 Hz, 1H), 7.56 (d, *J* = 7.8 Hz, 1H), 7.39 (dt, *J* = 8.2, 4.4 Hz, 1H), 7.23 (d, *J* = 4.1 Hz, 2H), 5.21 – 5.03 (m, 2H), 3.61 (s, 3H); ^13^C NMR (126 MHz, CDCl_3_): *δ* 158.1, 150.0, 135.5, 129.6, 128.3, 128.1, 125.3, 124.8, 124.3, 121.8, 121.4, 119.5, 117.3, 92.6, 70.0, 42.2; LRMS (ESI): *m*/*z* 329.2 [M + H]^+^; HRMS (ESI): calculated for C_16_H_13_N_2_O_4_S [M + H]^+^: 329.0591, found: 329.0603.

1-(5-Methyl-5-oxido-8H-5λ^4^-isochromeno[3,4-*c*][1,2]benzothiazin-2-yl)ethanone (**3ia**): orange-yellow solid; m.p.: 112–114 °C; ^1^H NMR (600 MHz, CDCl_3_): *δ* 8.63 (s, 1H), 7.86–7.80 (m, 2H), 7.57 (d, *J* = 7.8 Hz, 1H), 7.35 (td, *J* = 7.5, 1.7 Hz, 1H), 7.24–7.16 (m, 2H), 5.21–5.02 (m, 2H), 3.57 (s, 3H), 2.63 (s, 3H); ^13^C NMR (151 MHz, CDCl_3_): *δ* 197.4, 157.8, 140.1, 135.1, 130.8, 128.7, 128.6, 125.3, 125.2, 123.6, 123.0, 122.0, 122.0, 92.7, 70.4, 42.7, 27.1; LRMS (ESI): *m*/*z* 326.0 [M + H]^+^; HRMS (ESI): calculated for C_18_H_16_NO_3_S [M + H]^+^: 326.0845, found: 326.0850.

Ethyl (5-methyl-5-oxido-8H-5λ^4^-isochromeno[3,4-*c*][1,2]benzothiazin-2-yl)acetate (**3ja**): yellow solid; m.p.: 70–72 °C; ^1^H NMR (600 MHz, CDCl_3_): *δ* 7.96 (d, *J* = 1.8 Hz, 1H), 7.73 (d, *J* = 8.2 Hz, 1H), 7.59 (d, *J* = 7.9 Hz, 1H), 7.31 (td, *J* = 7.5, 1.6 Hz, 1H), 7.25 (d, *J* = 1.6 Hz, 1H), 7.19 (dd, *J* = 7.6, 1.5 Hz, 1H), 7.15 (td, *J* = 7.4, 1.1 Hz, 1H), 5.17–4.97 (m, 2H), 4.17 (q, *J* = 7.1 Hz, 2H), 3.68 (s, 2H), 3.48 (s, 3H), 1.27 (t, *J* = 7.1 Hz, 3H); ^13^C NMR (126 MHz, CDCl_3_): *δ* 170.7, 157.5, 139.4, 135.2, 131.2, 128.8, 128.2, 125.5, 125.0, 125.0, 124.8, 123.5, 122.1, 118.7, 91.8, 70.2, 61.3, 43.0, 41.7, 14.3; LRMS (ESI): *m*/*z* 370.0 [M + H]^+^; HRMS (ESI): calculated for C_20_H_20_NO_4_S [M + H]^+^: 370.1108, found: 370.1116.

*tert*-Butyl (5-methyl-5-oxido-8H-5λ^4^-isochromeno[3,4-*c*][1,2]benzothiazin-2-yl)carbamate (**3ka**): yellow-green solid; m.p.: 77–79 °C; ^1^H NMR (600 MHz, CDCl_3_): *δ* 7.90 (d, *J* = 2.0 Hz, 1H), 7.65 (d, *J* = 8.8 Hz, 1H), 7.59 (d, *J* = 7.4 Hz, 1H), 7.46 (d, *J* = 8.6 Hz, 1H), 7.30 – 7.24 (m, 1H), 7.13 (d, *J* = 7.5 Hz, 1H), 7.09 (t, *J* = 7.4 Hz, 1H), 6.98 (s, 1H), 5.08 – 4.95 (m, 2H), 3.39 (s, 3H), 1.49 (s, 9H); ^13^C NMR (126 MHz, CDCl_3_): *δ* 157.5, 152.3, 143.0, 136.3, 131.2, 128.6, 128.2, 124.9, 124.7, 124.6, 122.0, 115.1, 114.2, 111.9, 91.5, 81.4, 70.1, 43.4, 28.3; LRMS (ESI): *m*/*z* 399.0 [M + H]^+^; HRMS (ESI): calculated for C_21_H_23_N_2_O_4_S [M + H]^+^: 399.1373, found: 399.1383.

4,5-Dimethyl-8H-5λ^4^-isochromeno[3,4-*c*][1,2]benzothiazine 5-oxide (**3la**): yellow solid; m.p.: 109–111 °C; ^1^H NMR (600 MHz, CDCl_3_): *δ* 7.90 (d, *J* = 6.5 Hz, 1H), 7.53 (d, *J* = 6.7 Hz, 1H), 7.44 (dd, *J* = 8.3, 7.3 Hz, 1H), 7.28 (td, *J* = 7.7, 1.6 Hz, 1H), 7.18 (dd, *J* = 7.4, 0.8 Hz, 1H), 7.13 (td, *J* = 7.4, 1.1 Hz, 1H), 7.10 (dt, *J* = 7.3, 1.0 Hz, 1H), 5.12–5.02 (m, 2H), 3.43 (s, 3H), 2.76 (s, 3H); ^13^C NMR (126 MHz, CDCl_3_): *δ* 156.2, 136.6, 135.4, 132.5, 131.7, 128.9, 128.1, 127.6, 125.0, 124.6, 123.0, 122.2, 120.0, 91.2, 70.2, 46.6, 21.1; LRMS (ESI): *m*/*z* 298.1 [M + H]^+^; HRMS (ESI): calculated for C_17_H_16_NO_2_S [M + H]^+^: 298.0896, found: 298.0900.

4-Bromo-5-methyl-8H-5λ^4^-isochromeno[3,4-*c*][1,2]benzothiazine 5-oxide (**3ma**): yellow solid; m.p.: 101–103 °C; ^1^H NMR (500 MHz, CDCl_3_): *δ* 8.07 (dd, *J* = 8.4, 1.1 Hz, 1H), 7.53–7.42 (m, 2H), 7.37 (t, *J* = 8.0 Hz, 1H), 7.31–7.25 (m, 1H), 7.19 (d, *J* = 7.6 Hz, 1H), 7.15 (t, *J* = 6.8 Hz, 1H), 5.19–5.02 (m, 2H), 3.89 (s, 3H); ^13^C NMR (151 MHz, CDCl_3_): *δ* 156.5, 138.5, 132.8, 131.1, 129.7, 128.9, 128.2, 125.2, 125.0, 124.6, 122.3, 120.9, 118.2, 91.2, 70.3, 49.2; LRMS (ESI): *m*/*z* 360.9 [M + H]^+^; HRMS (ESI): calculated for C_16_H_13_BrNO_2_S [M + H]^+^: 361.9845, found: 361.9854.

3-Bromo-5-methyl-8H-5λ^4^-isochromeno[3,4-*c*][1,2]benzothiazine 5-oxide (**3na**): yellow solid; m.p.: 120–122 °C; ^1^H NMR (600 MHz, CDCl_3_): *δ* 7.95 (d, *J* = 8.9 Hz, 1H), 7.88 (d, *J* = 2.1 Hz, 1H), 7.63 (dd, *J* = 8.9, 2.1 Hz, 1H), 7.52 (d, *J* = 8.3 Hz, 1H), 7.31 (td, *J* = 7.5, 1.7 Hz, 1H), 7.21–7.12 (m, 2H), 5.15–5.01 (m, 2H), 3.52 (s, 3H); ^13^C NMR (151 MHz, CDCl_3_): *δ* 157.3, 135.9, 133.7, 130.7, 128.7, 128.3, 126.2, 125.5, 125.1, 125.1, 122.1, 120.9, 116.2, 91.9, 70.3, 42.9; LRMS (ESI): *m*/*z* 361.2 [M + H]^+^; HRMS (ESI): calculated for C_16_H_13_BrNO_2_S [M + H]^+^: 361.9845, found: 361.9843.

3-Methoxy-5-methyl-8H-5λ^4^-isochromeno[3,4-*c*][1,2]benzothiazine 5-oxide (**3oa**) and 1-Methoxy-5-methyl-8H-5λ^4^-isochromeno[3,4-*c*][1,2]benzothiazine 5-oxide (**3oa’**): pale yellow solid; ^1^H NMR (500 MHz, CDCl_3_) for the mixtures: *δ* 8.00 (d, *J* = 9.7 Hz, 1H), 7.54 (d, *J* = 7.9 Hz, 1H), 7.46 (d, *J* = 5.8 Hz, 1H), 7.41–7.33 (m, 5H), 7.30 (t, *J* = 7.4 Hz, 1H), 7.23–7.16 (m, 6H), 7.13 (d, *J* = 6.2 Hz, 7H), 7.08 (t, *J* = 7.2 Hz, 3H), 6.90 (d, *J* = 7.9 Hz, 1H), 6.85 (d, *J* = 7.8 Hz, 3H), 5.16–4.96 (m, 7H), 3.89 (s, 3H), 3.77 (s, 8H), 3.64 (s, 8H), 3.45 (s, 3H); ^13^C NMR (126 MHz, CDCl_3_) for the mixtures: *δ* 157.5, 154.8, 132.2, 132.0, 130.8, 128.2, 128.0, 127.6, 126.3, 125.8, 124.9, 124.5, 124.3, 124.1, 123.8, 123.2, 123.1, 123.1, 122.0, 121.4, 121.3, 120.0, 115.7, 115.1, 113.6, 113.5, 104.8, 89.4, 69.8, 69.6, 55.5, 54.8, 42.4, 42.0; LRMS (ESI): *m*/*z* 314.2 [M + H]^+^; HRMS (ESI): calculated for C_17_H_16_NO_3_S [M + H]^+^: 314.0845, found: 314.0837.

8-Methyl-5H-8λ^4^-isochromeno[3,4-*c*]naphtho[2,3-*e*][1,2]thiazine 8-oxide (**3pa**): yellow solid; m.p.: 98–100 °C; ^1^H NMR (600 MHz, CDCl_3_): *δ* 8.40 (s, 2H), 7.92 (d, *J* = 8.3 Hz, 1H), 7.82 (d, *J* = 8.4 Hz, 1H), 7.77 (d, *J* = 7.8 Hz, 1H), 7.61–7.55 (m, 1H), 7.46 (t, *J* = 7.5 Hz, 1H), 7.37 (t, *J* = 8.2 Hz, 1H), 7.23 (d, *J* = 7.1 Hz, 1H), 7.19 (t, *J* = 6.5 Hz, 1H), 5.11 (s, 2H), 3.41 (s, 3H); ^13^C NMR (126 MHz, CDCl_3_): *δ* 156.4, 135.8, 131.7, 130.2, 130.0, 129.3, 128.9, 128.8, 128.2, 127.8, 126.0, 125.1, 124.8, 124.6, 123.3, 121.9, 121.8, 91.8, 70.2, 42.5; LRMS (ESI): *m*/*z* 334.0 [M + H]^+^; HRMS (ESI): calculated for C_20_H_16_NO_2_S [M + H]^+^: 334.0896, found: 334.0894.

5-Ethyl-8H-5λ^4^-isochromeno[3,4-*c*][1,2]benzothiazine 5-oxide (**3qa**): yellow-green solid; m.p.: 77–79 °C; ^1^H NMR (500 MHz, CDCl_3_): *δ* 8.07 (d, *J* = 8.4 Hz, 1H), 7.72 (dd, *J* = 8.0, 1.4 Hz, 1H), 7.60 (d, *J* = 6.8 Hz, 1H), 7.59–7.55 (m, 1H), 7.34–7.27 (m, 2H), 7.19 (d, *J* = 5.9 Hz, 1H), 7.14 (td, *J* = 7.4, 1.2 Hz, 1H), 5.09 (q, *J* = 12.2 Hz, 2H), 3.60 (ddt, *J* = 32.6, 14.5, 7.3 Hz, 2H), 1.36 (t, *J* = 7.3 Hz, 3H); ^13^C NMR (126 MHz, CDCl_3_): *δ* 157.7, 136.0, 133.0, 131.4, 128.8, 128.2, 125.0, 124.7, 124.6, 124.2, 123.8, 122.0, 117.2, 91.3, 70.2, 49.3, 7.9; LRMS (ESI): *m*/*z* 298.1 [M + H]^+^; HRMS (ESI): calculated for C_17_H_16_NO_2_S [M + H]^+^: 298.0896, found: 298.0904.

5-Cyclopropyl-8H-5λ^4^-isochromeno[3,4-*c*][1,2]benzothiazine 5-oxide (**3ra**): yellow oil; ^1^H NMR (600 MHz, CDCl_3_): *δ* 8.09 (d, *J* = 8.3 Hz, 1H), 7.87 (d, *J* = 8.0 Hz, 1H), 7.62 (d, *J* = 7.8 Hz, 1H), 7.56 (t, *J* = 7.7 Hz, 1H), 7.33–7.28 (m, 2H), 7.19 (d, *J* = 7.3 Hz, 1H), 7.14 (t, *J* = 7.4 Hz, 1H), 5.17–4.99 (m, 2H), 2.88 (tt, *J* = 8.0, 4.7 Hz, 1H), 1.78 – 1.70 (m, 1H), 1.57–1.49 (m, 1H), 1.42 (dt, *J* = 14.9, 7.8 Hz, 1H), 1.31–1.26 (m, 1H); ^13^C NMR (151 MHz, CDCl_3_): *δ* 157.4, 135.2, 132.5, 131.3, 128.8, 128.1, 125.0, 124.7, 124.4, 124.1, 123.3, 122.3, 120.5, 91.8, 70.2, 30.7, 7.0, 4.6; LRMS (ESI): *m*/*z* 310.1 [M + H]^+^; HRMS (ESI): calculated for C_18_H_16_NO_2_S [M + H]^+^: 310.0896, found: 310.0904.

5-(Chloromethyl)-8H-5λ^4^-isochromeno[3,4-*c*][1,2]benzothiazine 5-oxide (**3sa**): yellow-green solid; m.p.: 194–196 °C; ^1^H NMR (600 MHz, CDCl_3_): *δ* 8.06 (d, *J* = 8.4 Hz, 1H), 7.87 (dd, *J* = 8.1, 1.4 Hz, 1H), 7.66–7.62 (m, 1H), 7.61 (d, *J* = 7.4 Hz, 1H), 7.38–7.34 (m, 1H), 7.32 (td, *J* = 7.5, 1.7 Hz, 1H), 7.21 (dd, *J* = 7.6, 1.6 Hz, 1H), 7.17 (td, *J* = 7.3, 1.1 Hz, 1H), 5.09 (q, *J* = 12.3 Hz, 2H), 4.89–4.75 (m, 2H); ^13^C NMR (126 MHz, CDCl_3_): *δ* 157.3, 137.3, 134.3, 130.8, 128.9, 128.3, 125.8, 125.1, 125.1, 124.7, 124.4, 122.1, 114.6, 91.7, 70.4, 58.6; LRMS (ESI): *m*/*z* 317.9 [M + H]^+^; HRMS (ESI): calculated for C_16_H_13_ClNO_2_S [M + H]^+^: 318.0350, found: 318.0345.

2-(5-Oxido-8H-5λ^4^-isochromeno[3,4-*c*][1,2]benzothiazin-5-yl)ethanol (**3ta**): yellow-green solid; m.p.: 143–145 °C; ^1^H NMR (600 MHz, CDCl_3_): *δ* 8.08 (d, *J* = 8.3 Hz, 1H), 7.80 (dd, *J* = 8.1, 1.3 Hz, 1H), 7.64–7.56 (m, 2H), 7.36–7.29 (m, 2H), 7.21–7.14 (m, 2H), 5.19–5.03 (m, 2H), 4.18–4.07 (m, 2H), 3.95 – 3.83 (m, 2H), 3.26 (s, 1H); ^13^C NMR (151 MHz, CDCl_3_): *δ* 156.5, 135.1, 132.9, 130.9, 129.7, 128.6, 128.1, 126.4, 125.0, 124.4, 123.4, 122.2, 118.8, 92.2, 70.2, 56.3, 56.2; LRMS (ESI): *m*/*z* 314.1 [M + H]^+^; HRMS (ESI): calculated for C_17_H_16_NO_3_S [M + H]^+^: 314.0845, found: 314.0842.

5-Phenyl-8H-5λ^4^-isochromeno[3,4-*c*][1,2]benzothiazine 5-oxide (**3ua**): yellow solid; m.p.: 102–103 °C; ^1^H NMR (600 MHz, CDCl_3_): *δ* 8.13 (d, *J* = 8.4 Hz, 1H), 8.07 (d, *J* = 7.8 Hz, 2H), 7.71 (t, *J* = 7.4 Hz, 1H), 7.67 (d, *J* = 7.8 Hz, 1H), 7.62 (t, *J* = 7.7 Hz, 2H), 7.51 (t, *J* = 7.7 Hz, 1H), 7.34 (t, *J* = 7.6 Hz, 1H), 7.22 (t, *J* = 8.0 Hz, 2H), 7.19–7.13 (m, 2H), 5.24–5.10 (m, 2H); ^13^C NMR (151 MHz, CDCl_3_): *δ* 157.4, 136.8, 134.6, 134.3, 132.2, 131.3, 129.8, 129.3, 128.9, 128.2, 125.0, 124.9, 124.7, 124.2, 124.1, 122.5, 121.2, 92.1, 70.4; LRMS (ESI): *m*/*z* 346.0 [M + H]^+^; HRMS (ESI): calculated for C_21_H_16_NO_2_S [M + H]^+^: 346.0896, found: 346.0899.

5-Benzyl-8H-5λ^4^-isochromeno[3,4-*c*][1,2]benzothiazine 5-oxide (**3va**): yellow-green solid; m.p.: 66–68 °C; ^1^H NMR (600 MHz, CDCl_3_): *δ* 7.99 (dd, *J* = 8.1, 1.1 Hz, 1H), 7.75 (dd, *J* = 7.8, 1.4 Hz, 1H), 7.65 (d, *J* = 8.9 Hz, 1H), 7.59 – 7.53 (m, 1H), 7.38 (td, *J* = 7.5, 1.1 Hz, 1H), 7.32 (t, *J* = 6.9 Hz, 1H), 7.30–7.26 (m, 2H), 7.25–7.22 (m, 1H), 7.18 (td, *J* = 7.4, 1.1 Hz, 1H), 7.16–7.12 (m, 3H), 5.33 (d, *J* = 15.5 Hz, 1H), 5.08 (d, *J* = 12.1 Hz, 1H), 4.98 (d, *J* = 15.5 Hz, 1H), 4.62 (d, *J* = 12.1 Hz, 1H); ^13^C NMR (126 MHz, CDCl_3_): *δ* 147.2, 137.0, 133.9, 131.4, 130.6, 129.0, 128.8, 128.5, 128.3, 127.8, 127.4, 126.5, 125.8, 125.4, 125.3, 124.9, 123.5, 97.0, 70.4, 54.2; LRMS (ESI): *m*/*z* 360.0 [M + H]^+^; HRMS (ESI): calculated for C_22_H_18_NO_2_S [M + H]^+^: 360.1053, found: 360.1053.

5,10-Dimethyl-8H-5λ^4^-isochromeno[3,4-*c*][1,2]benzothiazine 5-oxide (**3ab**): yellow-green solid; m.p.: 152–154 °C; ^1^H NMR (600 MHz, CDCl_3_): *δ* 8.05 (d, *J* = 8.4 Hz, 1H), 7.77 (d, *J* = 7.7 Hz, 1H), 7.59–7.54 (m, 1H), 7.49 (d, *J* = 7.9 Hz, 1H), 7.32 (t, *J* = 7.6 Hz, 1H), 7.12 (dd, *J* = 8.0, 1.8 Hz, 1H), 7.01 (s, 1H), 5.12–5.00 (m, 2H), 3.49 (s, 3H), 2.36 (s, 3H); ^13^C NMR (151 MHz, CDCl_3_): *δ* 156.8, 135.1, 134.6, 132.8, 129.0, 128.8, 128.4, 125.7, 124.5, 124.3, 123.2, 122.2, 119.9, 91.9, 70.3, 43.0, 21.1.; LRMS (ESI): *m*/*z* 298.1 [M + H]^+^; HRMS (ESI): calculated for C_17_H_16_NO_2_S [M + H]^+^: 298.0896, found: 298.0902.

10-Methoxy-5-methyl-8H-5λ^4^-isochromeno[3,4-*c*][1,2]benzothiazine 5-oxide (**3ac**): yellow-green solid; m.p.: 108–110 °C; ^1^H NMR (600 MHz, CDCl_3_): *δ* 8.02 (d, *J* = 8.4 Hz, 1H), 7.76 (d, *J* = 8.1 Hz, 1H), 7.56 (t, *J* = 7.7 Hz, 1H), 7.51 (d, *J* = 8.5 Hz, 1H), 7.31 (t, *J* = 7.6 Hz, 1H), 6.87 (dd, *J* = 8.6, 2.7 Hz, 1H), 6.76 (d, *J* = 2.7 Hz, 1H), 5.05 (q, *J* = 12.3 Hz, 2H), 3.82 (s, 3H), 3.50 (s, 3H); ^13^C NMR (151 MHz, CDCl_3_): *δ* 157.2, 156.2, 135.0, 132.8, 130.5, 124.4, 124.2, 123.9, 123.5, 123.1, 119.8, 113.5, 110.9, 91.7, 70.2, 55.6, 42.9; LRMS (ESI): *m*/*z* 314.1 [M + H]^+^; HRMS (ESI): calculated for C_17_H_16_NO_3_S [M + H]^+^: 314.0845, found: 314.0847.

11-Methoxy-5-methyl-8H-5λ^4^-isochromeno[3,4-*c*][1,2]benzothiazine 5-oxide (**3ad**): yellow solid; m.p.: 209–211 °C; ^1^H NMR (600 MHz, CDCl_3_): *δ* 8.06 (d, *J* = 8.3 Hz, 1H), 7.74 (d, *J* = 6.7 Hz, 1H), 7.60–7.51 (m, 1H), 7.32–7.27 (m, 1H), 7.12 (d, *J* = 2.5 Hz, 1H), 7.08 (d, *J* = 8.2 Hz, 1H), 6.66 (dd, *J* = 8.2, 2.5 Hz, 1H), 5.07–4.95 (m, 2H), 3.78 (s, 3H), 3.46 (s, 3H); ^13^C NMR (151 MHz, CDCl_3_): *δ* 159.7, 157.5, 135.0, 133.0, 132.6, 125.9, 124.5, 124.4, 123.2, 121.4, 120.0, 109.4, 108.7, 91.9, 69.9, 55.5, 42.9; LRMS (ESI): *m*/*z* 314.0 [M + H]^+^; HRMS (ESI): calculated for C_17_H_16_NO_3_S [M + H]^+^: 314.0845, found: 314.0842.

10-Chloro-5-methyl-8H-5λ^4^-isochromeno[3,4-*c*][1,2]benzothiazine 5-oxide (**3ae**): yellow solid; m.p.: 180–182 °C; ^1^H NMR (500 MHz, CDCl_3_): *δ* 7.98 (d, *J* = 7.3 Hz, 1H), 7.78 (dd, *J* = 8.1, 1.3 Hz, 1H), 7.60–7.56 (m, 1H), 7.51 (d, *J* = 8.3 Hz, 1H), 7.37–7.31 (m, 1H), 7.28–7.24 (m, 1H), 7.17 (d, J = 2.2 Hz, 1H), 5.11–4.95 (m, 2H), 3.51 (s, 3H); ^13^C NMR (126 MHz, CDCl_3_): *δ* 157.3, 134.7, 133.0, 130.4, 129.9, 129.8, 128.2, 125.1, 124.6, 124.3, 123.3, 123.3, 120.1, 91.3, 69.6, 43.0; LRMS (ESI): *m*/*z* 318.1 [M + H]^+^; HRMS (ESI): calculated for C_16_H_13_ClNO_2_S [M + H]^+^: 318.0350, found: 318.0359.

11-Chloro-5-methyl-8H-5λ^4^-isochromeno[3,4-*c*][1,2]benzothiazine 5-oxide (**3af**): yellow-green solid; m.p.: 66–68 °C; ^1^H NMR (600 MHz, CDCl_3_): *δ* 8.02 (d, *J* = 8.2 Hz, 1H), 7.78 (dd, *J* = 8.1, 1.3 Hz, 1H), 7.65–7.60 (m, 1H), 7.56 (s, 1H), 7.38–7.33 (m, 1H), 7.11 (d, *J* = 1.3 Hz, 2H), 5.13–4.95 (m, 2H), 3.50 (s, 3H); ^13^C NMR (151 MHz, CDCl_3_): *δ* 157.7, 134.6, 134.2, 133.3, 133.2, 126.9, 126.2, 124.8, 124.5, 124.2, 123.3, 121.9, 120.1, 91.2, 69.7, 43.0; LRMS (ESI): *m*/*z* 318.1 [M + H]^+^; HRMS (ESI): calculated for C_16_H_13_ClNO_2_S [M + H]^+^: 318.0350, found: 318.0349.

10-Fluoro-5-methyl-8H-5λ^4^-isochromeno[3,4-*c*][1,2]benzothiazine 5-oxide (**3ag**): yellow-green solid; m.p.: 77–79 °C; ^1^H NMR (600 MHz, CDCl_3_): *δ* 8.00 (d, *J* = 8.4 Hz, 1H), 7.78 (dd, *J* = 8.1, 1.3 Hz, 1H), 7.60–7.55 (m, 1H), 7.53 (dd, *J* = 8.6, 5.2 Hz, 1H), 7.40–7.30 (m, 1H), 7.00 (td, *J* = 8.6, 2.8 Hz, 1H), 6.91 (dd, *J* = 8.4, 2.7 Hz, 1H), 5.12–4.97 (m, 2H), 3.52 (s, 3H); ^13^C NMR (151 MHz, CDCl_3_): *δ* 161.1, 159.5, 156.8, 134.7, 132.9, 130.7 (d, *J* = 7.0 Hz), 127.3 (d, *J* = 3.2 Hz), 124.4 (d, *J* = 42.3 Hz), 123.6 (d, *J* = 7.7 Hz), 123.2, 120.1, 114.9 (d, *J* = 21.4 Hz), 112.3 (d, *J* = 22.3 Hz), 91.4, 69.6 (d, *J* = 2.1 Hz), 42.9; LRMS (ESI): *m*/*z* 302.1 [M + H]^+^; HRMS (ESI): calculated for C_16_H_13_FNO_2_S [M + H]^+^: 302.0646, found: 302.0649.

11-Fluoro-5-methyl-8H-5λ^4^-isochromeno[3,4-*c*][1,2]benzothiazine 5-oxide (**3ah**): pale yellow solid; m.p.: 91–93 °C; ^1^H NMR (600 MHz, CDCl_3_): *δ* 8.02 (d, *J* = 8.9 Hz, 1H), 7.78 (dd, *J* = 8.1, 1.3 Hz, 1H), 7.67–7.57 (m, 1H), 7.38–7.32 (m, 1H), 7.28 (dd, *J* = 10.6, 2.5 Hz, 1H), 7.14 (dd, *J* = 8.3, 5.7 Hz, 1H), 6.82 (td, *J* = 8.4, 2.5 Hz, 1H), 5.12–4.97 (m, 2H), 3.51 (s, 3H); ^13^C NMR (151 MHz, CDCl_3_): *δ* 163.7, 162.1, 157.6, 134.5, 133.3 (d, *J* = 9.2 Hz), 133.1, 126.3 (d, *J* = 9.3 Hz), 124.6, 124.1 (d, *J* = 3.6 Hz), 123.2, 120.0, 111.0 (d, *J* = 22.3 Hz), 109.0 (d, *J* = 24.1 Hz), 91.3 (d, *J* = 2.2 Hz), 69.6, 42.9; LRMS (ESI): *m*/*z* 302.0 [M + H]^+^; HRMS (ESI): calculated for C_16_H_13_FNO_2_S [M + H]^+^: 302.0646, found: 302.0645.

5-Methyl-10-(trifluoromethyl)-8H-5λ^4^-isochromeno[3,4-*c*][1,2]benzothiazine 5-oxide (**3ai**): yellow solid; m.p.: 191–193 °C; ^1^H NMR (600 MHz, CDCl_3_): *δ* 8.01 (d, *J* = 8.3 Hz, 1H), 7.80 (dd, *J* = 8.0, 1.3 Hz, 1H), 7.68 (d, *J* = 8.2 Hz, 1H), 7.63–7.58 (m, 1H), 7.56–7.52 (m, 1H), 7.44 (s, 1H), 7.42–7.35 (m, 1H), 5.21–5.02 (m, 2H), 3.53 (s, 3H); ^13^C NMR (151 MHz, CDCl_3_): *δ* 158.3, 135.0, 134.5, 133.2, 128.7, 126.4 (q, *J* = 32.6 Hz), 125.3 (d, *J* = 3.6 Hz), 124.9, 124.3, 123.3, 122.0 (d, *J* = 3.9 Hz), 121.9, 120.2, 91.4, 69.7, 43.0; LRMS (ESI): *m*/*z* 352.0 [M + H]^+^; HRMS (ESI): calculated for C_17_H_13_F_3_NO_2_S [M + H]^+^: 352.0614, found: 352.0617.

5-Methyl-11-(trifluoromethyl)-8H-5λ^4^-isochromeno[3,4-*c*][1,2]benzothiazine 5-oxide (**3aj**): yellow solid; m.p.: 150–152 °C; ^1^H NMR (600 MHz, CDCl_3_): *δ* 8.00 (d, *J* = 8.3 Hz, 1H), 7.83 (s, 1H), 7.81 (dd, *J* = 8.1, 1.3 Hz, 1H), 7.67–7.61 (m, 1H), 7.42–7.36 (m, 2H), 7.30 (d, *J* = 7.8 Hz, 1H), 5.20–5.04 (m, 2H), 3.52 (s, 3H); ^13^C NMR (151 MHz, CDCl_3_): *δ* 157.8, 134.5, 133.5, 132.2, 131.9, 131.0–130.3 (m), 125.5, 125.0, 124.0, 123.4, 121.5–121.4 (m), 120.2, 118.6 (q, *J* = 3.9 Hz), 91.3, 69.7, 43.0; LRMS (ESI): *m*/*z* 352.0 [M + H]^+^; HRMS (ESI): calculated for C_17_H_13_F_3_NO_2_S [M + H]^+^: 352.0614, found: 352.0618.

#### 3.2.5. Mechanistic Investigations

**KIE Experiment.** To a 15 mL vial was added *d*^1^-**1a** (23.43 mg, 0.15 mmol), **2a** (54.01 mg, 0.165 mmol), [Cp*RhCl_2_]_2_ (4.64 mg, 5 mol%), AgOPiv (6.27 mg, 20 mol%) under air. Trifluoroethanol (TFE, 2.5 mL) was added subsequently. The resulting mixture was stirred at ambient temperature for 6 h. Then, the mixture was filtered through a celite pad and washed with DCM (10 mL × 3). The combined organic layer was concentrated under vacuo, and the residue was purified by silica gel chromatography eluting with DCM/MeOH from 50:1 to 30:1 to give the mixture of **3aa** and *d*^1^-**3aa**. The ratio of two products was determined by ^1^H NMR to give an intramolecular kinetic isotopic effect (KIE) *k*H/*k*D = 0.47. (see Supporting Information).

**H/D Exchange Experiment.** A mixture of **1a** (23.43 mg, 0.15 mmol), [Cp*RhCl_2_]_2_ (4.64 mg, 5 mol%), AgOPiv (6.27 mg, 20 mol%) and *d*^3^-TFE was added into a vial under air. The resulting mixture was stirred at room temperature for 18 h. Then, the mixture was filtered through a celite pad and washed with DCM (10 mL × 3). The combined organic layer was concentrated under vacuo, and the residue was purified by silica gel chromatography eluting with DCM/MeOH 10:1 to give the product *d*^2^-**1a**. H/D exchange occurred at the *o*-position of *S*-phenylsulfoximine (91% D). (see Supporting Information).

**Competition Experiment.** To a mixture of **1c** (27.79 mg, 0.15 mmol), **1i** (29.59 mg, 0.15 mmol), **2a** (54.01 mg, 0.165 mmol), [Cp*RhCl_2_]_2_ (4.64 mg, 5 mol%), and AgOPiv (6.27 mg, 20 mol%) was added TFE (2.5 mL) under air. The resulting mixture was stirred at room temperature for 18 h. After the reaction was completed, the mixture was filtered through a celite pad and washed with DCM (10 mL × 3). The combined organic layer was concentrated under vacuo and the residue was purified by silica gel chromatography eluting with DCM/MeOH from 100:1 to 30:1 to give the isolated products **3ca** and **3ia**. The ratio was calculated according to the moles of products.

## 4. Conclusions

In summary, we have developed a sulfoximie-assisted Rh(III)-catalyzed C–H activation and intramolecular annulation. In this strategy, fused isochromeno-1,2-benzothiazines was unprecedentedly synthesized, and the desired products could be yields in good to excellent yields. Most importantly, it is a redox-neutral process that can be conducted under room temperature and the strategy features broad generality and versatility.

## Figures and Tables

**Table 1:**
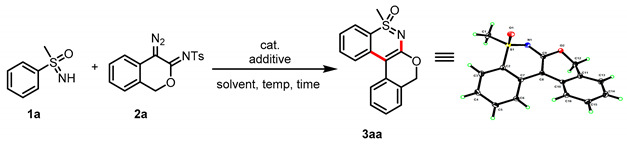
Optimization of reaction conditions *^a^*. TFE: trifluoroethanol.

Entry	Catalyst (mol%)	Additive (mol%)	Solvent	Temp (°C)	Yield of 3aa (%) *^b^*
1	[Cp*RhCl_2_]_2_ (10)	AgSbF_6_ (40)	DCE	100	ND
2	[Cp*RhCl_2_]_2_ (10)	AgSbF_6_ (40)	DCE	80	16
3	[Cp*RhCl_2_]_2_ (10)	AgSbF_6_ (40)	HFIP	80	trace
4	[Cp*RhCl_2_]_2_ (10)	AgSbF_6_ (40)	DCE	rt	trace
5	[Cp*RhCl_2_]_2_ (10)	AgSbF_6_ (40)	HFIP	rt	47
6	[Cp*RhCl_2_]_2_ (10)	AgSbF_6_ (40)	TFE	rt	75
7	[Cp*RhCl_2_]_2_ (10)	AgSbF_6_ (40)	EtOH	rt	57
8	[Cp*RhCl_2_]_2_ (10)	AgSbF_6_ (40)	THF	rt	ND
9	[Cp*RhCl_2_]_2_ (10)	AgSbF_6_ (40)	DME	rt	trace
10	[Cp*RhCl_2_]_2_ (10)	AgOAc (40)	TFE	rt	69
11	[Cp*RhCl_2_]_2_ (10)	AgNTf_2_ (40)	TFE	rt	27
12	[Cp*RhCl_2_]_2_ (10)	AgBF_4_ (40)	TFE	rt	ND
13	[Cp*RhCl_2_]_2_ (10)	AgOPiv (40)	TFE	rt	99 (92) *^c^*
14	[Cp*RhCl_2_]_2_ (20)	AgOPiv (40)	TFE	rt	47
15	[Cp*RhCl_2_]_2_ (5)	AgOPiv (40)	TFE	rt	77
16	[Cp*RhCl_2_]_2_ (5)	AgOPiv (20)	TFE	rt	97 (93) *^c^*
17	[Cp*RhCl_2_]_2_ (2.5)	AgOPiv (10)	TFE	rt	87
18 *^d^*	[Cp*RhCl_2_]_2_ (10)	AgOPiv (40)	TFE	rt	90 (89) *^c^*
19 *^e^*	[Cp*RhCl_2_]_2_ (10)	AgOPiv (40)	TFE	rt	82
20	[Cp*RuCl_2_]_2_ (10)	AgOPiv (40)	TFE	rt	trace
21	[Cp*lrCl_2_]_2_ (10)	AgOPiv (40)	TFE	rt	85
22	-	AgOPiv (40)	TFE	rt	ND

*^a^* Reaction conditions: **1a** (0.15 mmol), **2a** (0.165 mmol), catalyst and additive in solvent (2.5 mL) under air. *^b^* Determined by ^1^H NMR spectroscopy using 1,3,5-trimethoxybenzene as an internal standard. *^c^* Isolated yield in parentheses. *^d^* Under an Argon atmosphere. *^e^* The reaction time was shortened to 12 h.

**Table 2:** Substrate scope of sulfoximines *^a^*.

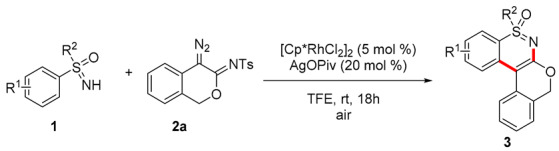
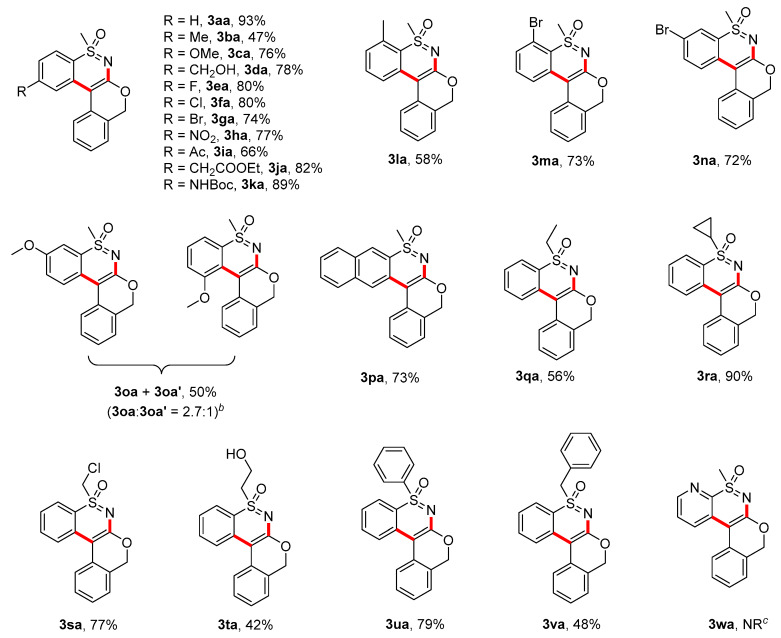

*^a^* Reaction conditions: **1** (0.15 mmol), **2a** (0.165 mmol), [Cp*RhCl_2_]_2_ (5 mol%) and AgOPiv (20 mol%) in TFE (2.5 mL) under air at room temperature for 18 h. All listed yields are isolated ones. *^b^* Determined by ^1^H NMR spectroscopy. *^c^* NR means No Reaction.

**Table 3:** Substrate scope of 4-diazoisochroman-3-imines *^a^*.

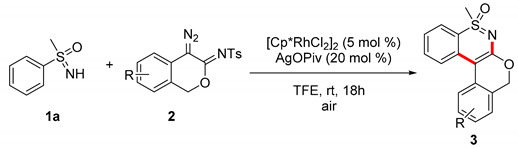
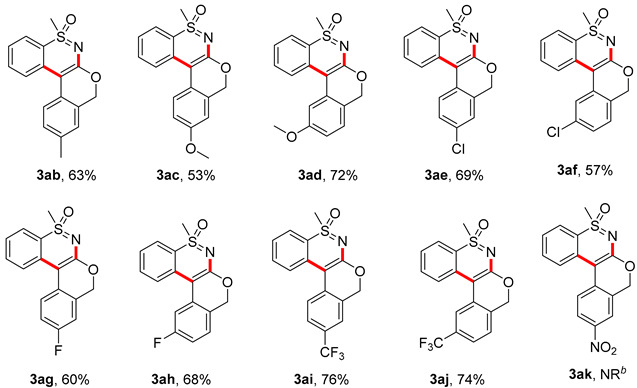

*^a^* Reaction conditions: **1a** (0.15 mmol), **2** (0.165 mmol), [Cp*RhCl_2_]_2_ (5 mol%) and AgOPiv (20 mol%) in TFE (2.5 mL) under air at room temperature for 18 h. All listed yields are isolated ones. *^b^* NR means No Reaction.

## Supplementary Materials

Supplementary File 1
