# Supplementary material for: Sulfoximines-Assisted Rh(III)-Catalyzed C–H Activation and Intramolecular Annulation for the Synthesis of Fused Isochromeno-1,2-Benzothiazines Scaffolds under Room Temperature
Source: Molecules. 2020 May 28;25(11):2515. doi: 10.3390/molecules25112515 (PMC7321204; doi:10.3390/molecules25112515)
Supplement: Supplementary File 1 [file molecules-25-02515-s001.pdf]

Supporting Information

Sulfoximines Assisted Rh(III)-Catalyzed C-H Activation and Intramolecular Annulation for the Synthesis of Fused Isochromeno-1,2-Benzothiazines Scaffolds under Room Temperature

Bao Wang [1, 2, 3], Xu Han [1, 2], Jian Li [1], Chunpu Li [1, 2,*] and Hong Liu [1, 2, 3,*]

[1]State Key Laboratory of Drug Research and CAS Key Laboratory of Receptor Research, Shanghai Institute of Materia Medica, Chinese Academy of Sciences, 555 Zu Chong Zhi Road, Shanghai, 201203, China

[2]University of Chinese Academy of Sciences, No. 19A Yuquan Road, Beijing 100049, China

[3]School of Life Science and Technology, ShanghaiTech University, 100 Haike Road, Shanghai 201210, China

Correspondence: lichunpu@simm.ac.cn (C.L.) and hliu@simm.ac.cn (H.L.)

Table of Contents

1. X-ray crystallography data of 3aa S3

2. Conversion of stereoisomer 1a S5

3. Mechanistic investigations S7

4. NMR spectra data S10

1. X-ray Crystallographic Data of 3aa

1.1 X-ray Single Crystal Diffraction Data of compound 3aa

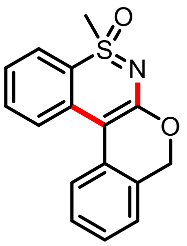

3aa

Sample preparation: To a solution of compound **3aa** (10 mg) dissolved in EtOAc (1.0 mL) was filtered through a nylon-membrane syringe filter (13 mm*0.22 μm, purchased from ANPEL Laboratory Tech. Shanghai, Inc.) and transferred into a clean 2 mL vial. The vial was sealed with a thin layer of parafilm on top of which 3-5 holes was made with a capillary (0.3 mm) to allow the solvent slowly evaporated at room temperature to afford the single crystal **3aa** in 48 hours.

Single crystal structure of 3aa： X-ray crystal structure of **3aa** was determined at 170 K with the ellipsoid contour at 50% probability levels.

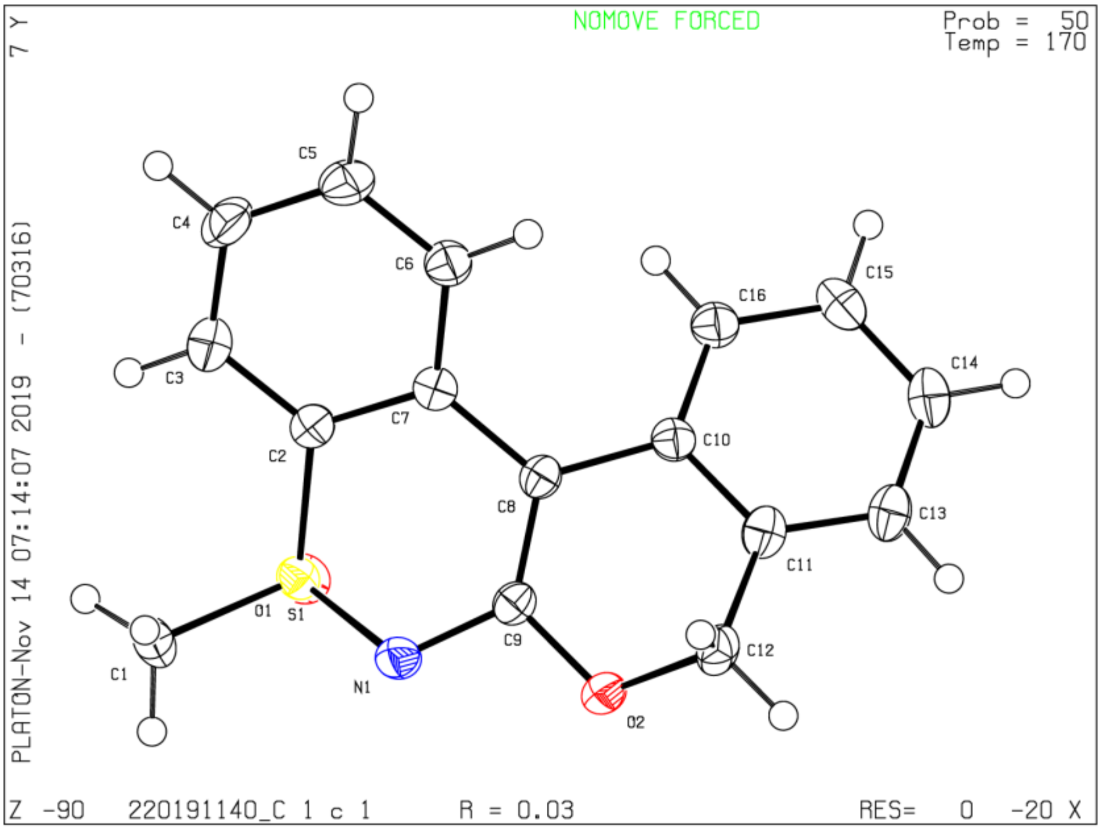

Table 1 Crystal data and structure refinement for 220191140_0m (3aa).

Identification code	220191140_0m
Empirical formula	$C_{16}H_{13}NO_2S$
Formula weight	283.33
Temperature/K	170.0
Crystal system	monoclinic
Space group	Cc
a/Å	14.3267(7)
b/Å	10.2239(6)
c/Å	9.2727(4)
α/°	90
β/°	106.242(2)
γ/°	90
Volume/Å^3	1304.01(12)
Z	4
ρ_{calc}g/cm^3	1.443
μ/mm^{-1}	0.248
F(000)	592.0
Crystal size/mm^3	0.18 × 0.11 × 0.08
Radiation	MoKα (λ = 0.71073)
2Θ range for data collection/°	4.964 to 54.998
Index ranges	$-18 \leq h \leq 18$, $-12 \leq k \leq 13$, $-12 \leq l \leq 11$
Reflections collected	6706
Independent reflections	2590 [R_{int} = 0.0413, R_{sigma} = 0.0517]
Data/restraints/parameters	2590/2/182
Goodness-of-fit on F^2	1.066
Final R indexes [I>=2σ (I)]	R_1 = 0.0344, wR_2 = 0.0804
Final R indexes [all data]	R_1 = 0.0382, wR_2 = 0.0837

Largest diff. peak/hole / e $Å^{-3}$ 0.25/-0.28

Flack parameter -0.01(6)

Crystal structure determination of [220191140_0m] (3aa)

Crystal Data for $C_{16}H_{13}NO_2S$ (M =283.33 g/mol): monoclinic, space group Cc (no. 9), a = 14.3267(7) Å, b = 10.2239(6) Å, c = 9.2727(4) Å, β = 106.242(2)°, V = 1304.01(12) $Å^3$, Z = 4, T = 170.0 K, μ(MoKα) = 0.248 mm^{-1}, $Dcalc$ = 1.443 g/cm^3, 6706 reflections measured (4.964° ≤ 2Θ ≤ 54.998°), 2590 unique (R_{int} = 0.0413, R_{sigma} = 0.0517) which were used in all calculations. The final R_1 was 0.0344 (I > 2σ(I)) and wR_2 was 0.0837 (all data).

2. Conversion of stereoisomer 1a

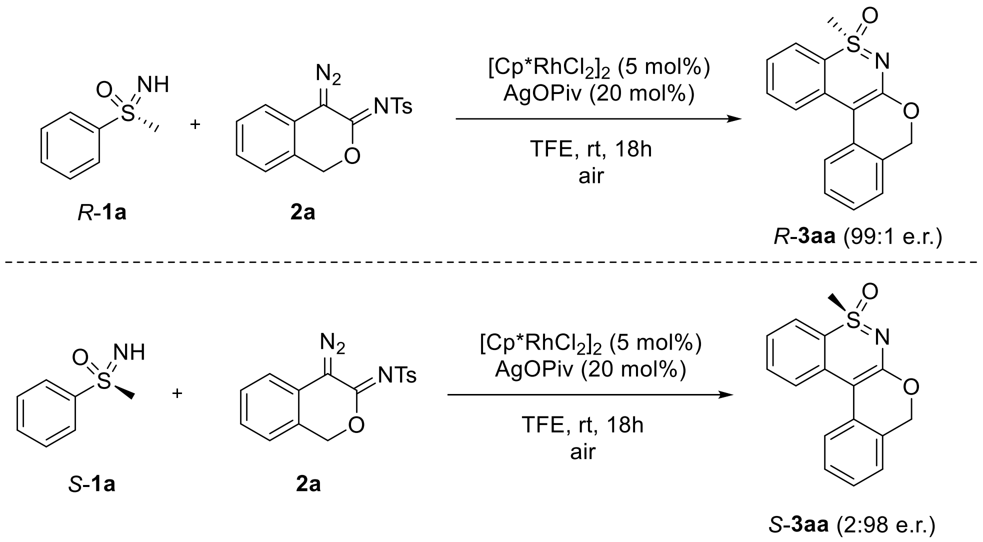

5-methyl-8H-5λ[4]-isochromeno[3,4-c][1,2]benzothiazine 5-oxide (3aa). (*R*)-**3aa** Yellow-green solid, yield 93% (39.4 mg), 99:1 e.r. was determined by chiral HPLC (Chiralcel AD-H, *n*-hexane/*i*-PrOH = 70/30, 0.8 mL/min, 254 nm, 25 °C): t_R (major) = 13.2 min, t_R (minor) = 17.6 min; (*S*)-**3aa**, Yellow-green solid, yield 91% (38.6 mg), 2:98 e.r. was determined by chiral HPLC (Chiralcel AD-H, *n*-hexane/*i*-PrOH = 70/30, 0.8 mL/min, 254 nm, 25 °C): t_R (minor) = 12.0 min, t_R (major) = 17.4 min; ^{1}H NMR (500 MHz, $CDCl_3$): δ 8.07 (d, J = 9.6 Hz, 1H), 7.78 (dd, J = 8.0, 1.3 Hz, 1H), 7.62 – 7.54 (m, 2H), 7.38 – 7.26 (m, 2H), 7.21 – 7.11 (m, 2H), 5.20 – 4.99 (m, 2H), 3.49 (s, 3H); ^{13}C NMR (126 MHz, $CDCl_3$): δ 157.3, 135.0, 132.9, 131.3, 128.8, 128.2, 125.0, 124.8, 124.5, 124.4, 123.2, 122.2, 120.0, 91.9, 70.3, 43.0;

LRMS (ESI): *m/z* 284.1 $[M + H]^+$; HRMS (ESI): calculated for $C_{16}H_{14}NO_2S$ $[M + H]^+$: 284.0740, found: 284.0745.

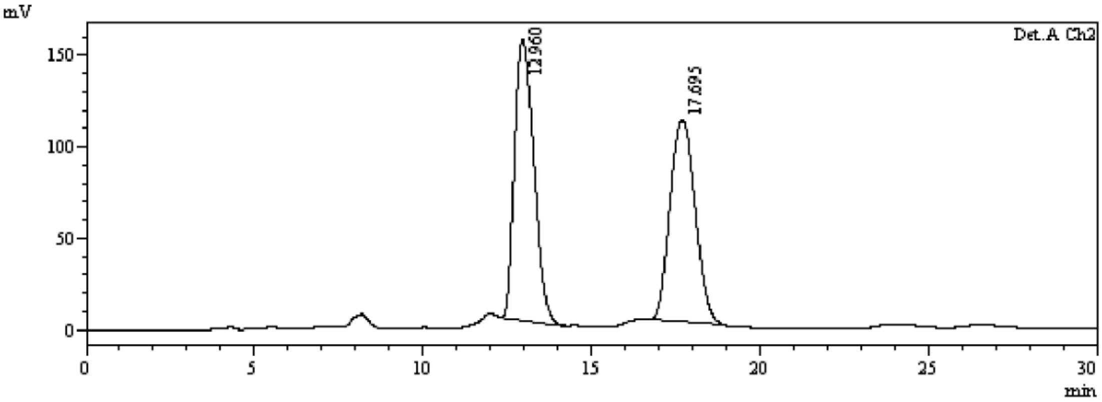

PeakTable

Detector A Ch2 254nm

Peak#	Ret. Time	Area	Height	Area %	Height %
1	12.960	6107921	152959	51.401	58.021
2	17.695	5774872	110667	48.599	41.979
Total		11882793	263626	100.000	100.000

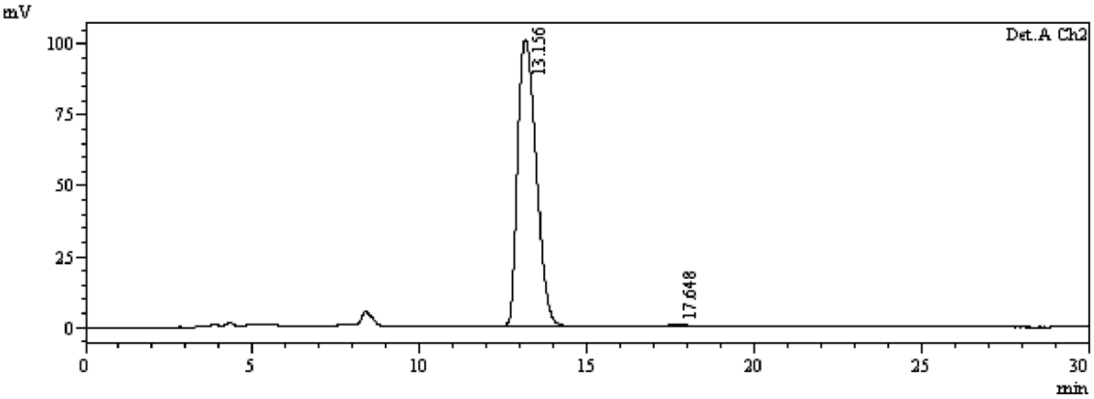

PeakTable

Detector A Ch2 254nm

Peak#	Ret. Time	Area	Height	Area %	Height %
1	13.156	3911997	101074	99.243	99.344
2	17.648	29834	668	0.757	0.656
Total		3941832	101742	100.000	100.000

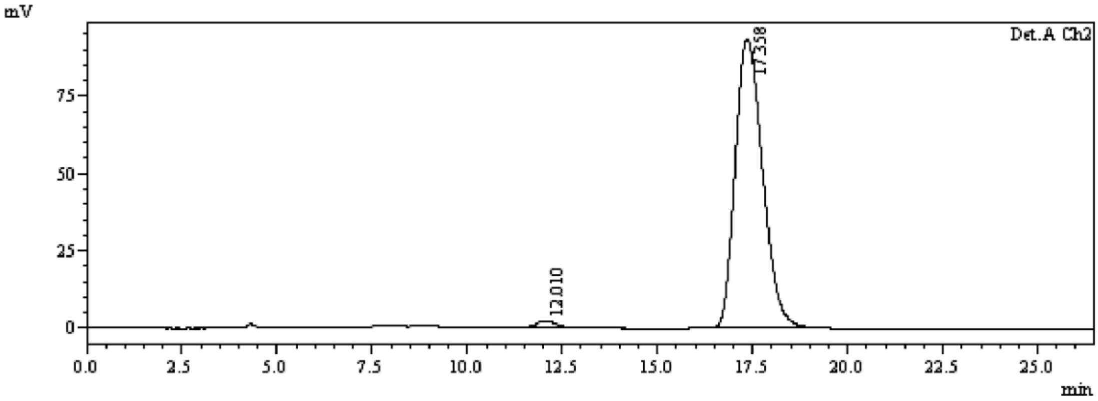

PeakTable

Detector A Ch2 254nm

Peak#	Ret. Time	Area	Height	Area %	Height %
1	12.010	61014	1919	1.297	2.022
2	17.358	4644479	92999	98.703	97.978
Total		4705493	94918	100.000	100.000

3. Mechanistic Investigations

3.1 Kinetic isotope effect (KIE) experiment

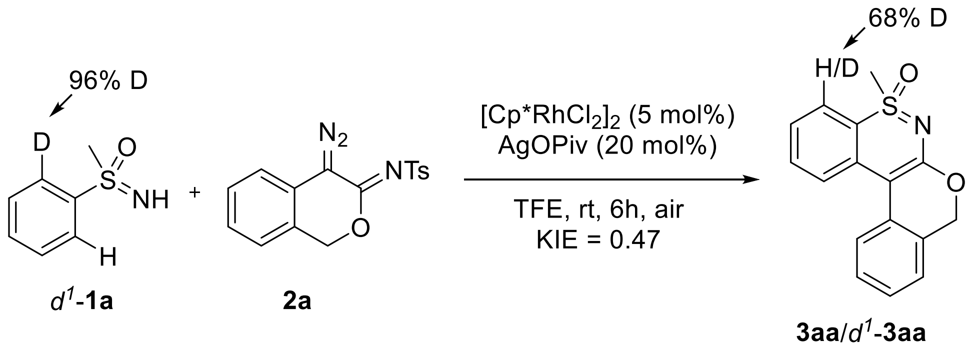

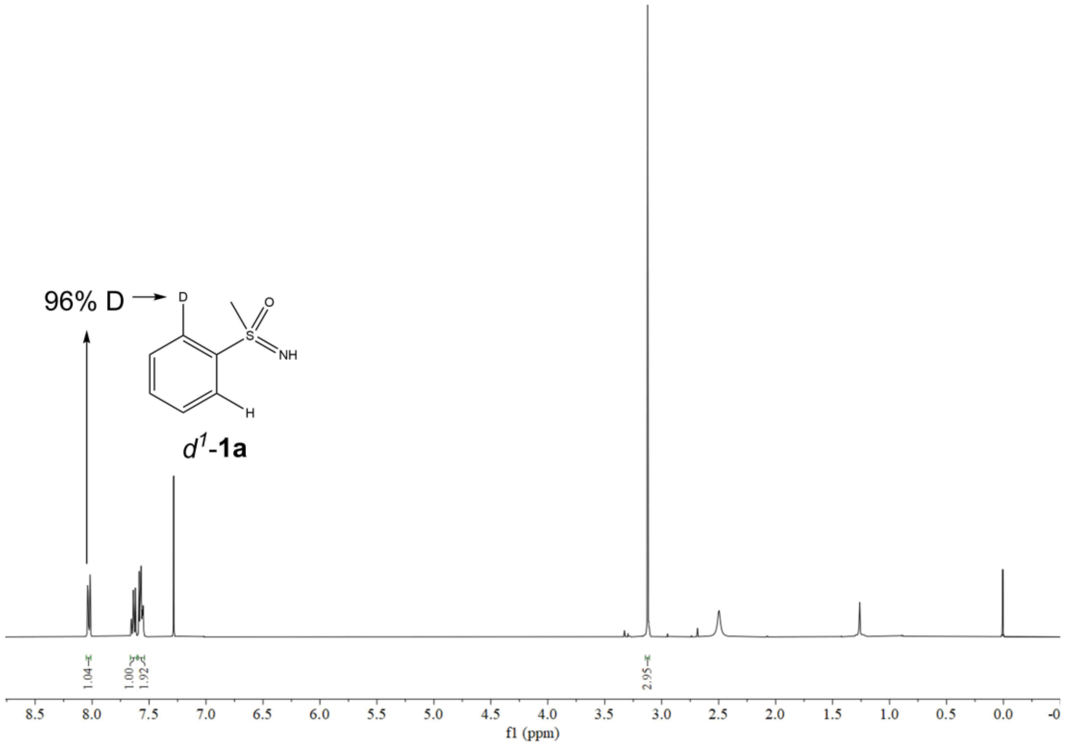

^{1}H NMR spectrum (400 MHz, $CDCl_3$) of d^1-**1a**

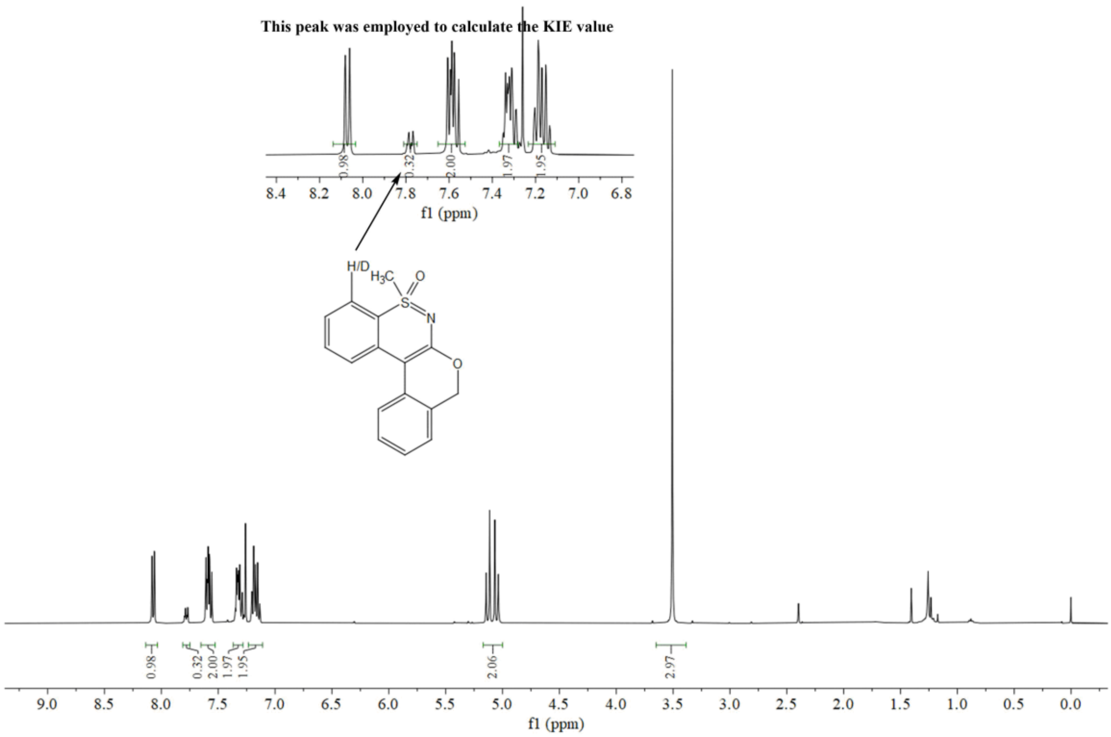

$k_H/k_D = 0.32/0.68 = 0.47$

3.2 H/D exchange experiment

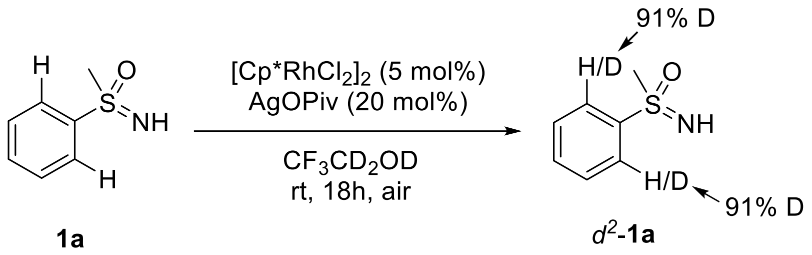

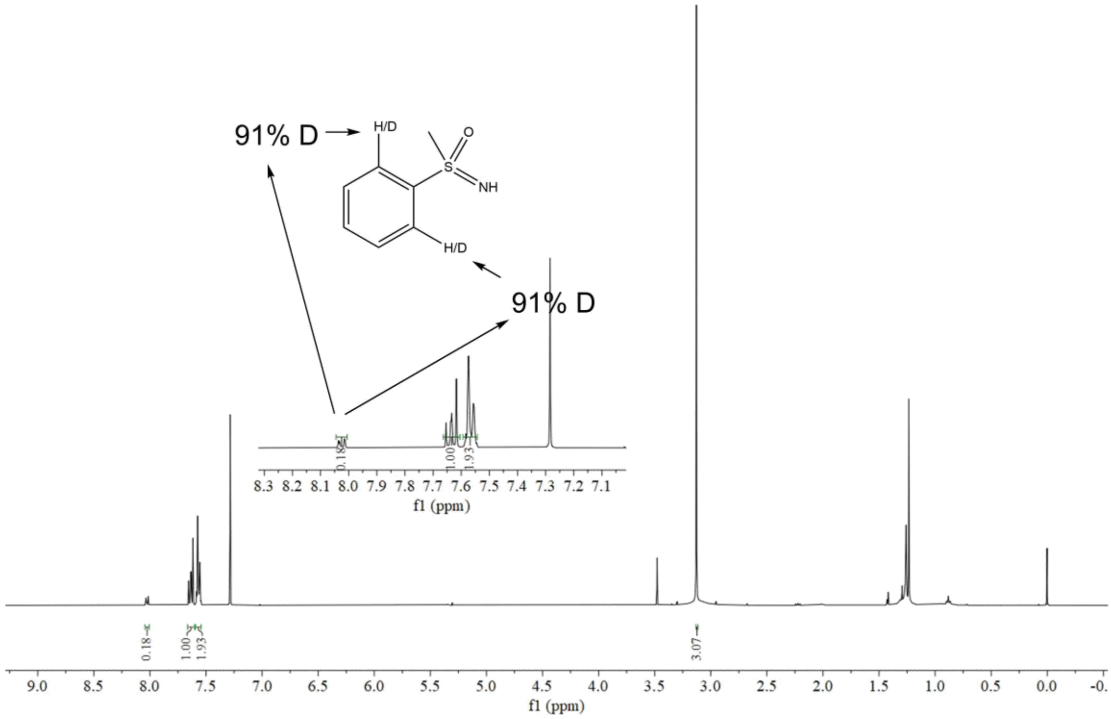

^{1}H NMR spectrum (400 MHz, $CDCl_3$) of d^2-**1a**

4. NMR Data

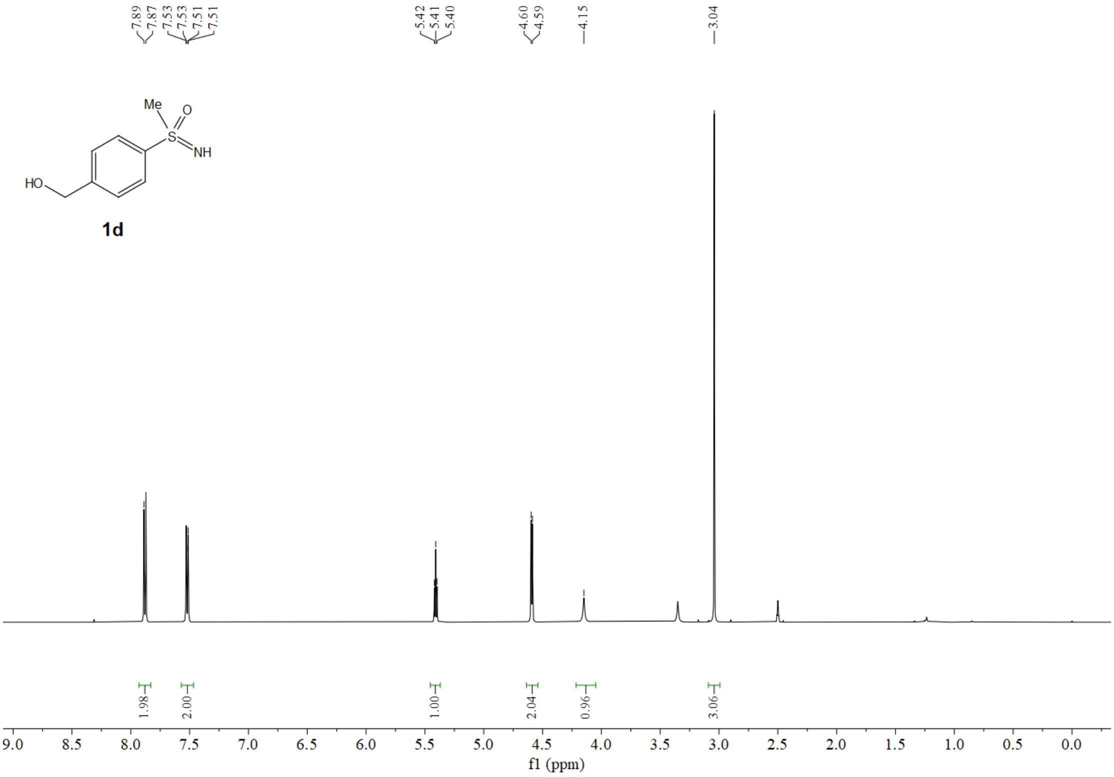

^{1}H NMR spectrum of **1d**

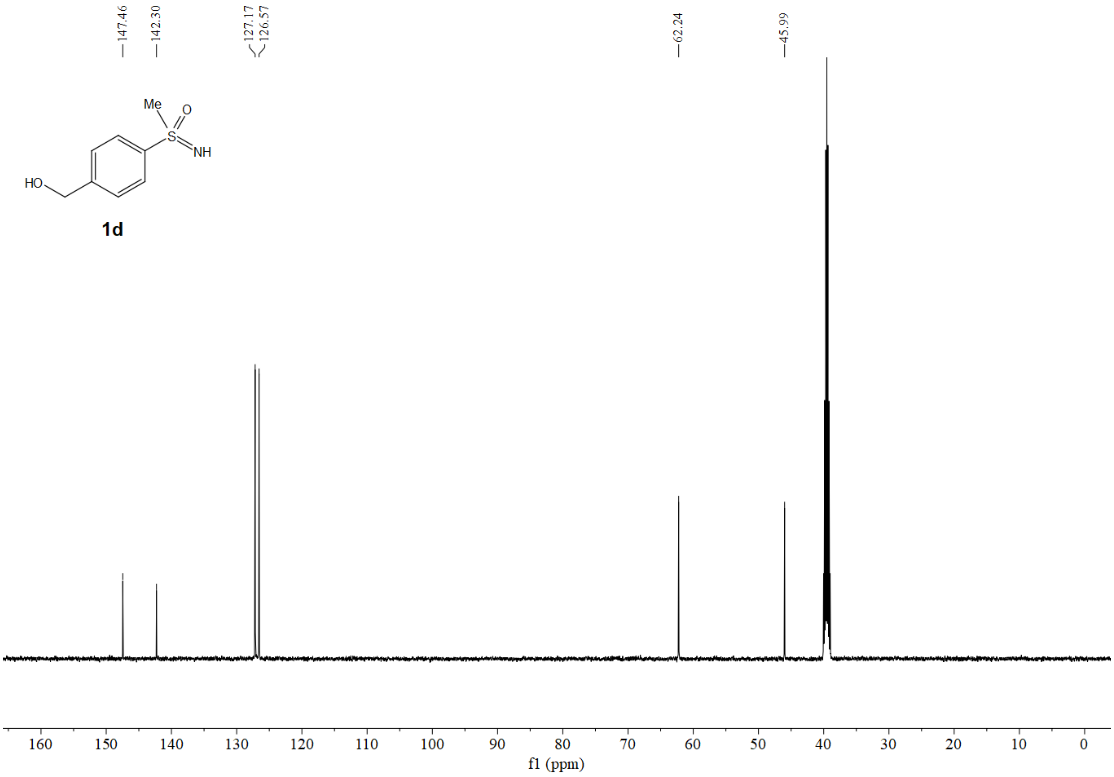

^{13}C NMR spectrum of **1d**

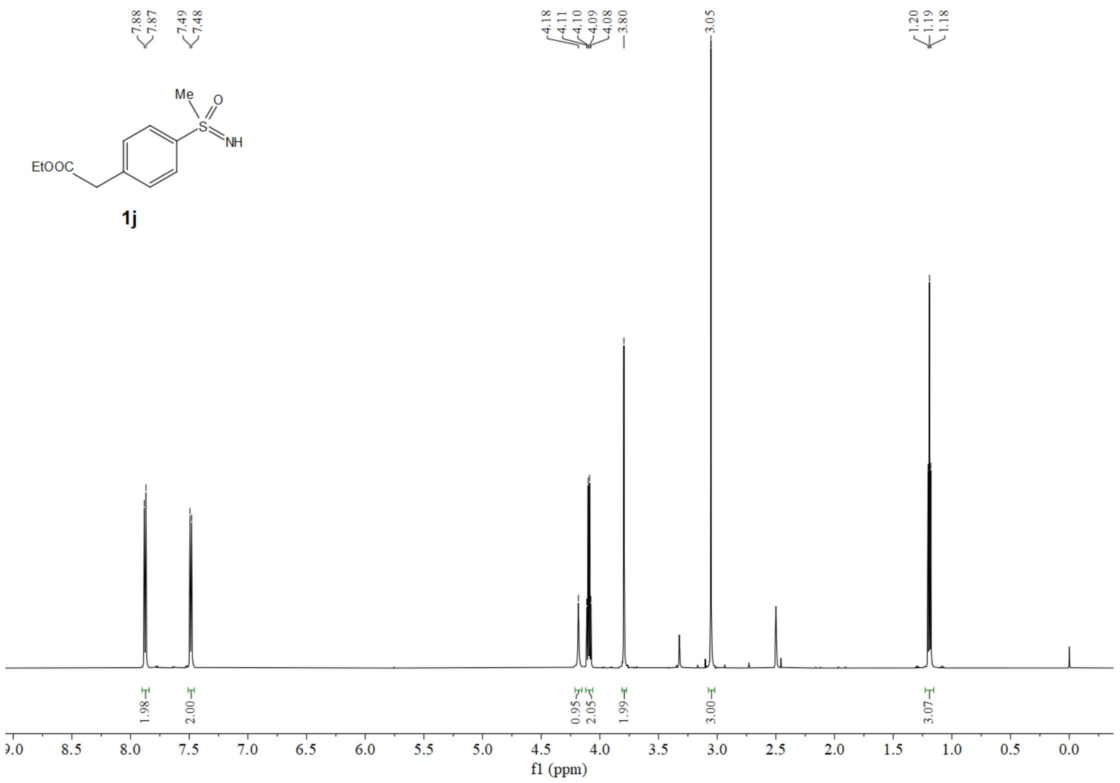

^{1}H NMR spectrum of **1j**

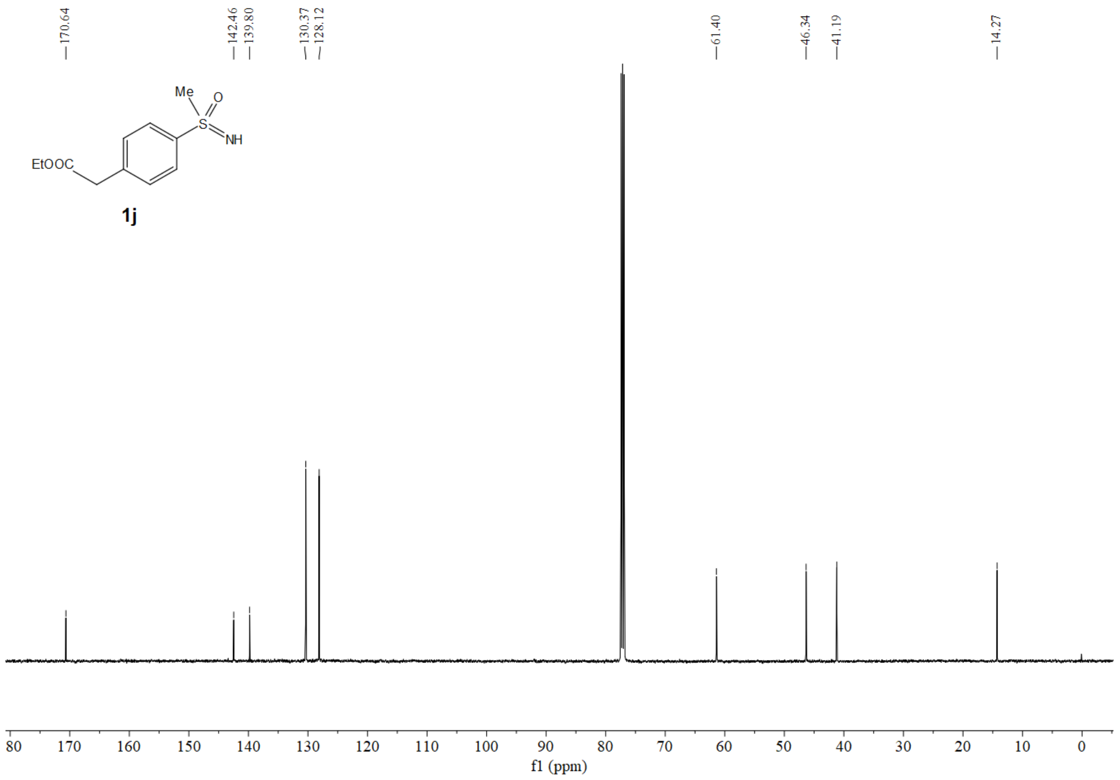

^{13}C NMR spectrum of **1j**

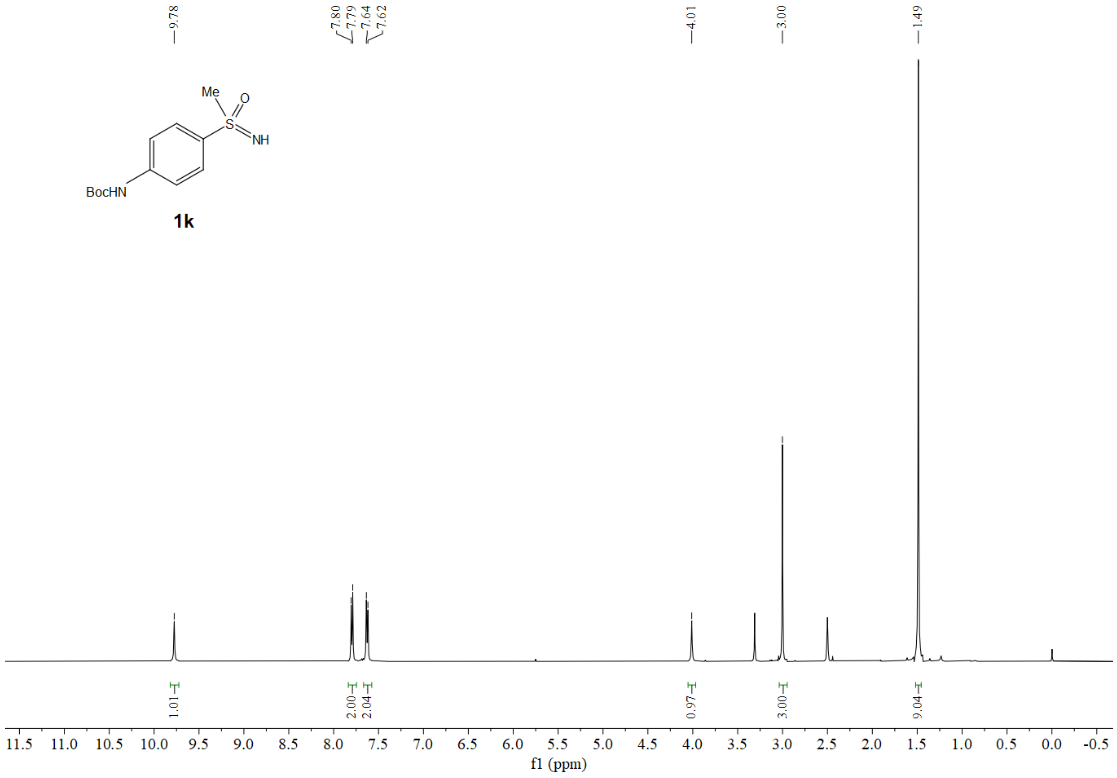

^{1}H NMR spectrum of **1k**

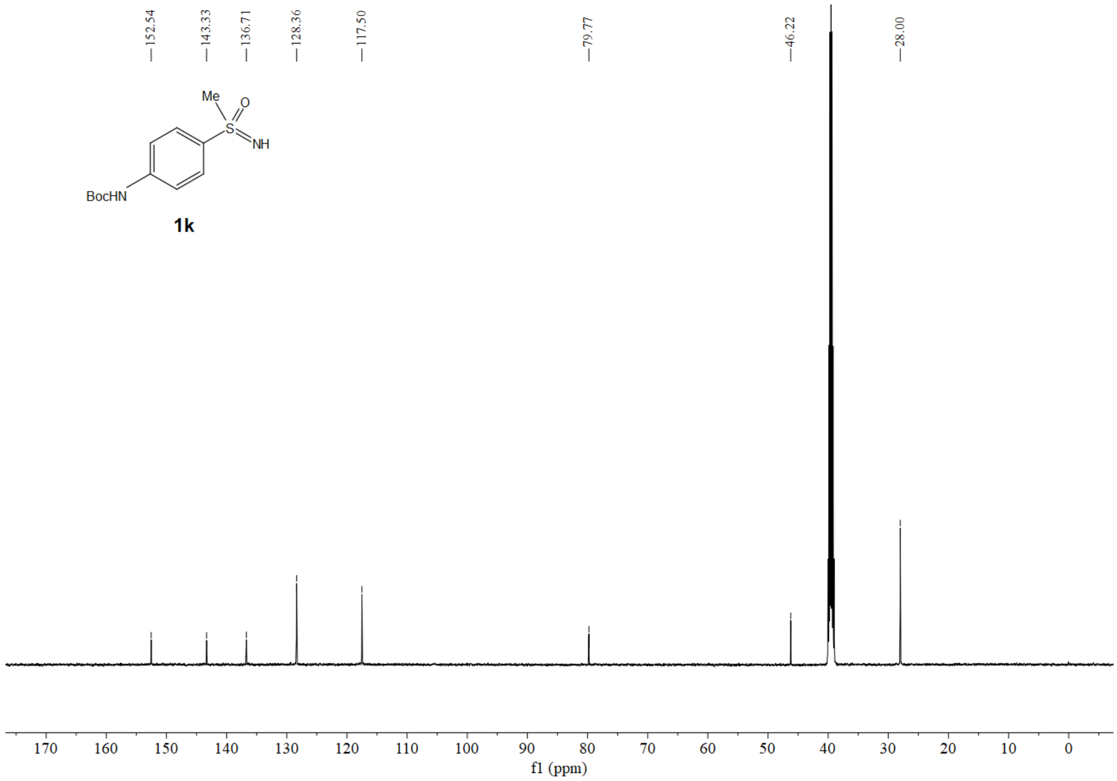

^{13}C NMR spectrum of **1k**

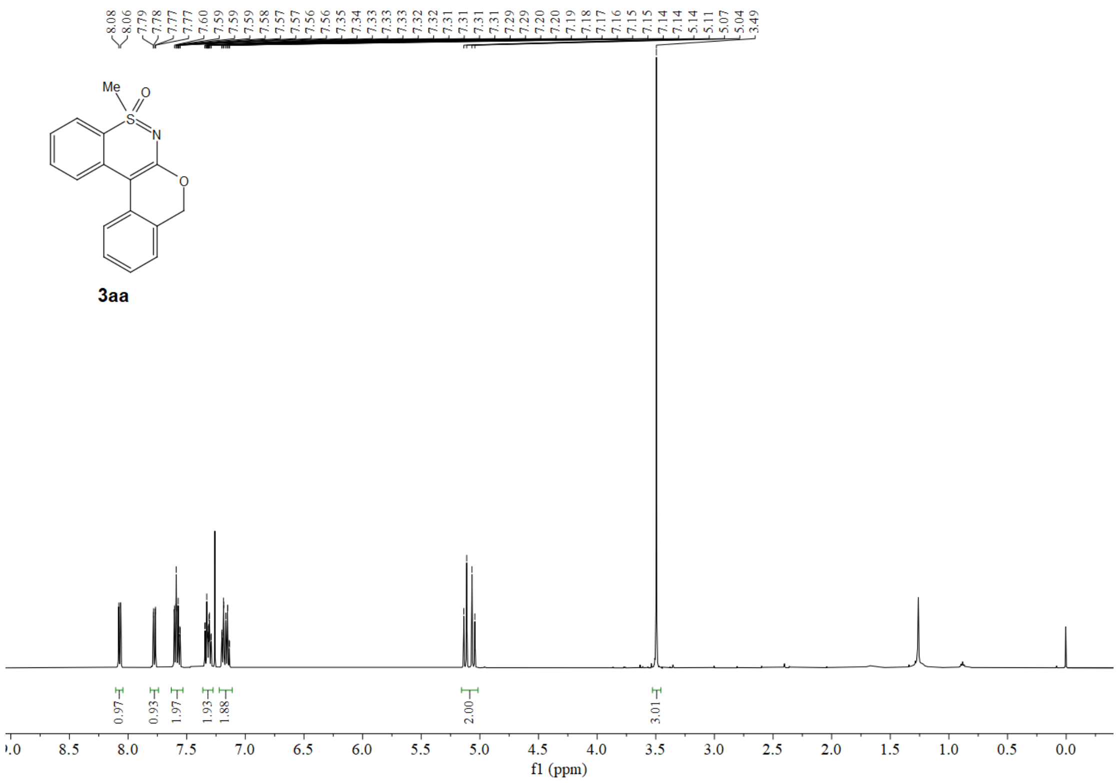

^{1}H NMR spectrum of **3aa**

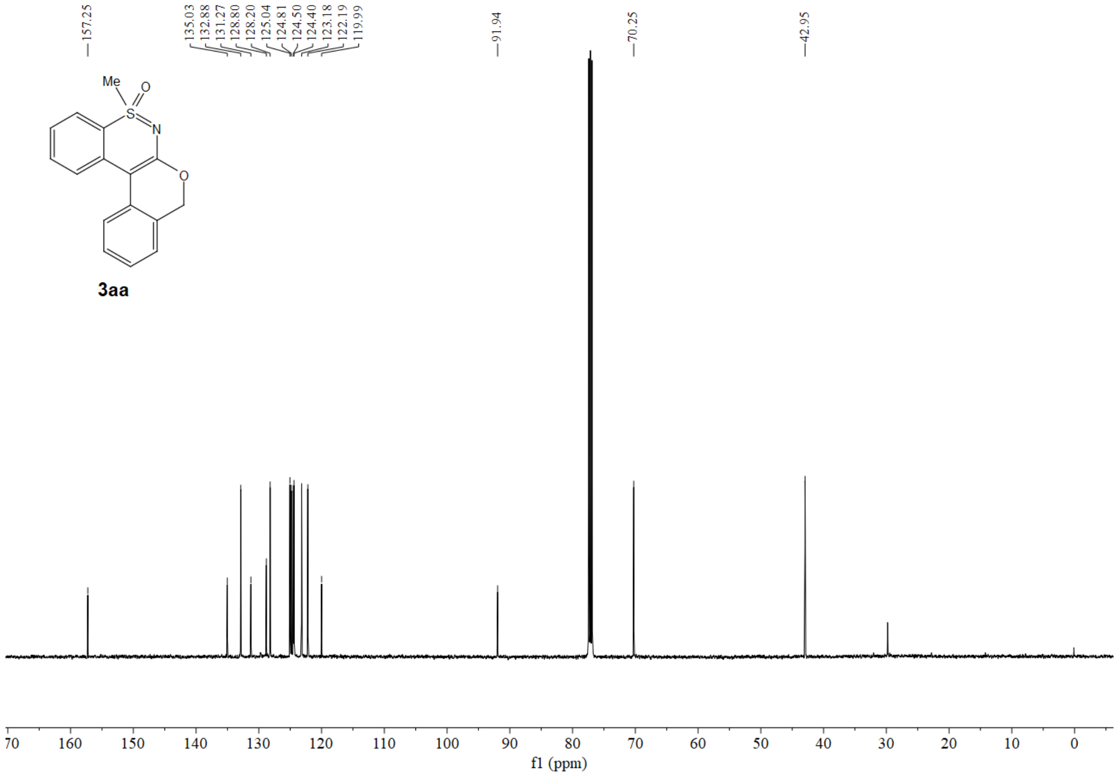

^{13}C NMR spectrum of **3aa**

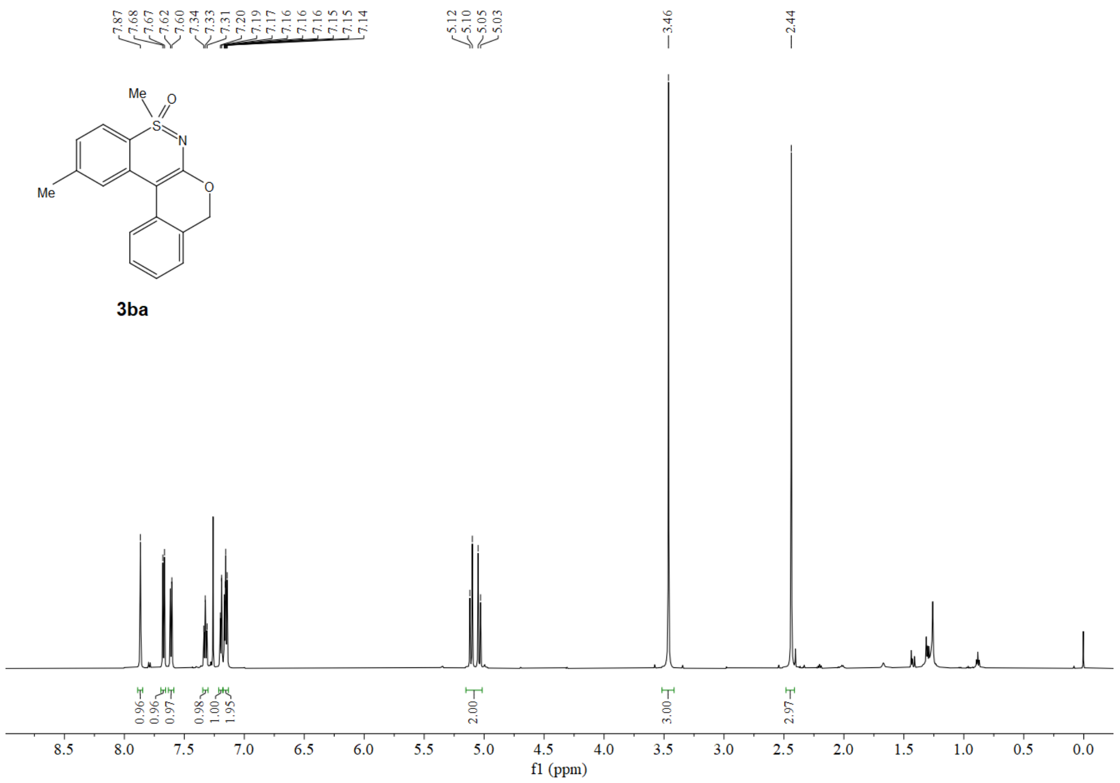

^{1}H NMR spectrum of **3ba**

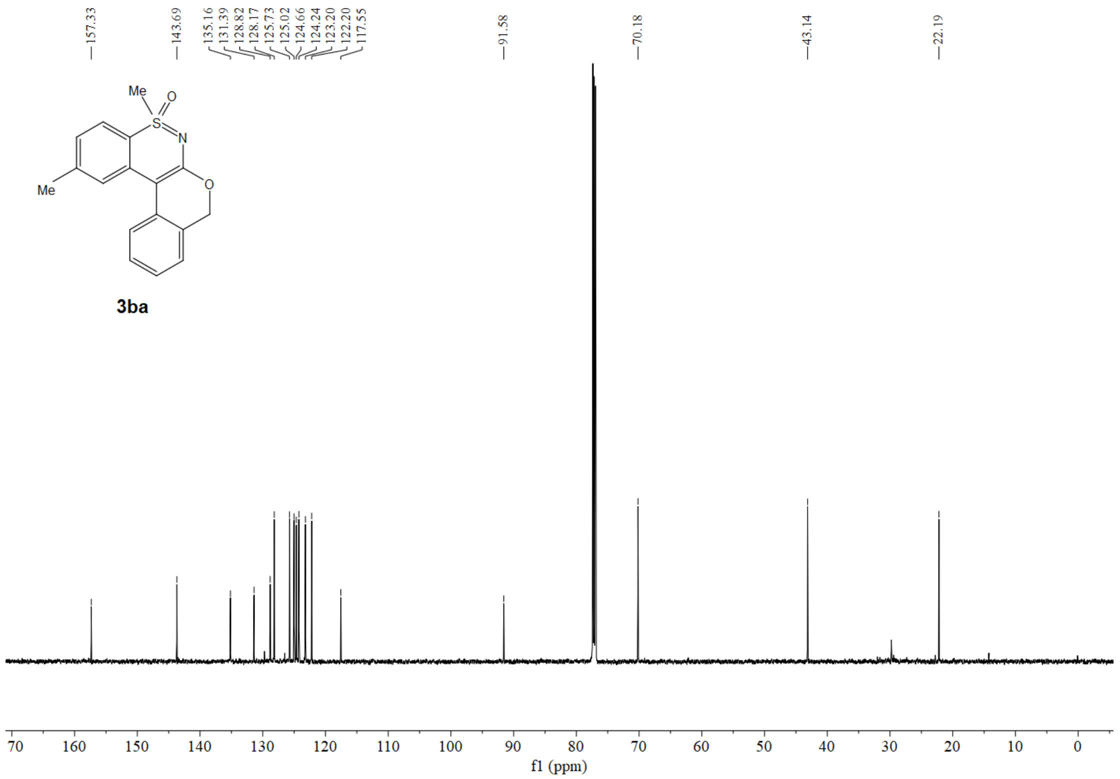

^{13}C NMR spectrum of **3ba**

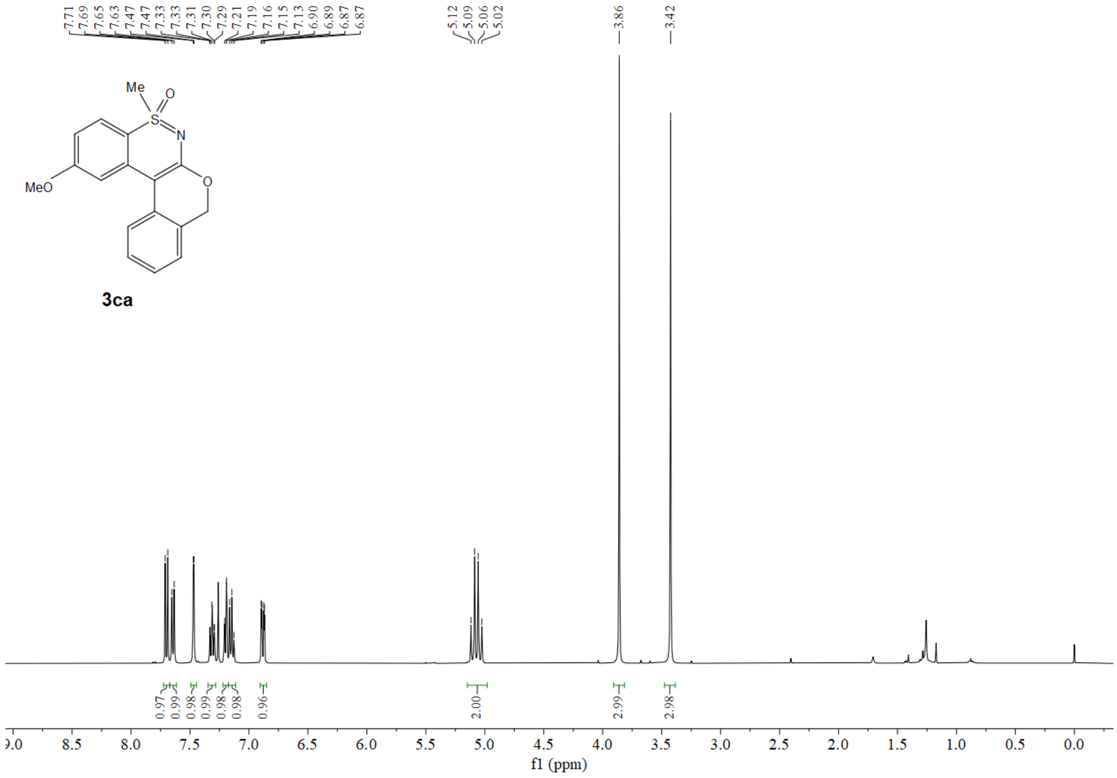

^{1}H NMR spectrum of **3ca**

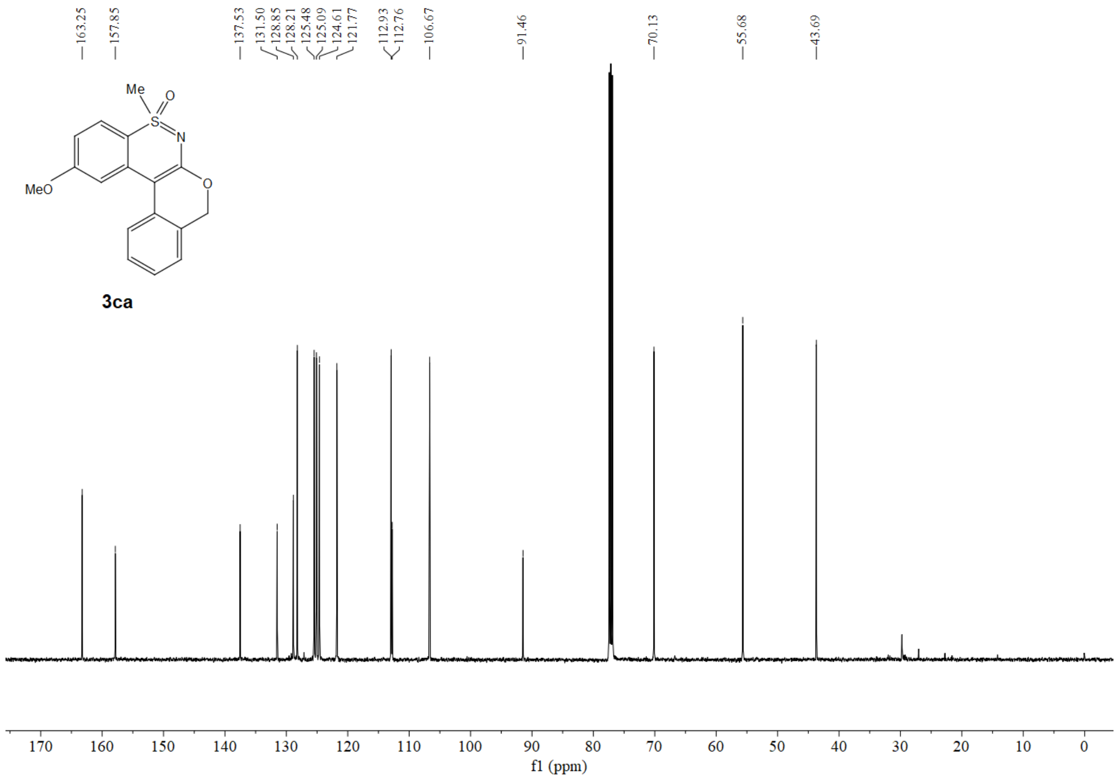

^{13}C NMR spectrum of **3ca**

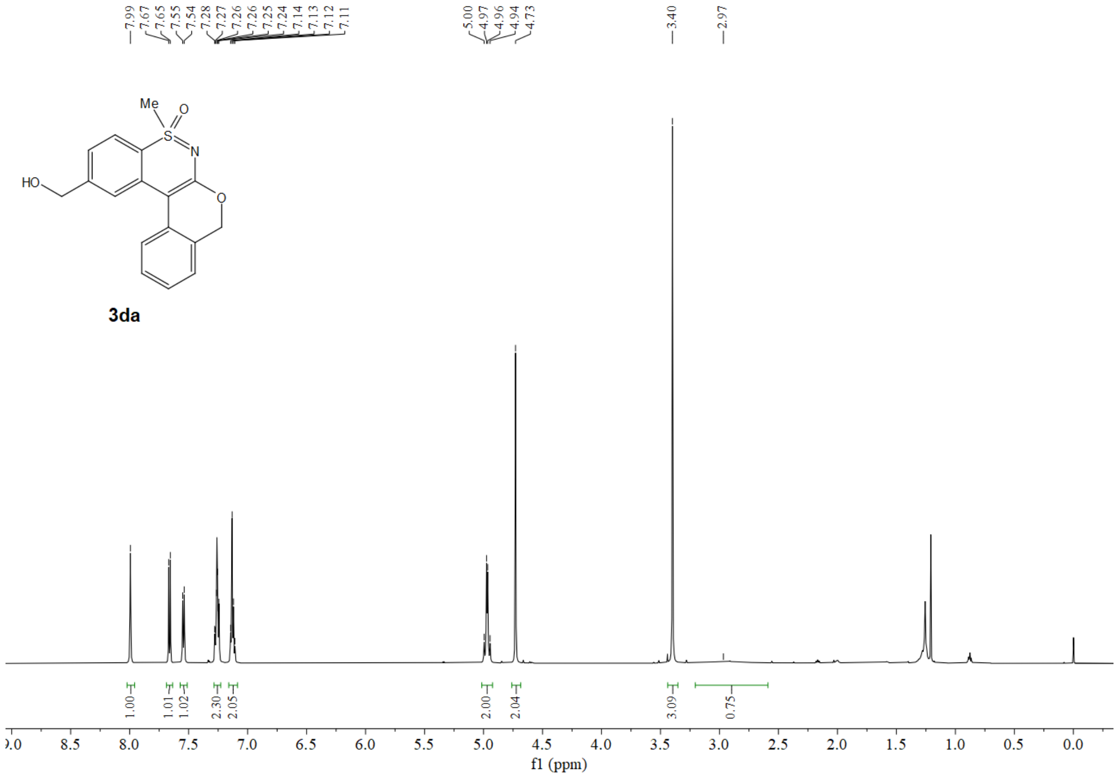

^{1}H NMR spectrum of **3da**

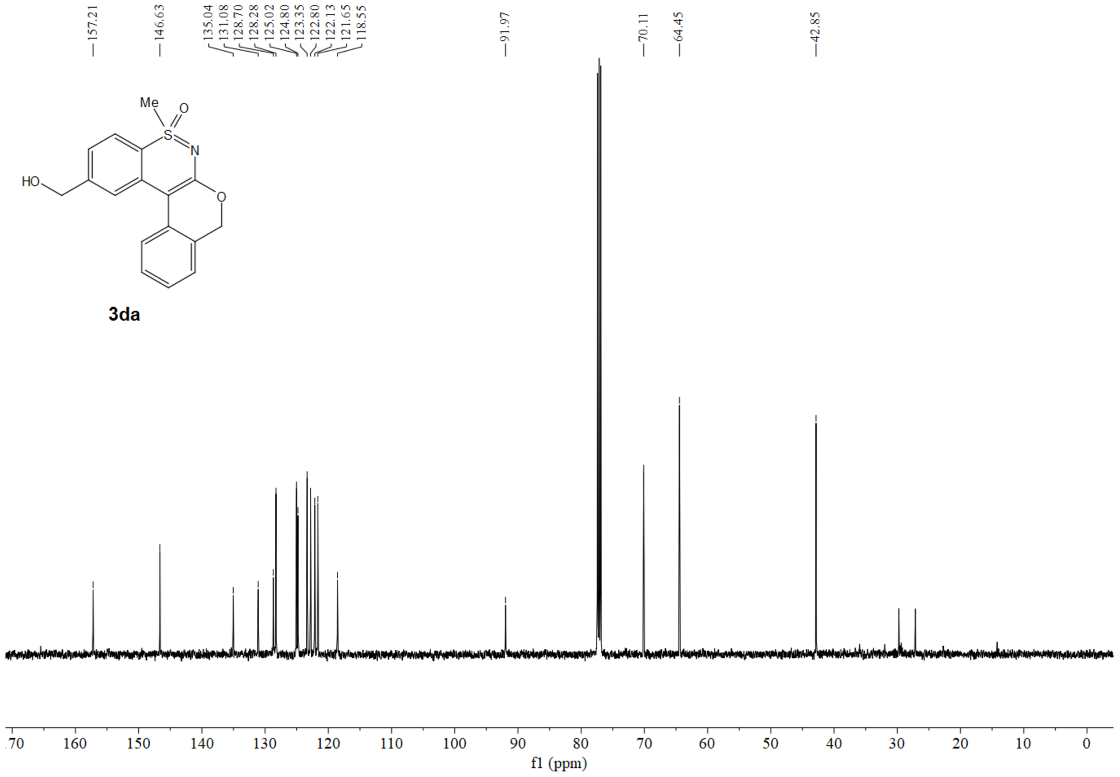

^{13}C NMR spectrum of **3da**

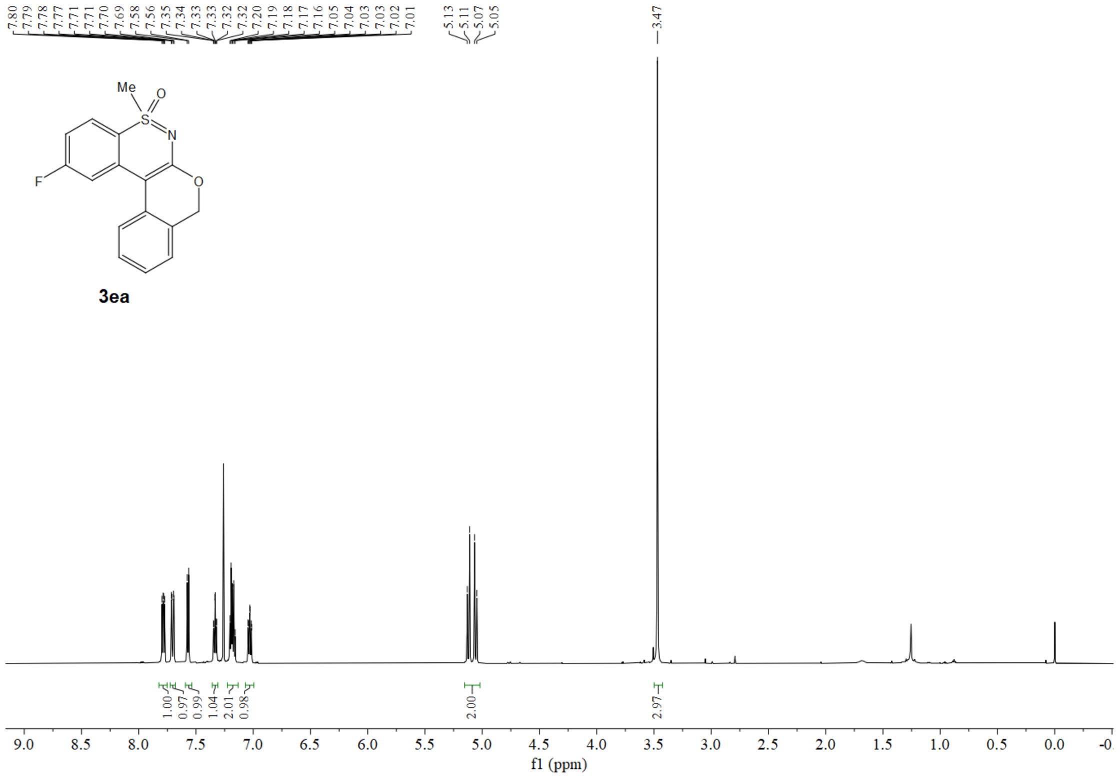

^{1}H NMR spectrum of **3ea**

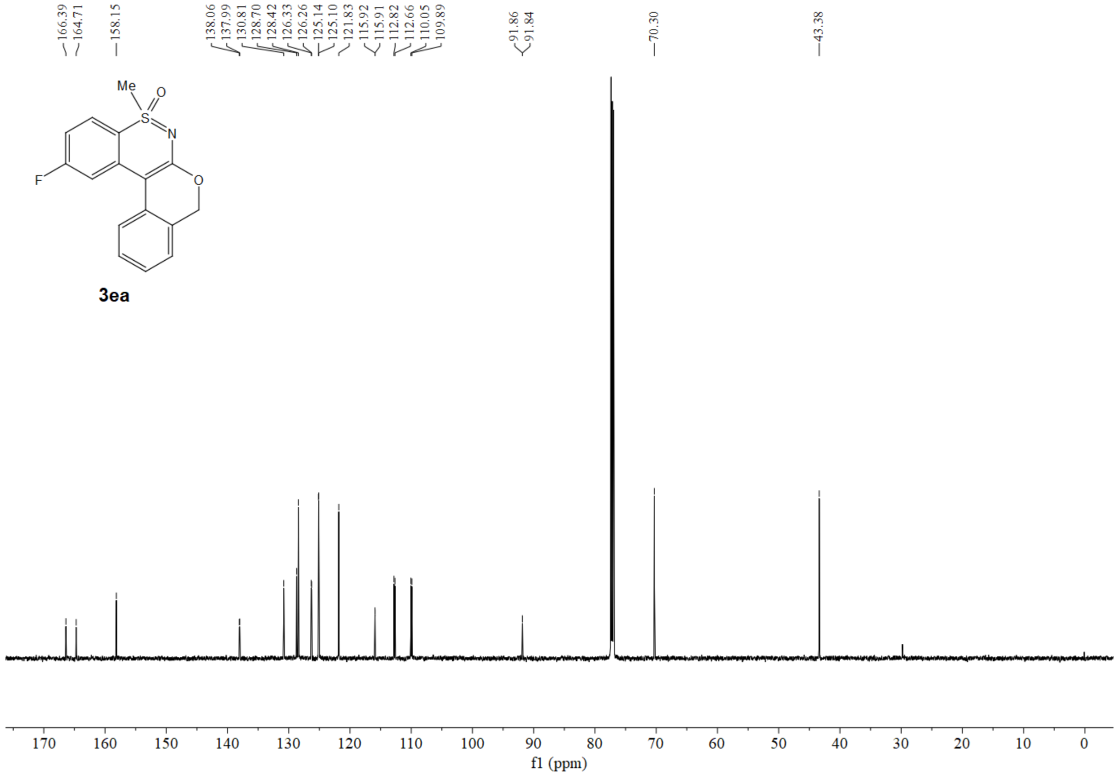

^{13}C NMR spectrum of **3ea**

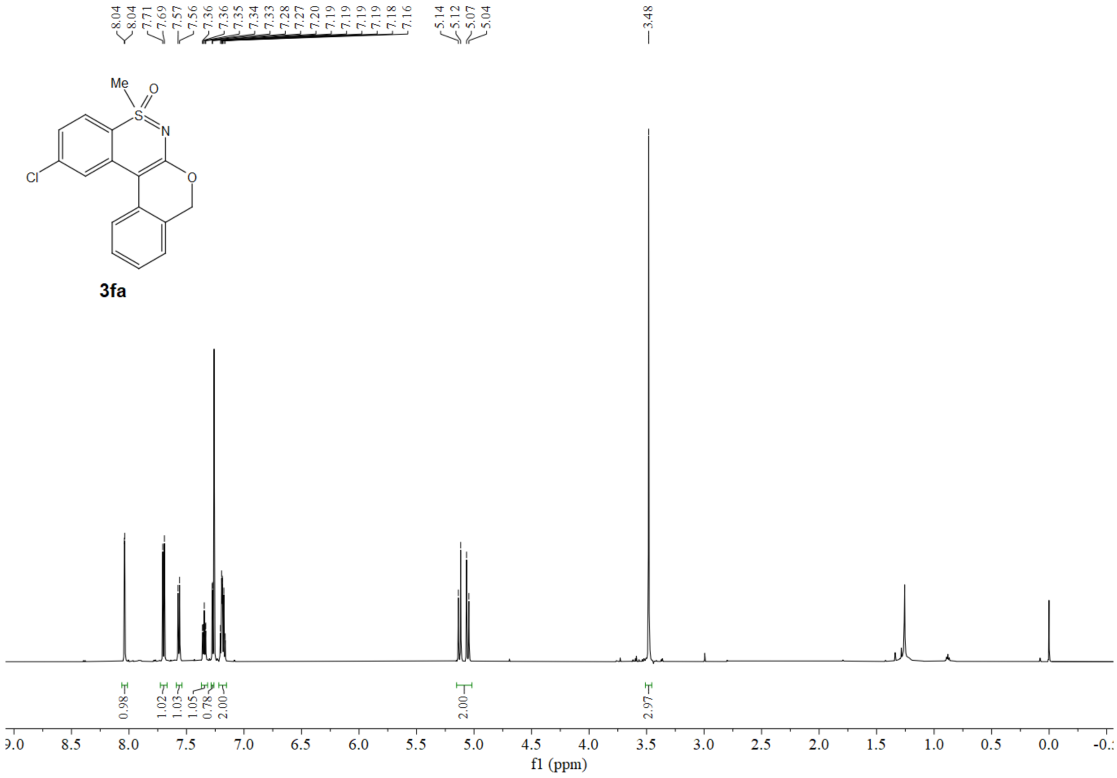

^{1}H NMR spectrum of **3fa**

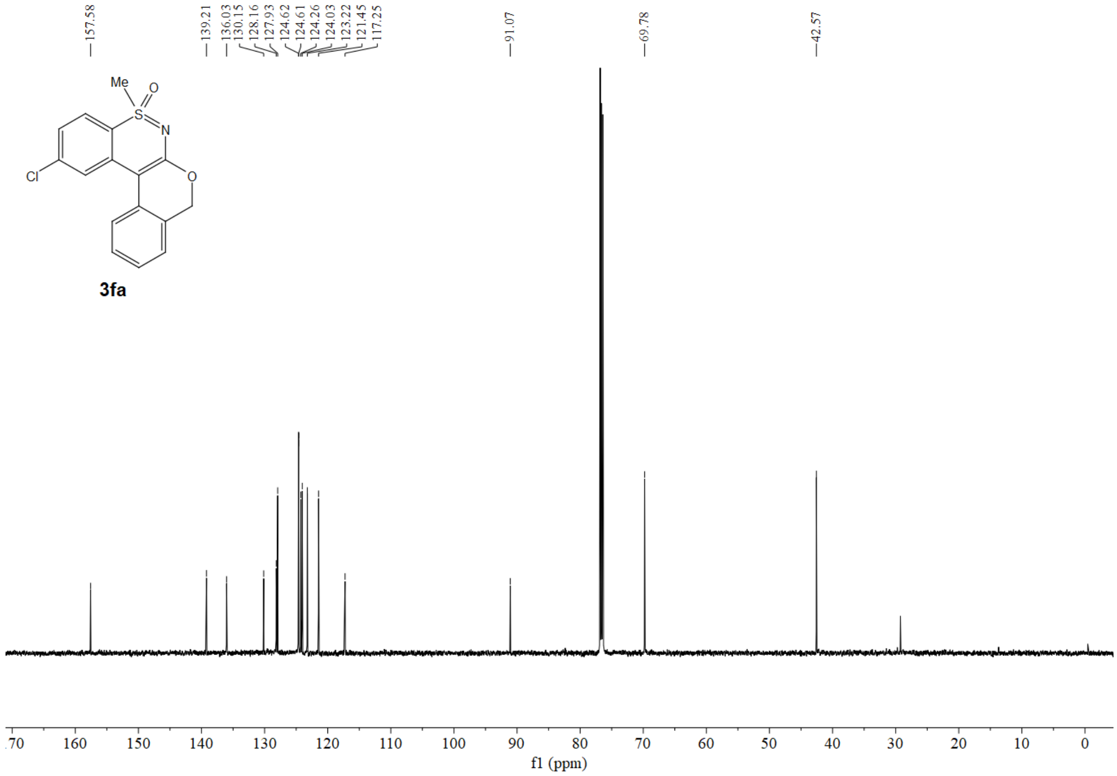

^{13}C NMR spectrum of **3fa**

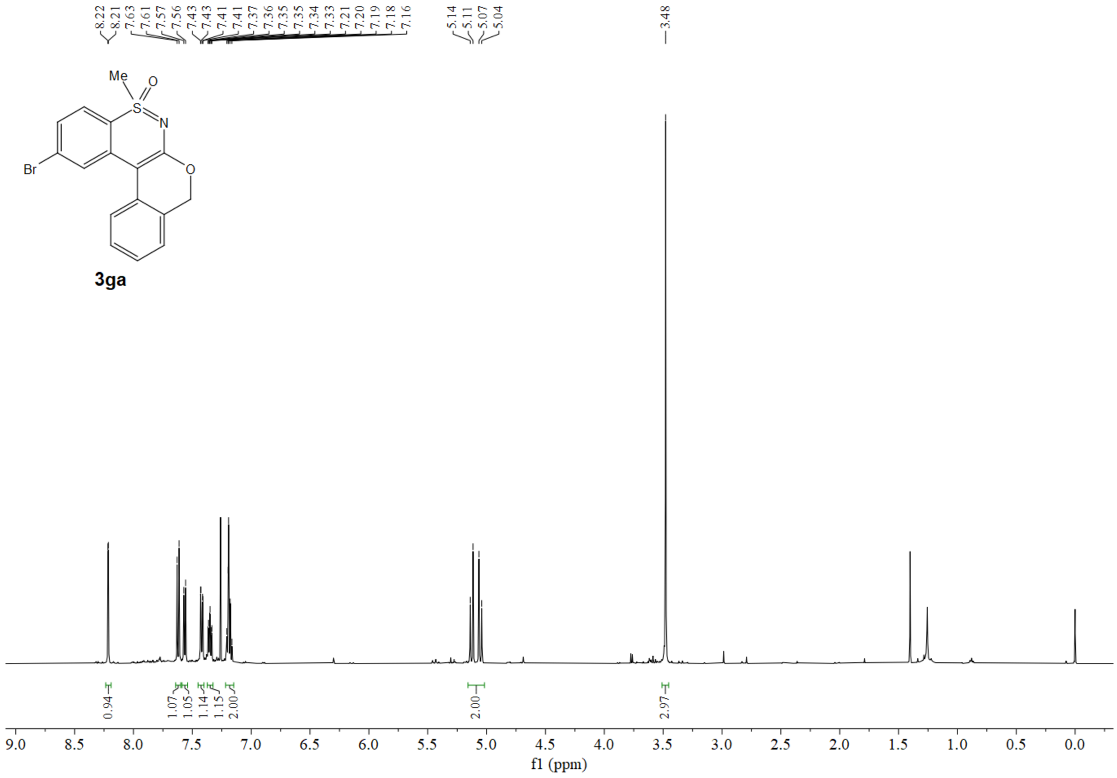

^{1}H NMR spectrum of **3ga**

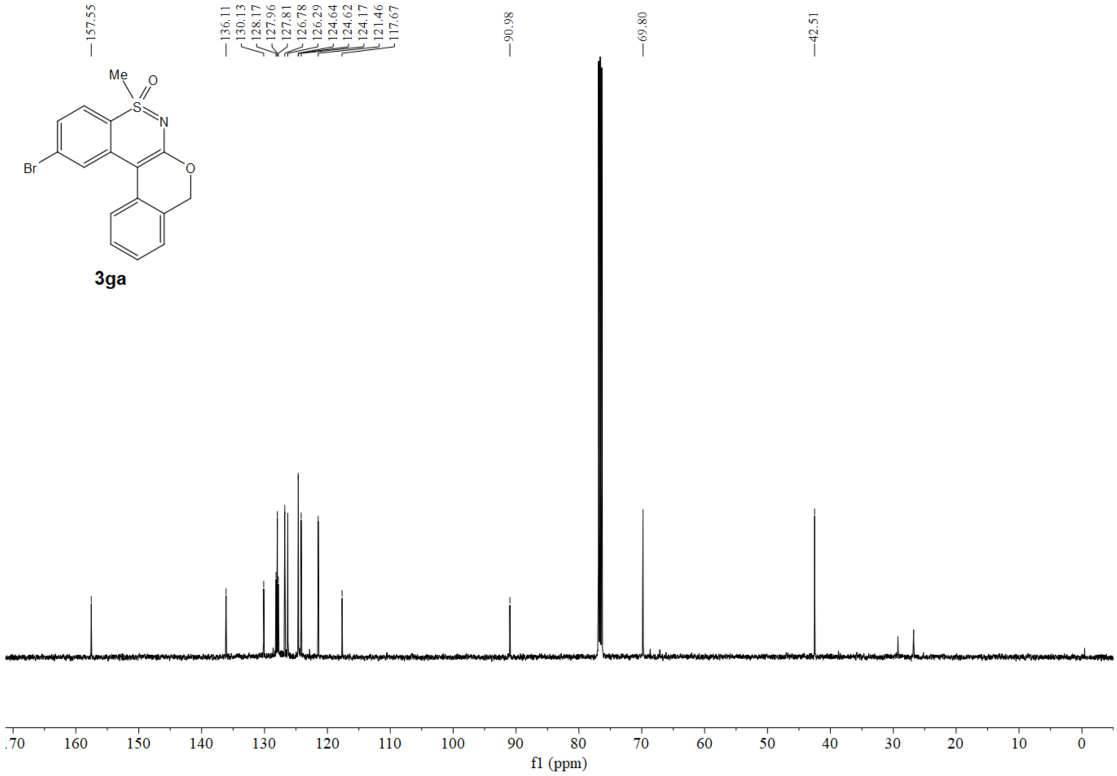

^{13}C NMR spectrum of **3ga**

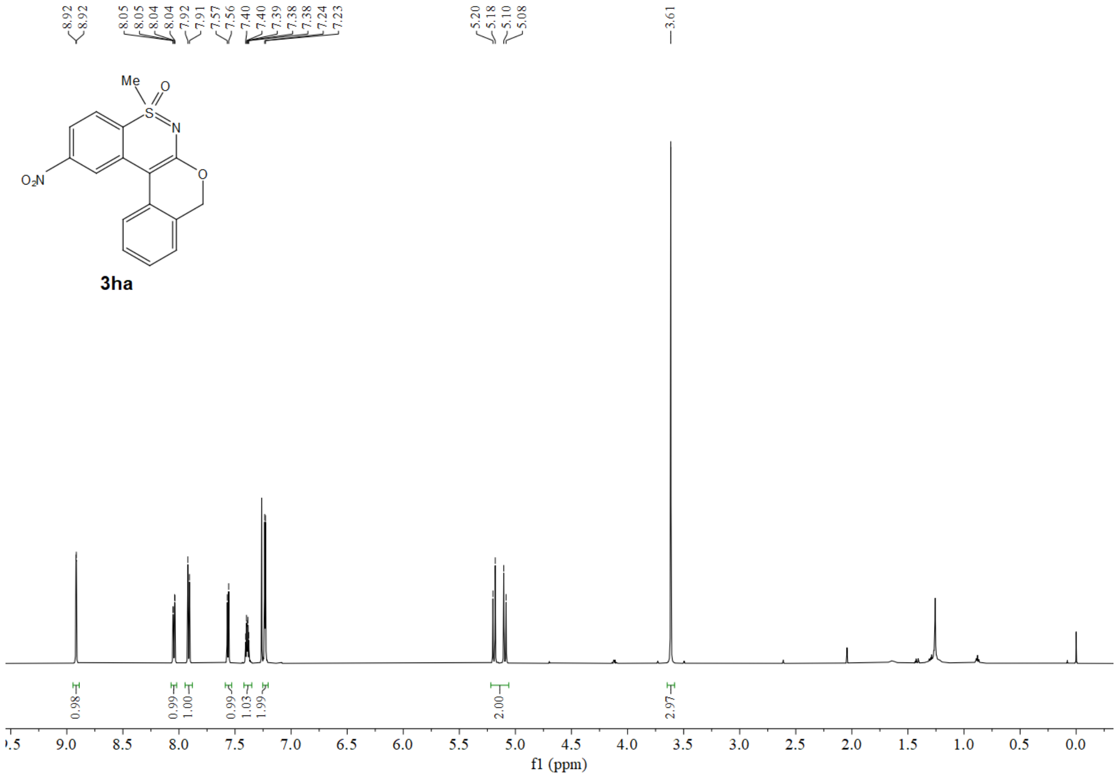

^{1}H NMR spectrum of **3ha**

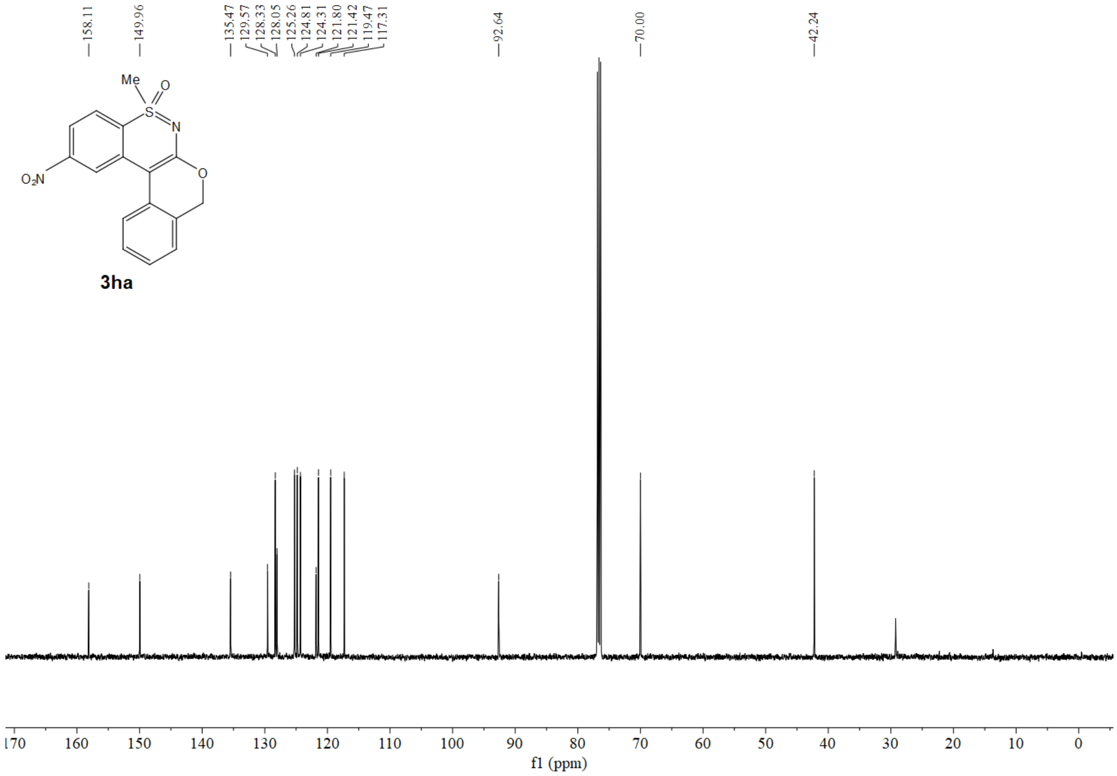

^{13}C NMR spectrum of **3ha**

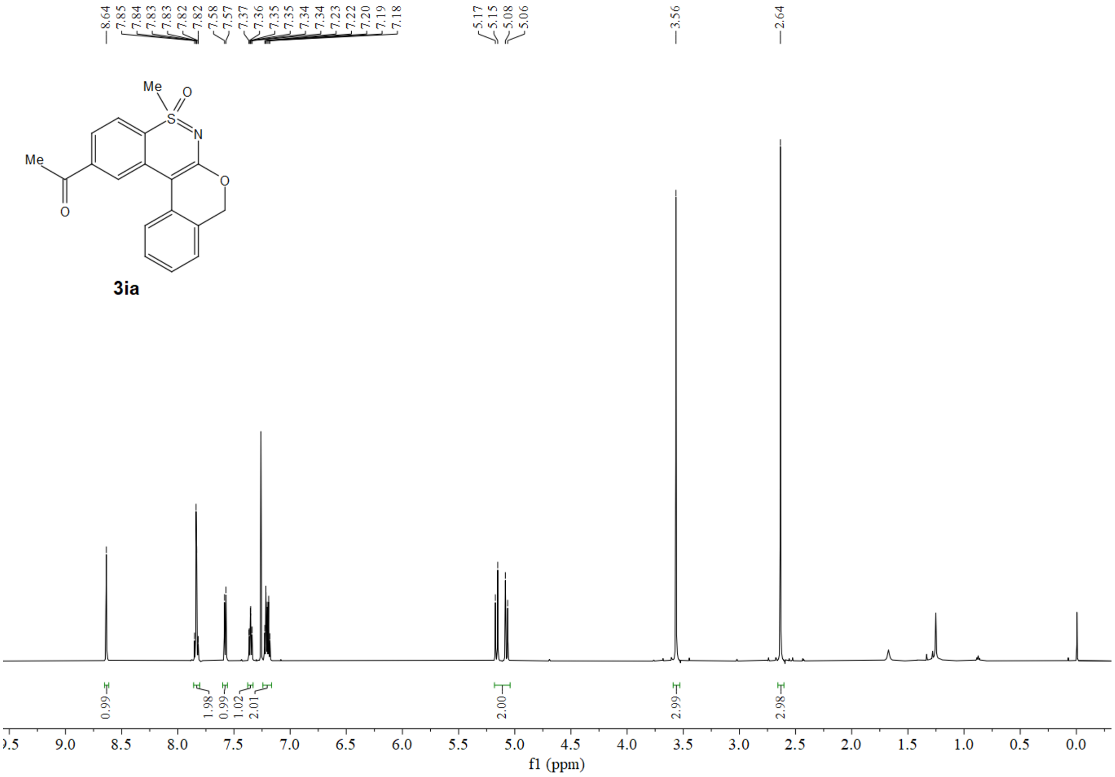

^{1}H NMR spectrum of **3ia**

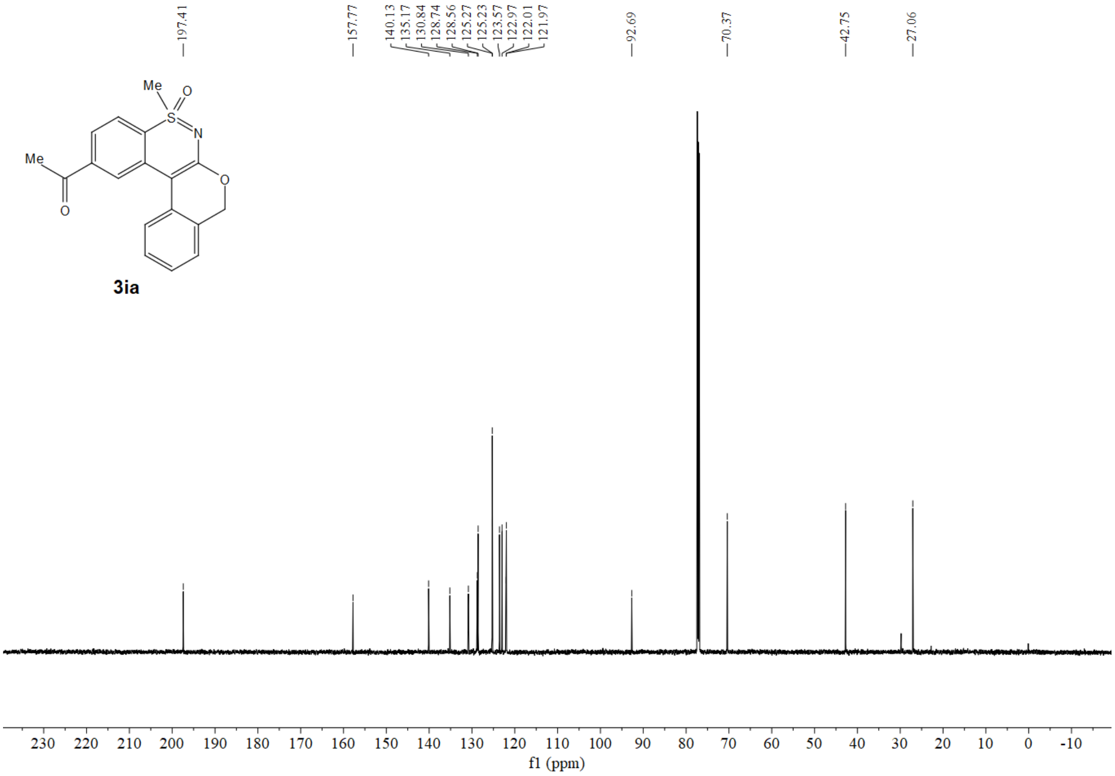

^{13}C NMR spectrum of **3ia**

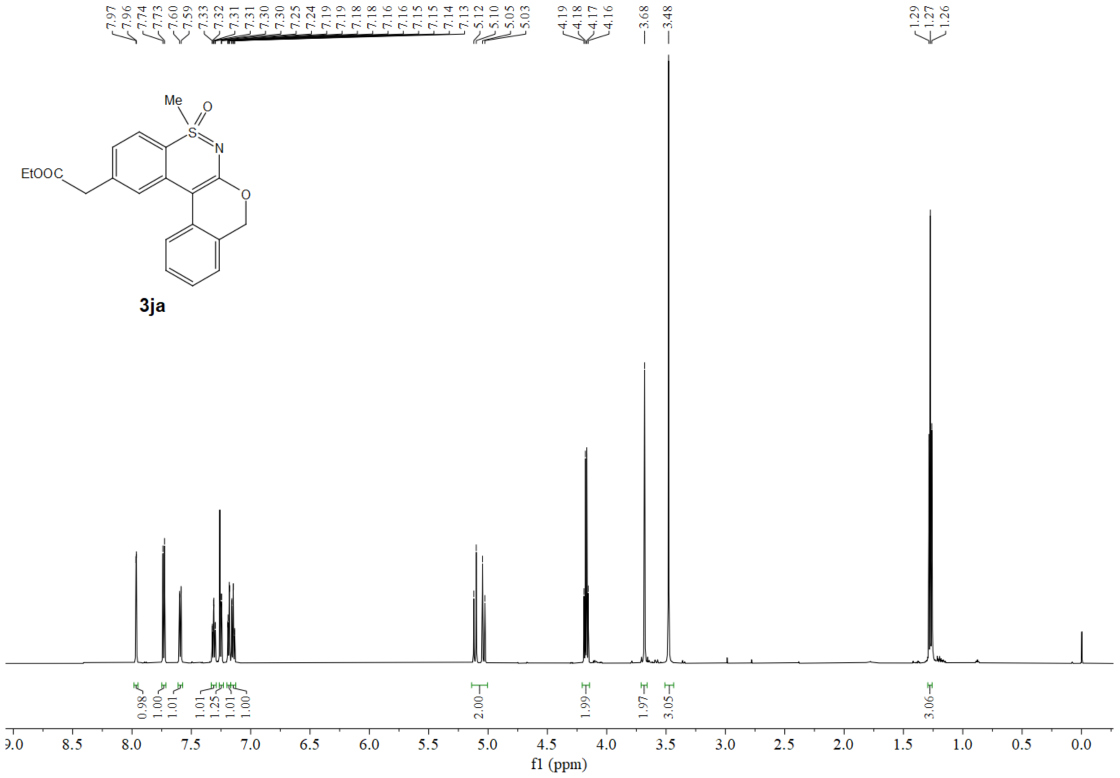

^{1}H NMR spectrum of **3ja**

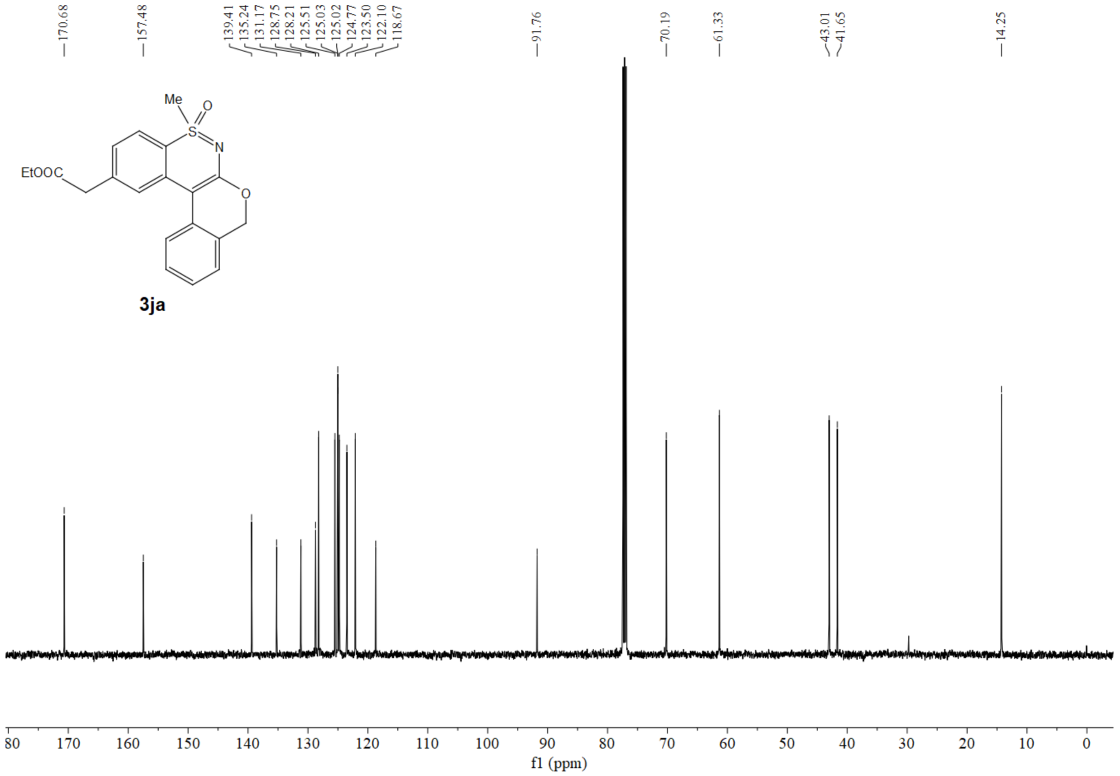

^{13}C NMR spectrum of **3ja**

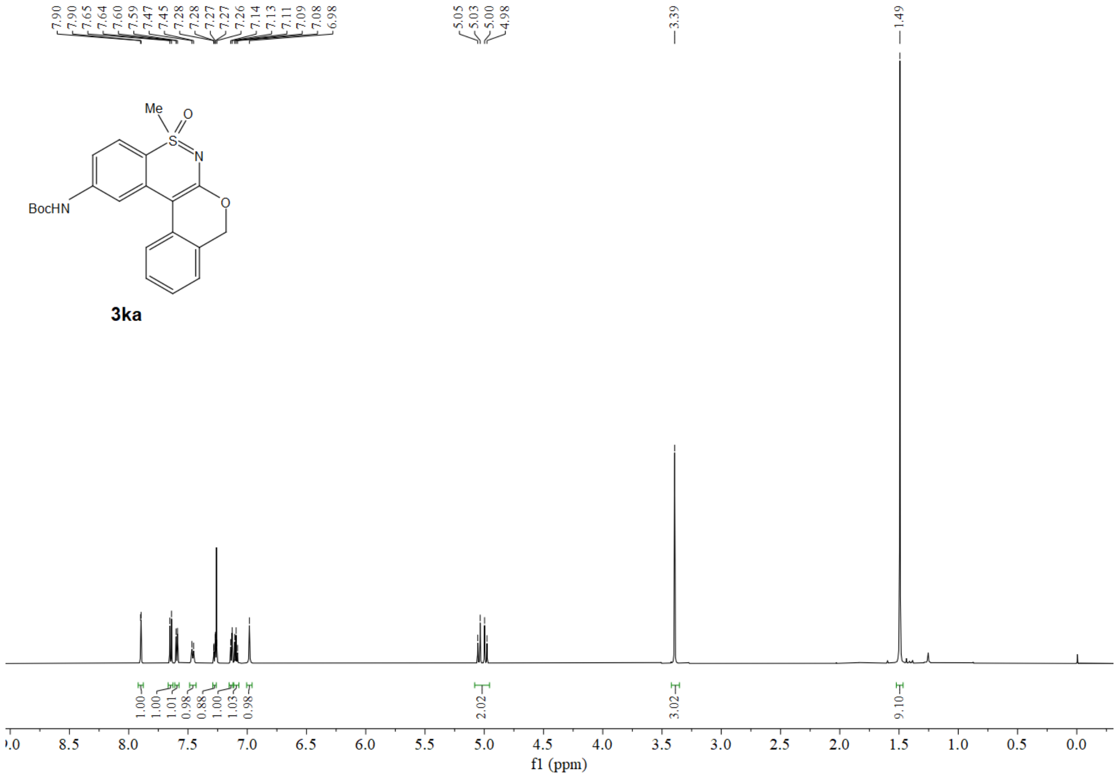

^{1}H NMR spectrum of **3ka**

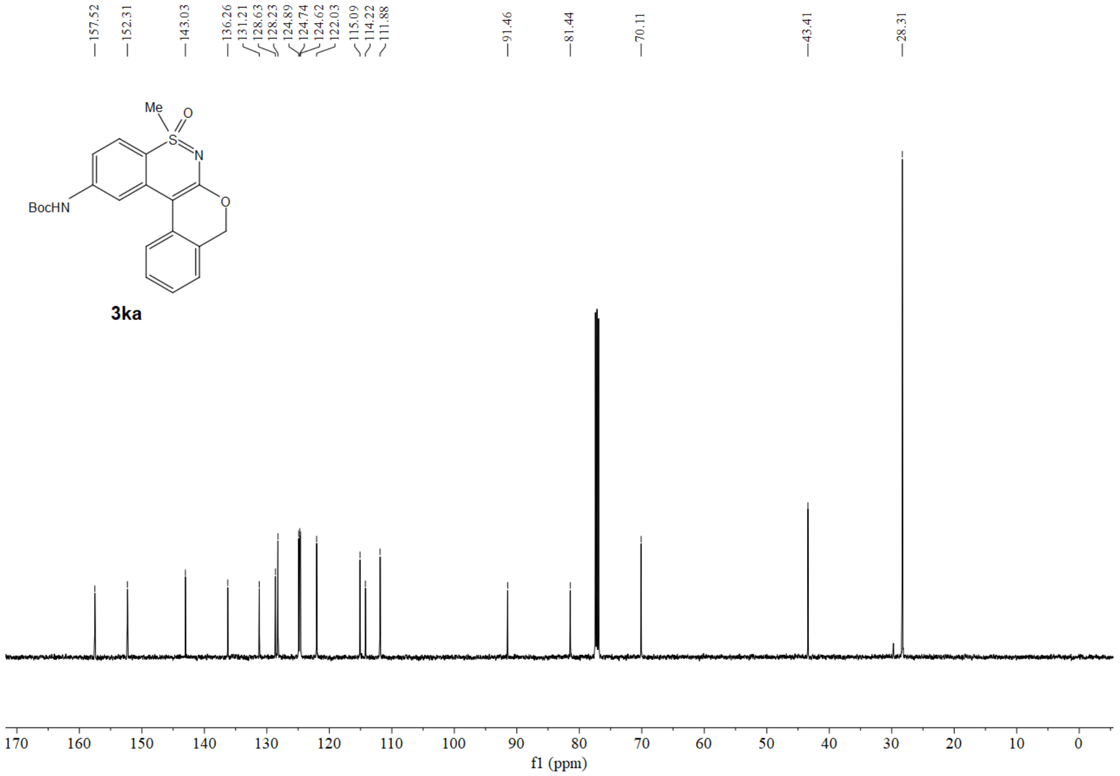

^{13}C NMR spectrum of **3ka**

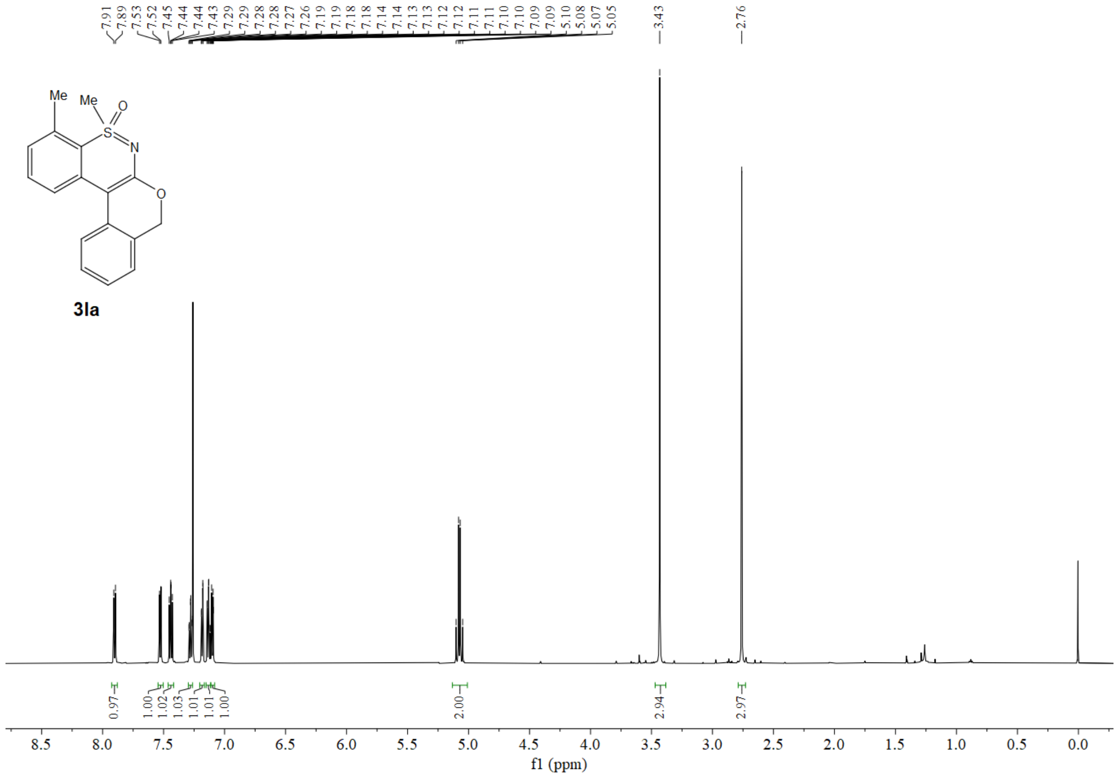

^{1}H NMR spectrum of **3la**

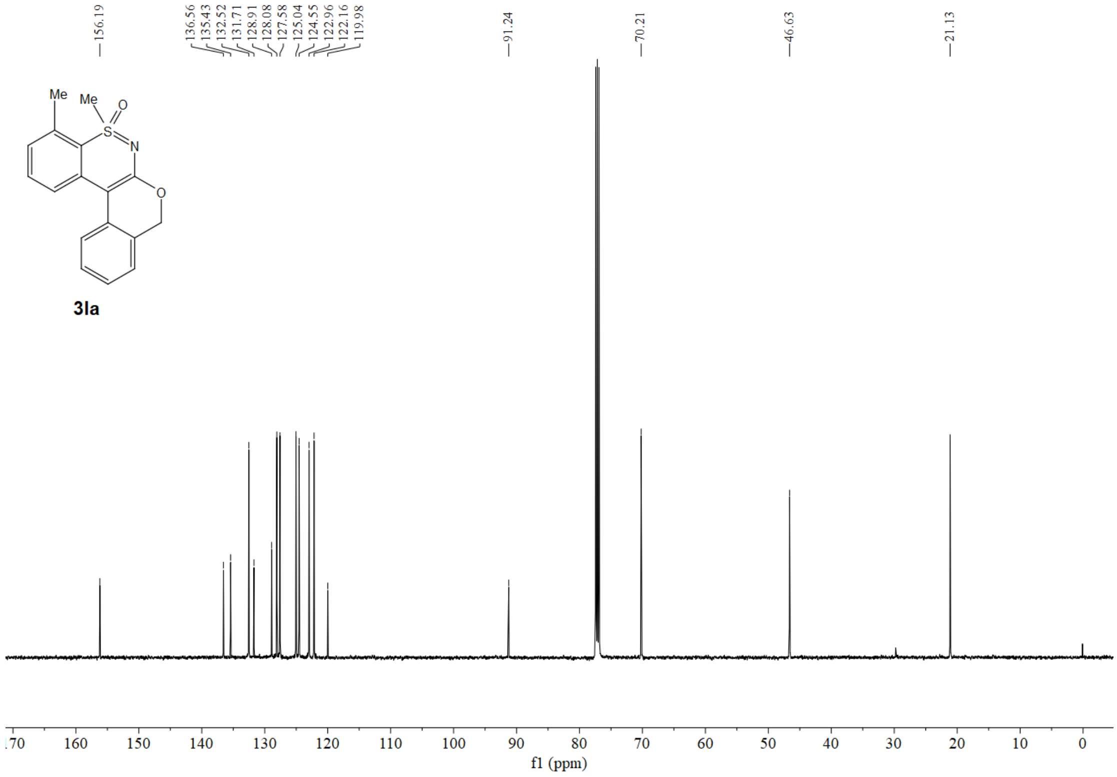

^{13}C NMR spectrum of **3la**

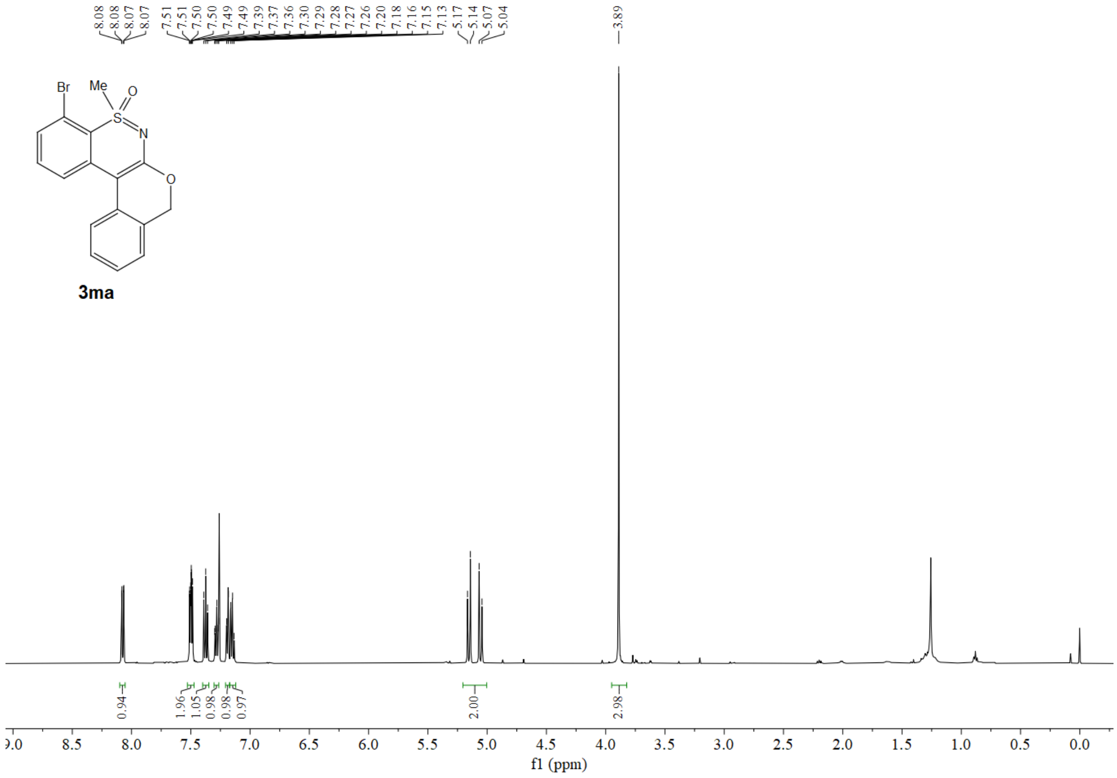

^{1}H NMR spectrum of **3ma**

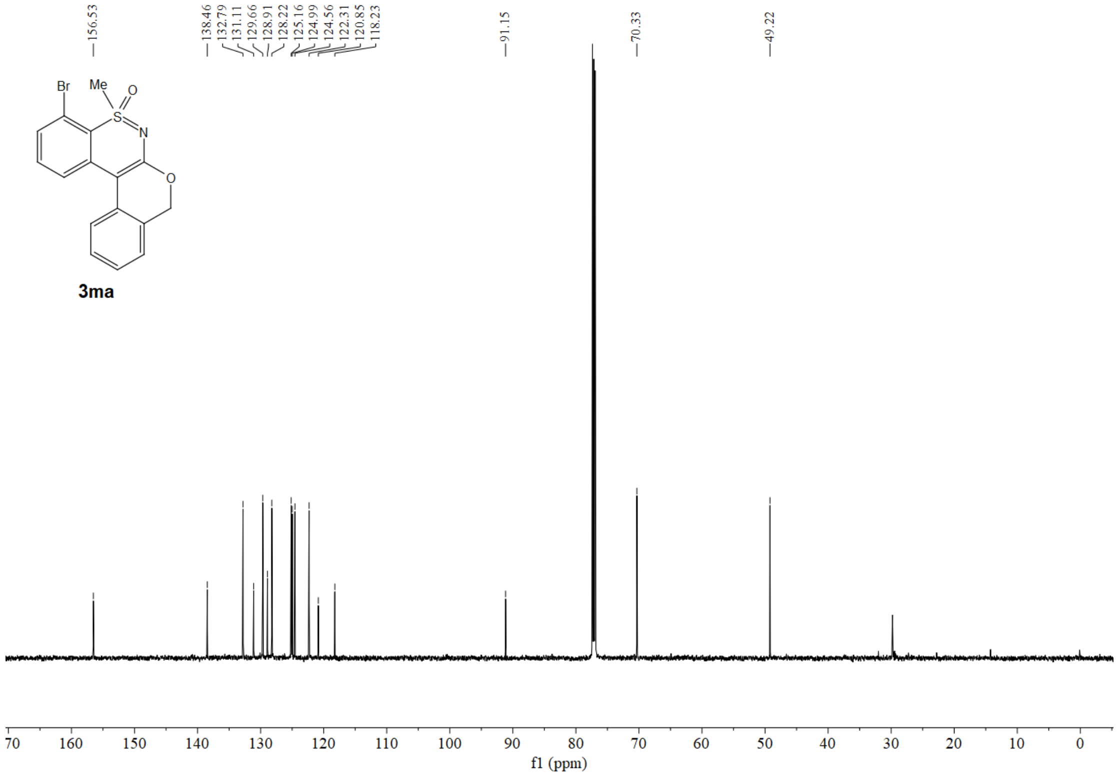

^{13}C NMR spectrum of **3ma**

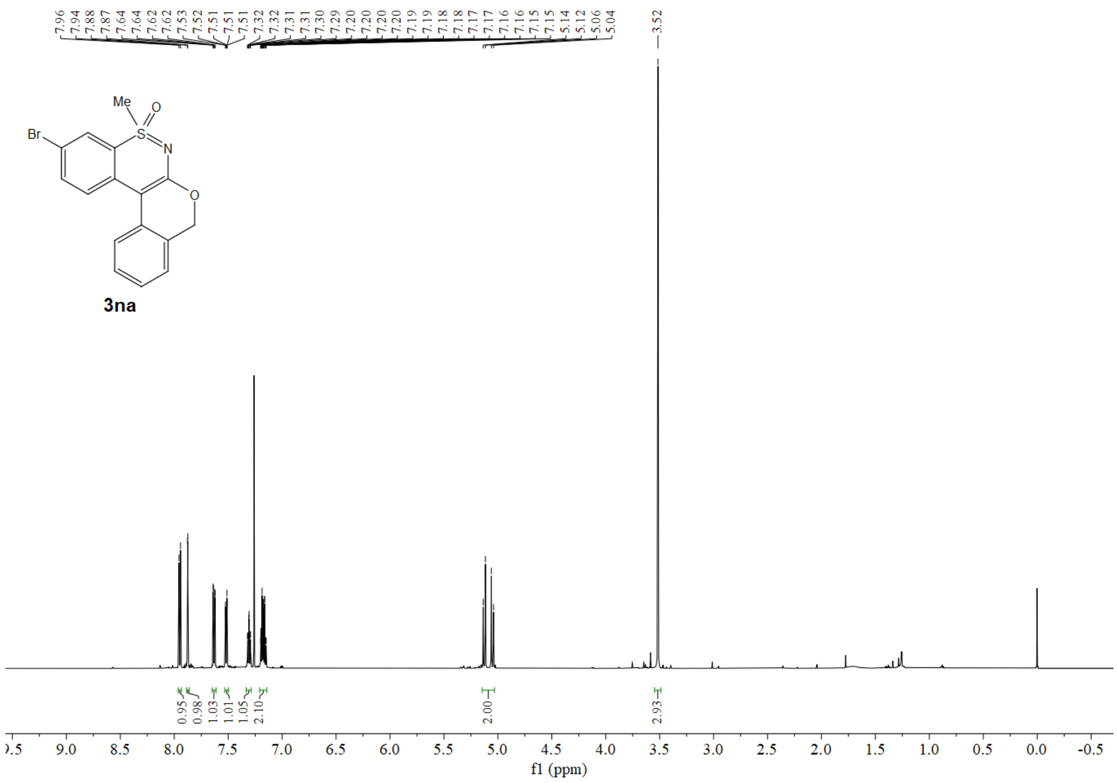

^{1}H NMR spectrum of **3na**

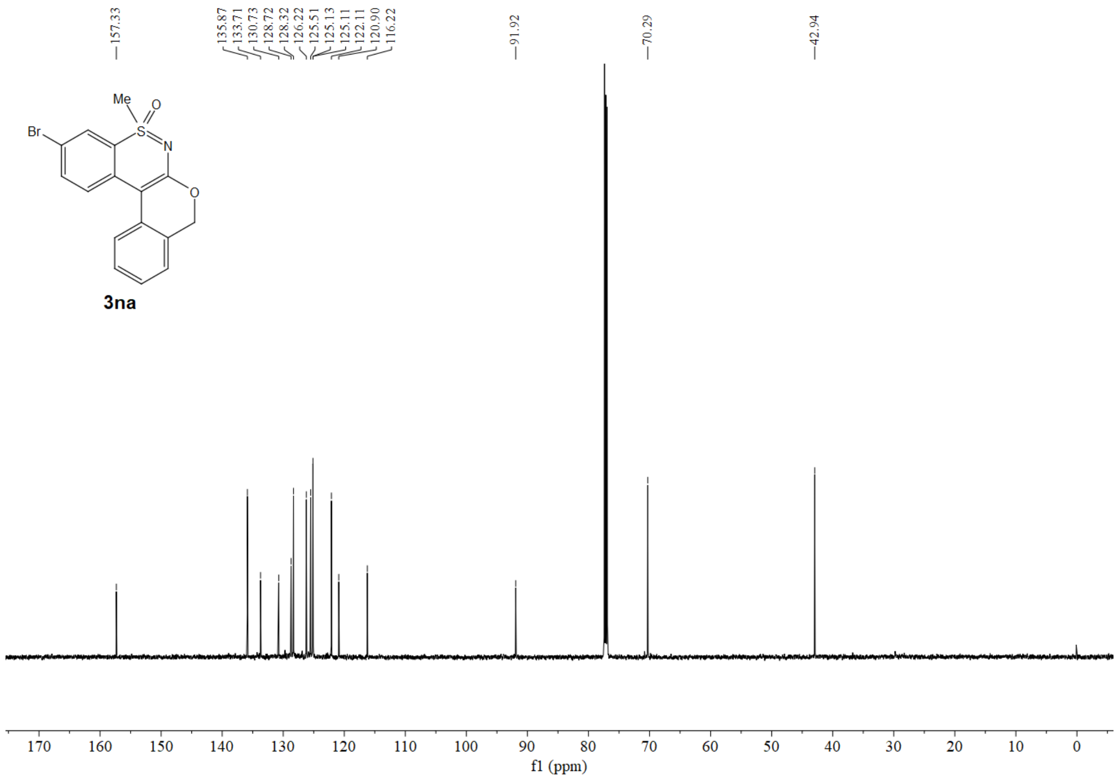

^{13}C NMR spectrum of **3na**

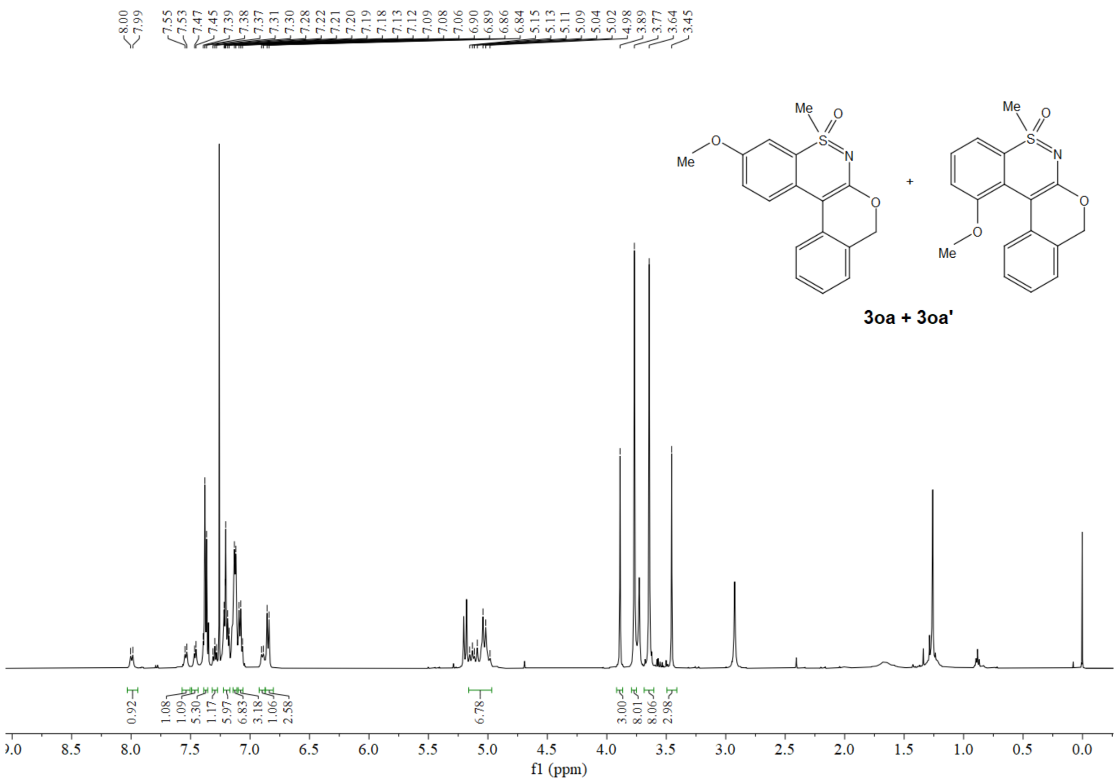

^{1}H NMR spectrum of a mixture of **3oa** and **3oa’**

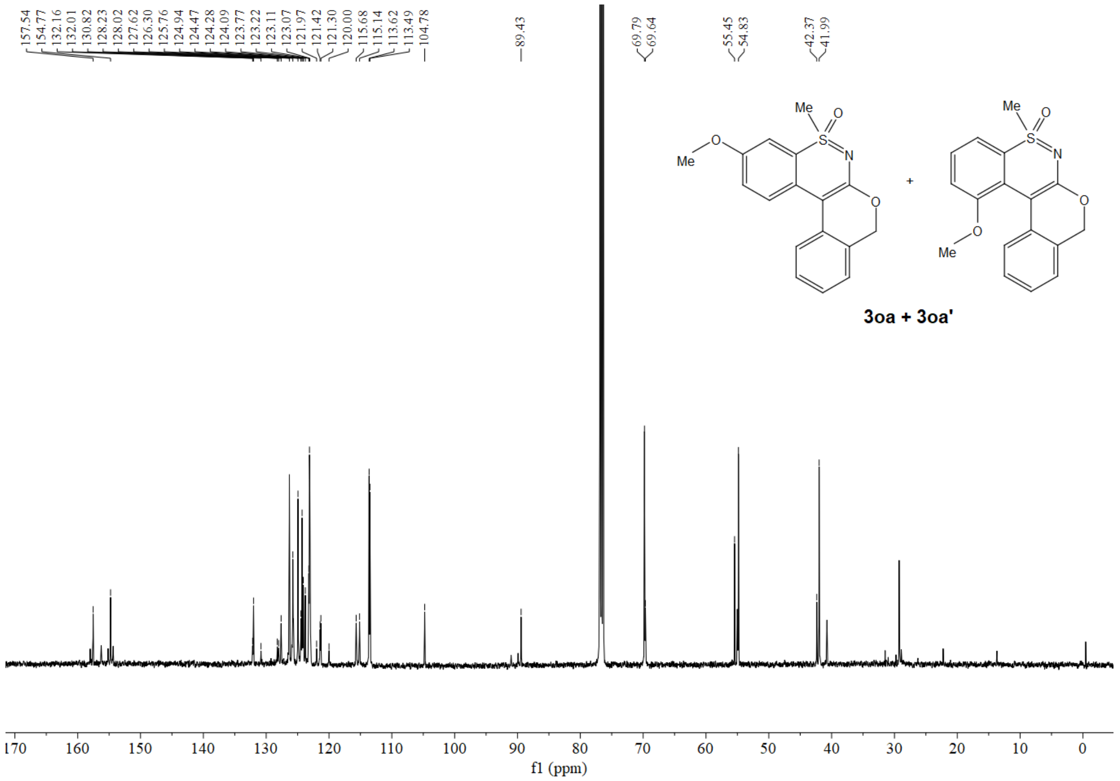

^{13}C NMR spectrum of a mixture of **3oa** and **3oa’**

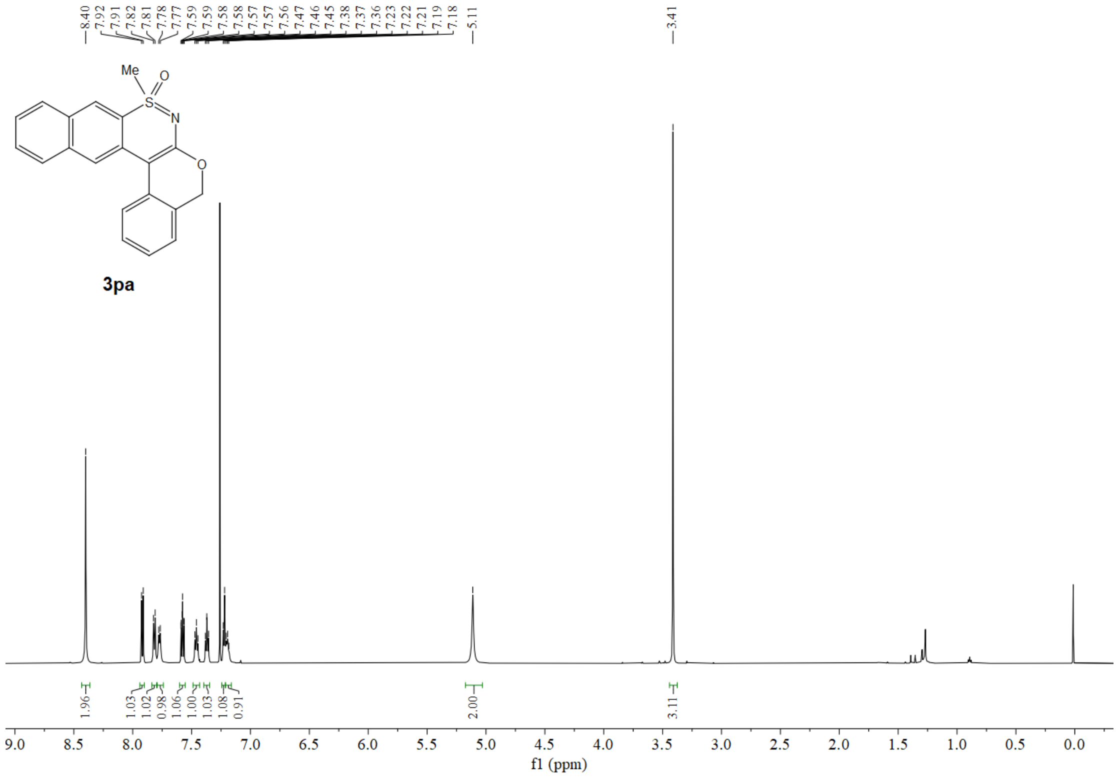

^{1}H NMR spectrum of **3pa**

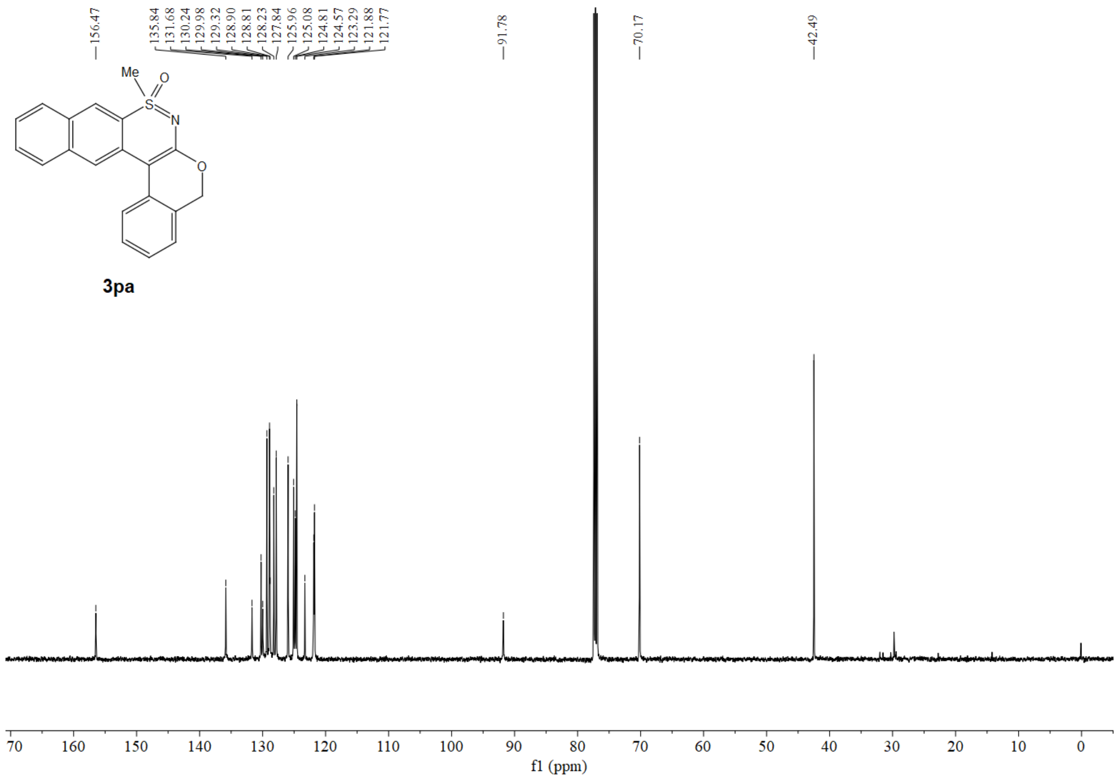

^{13}C NMR spectrum of **3pa**

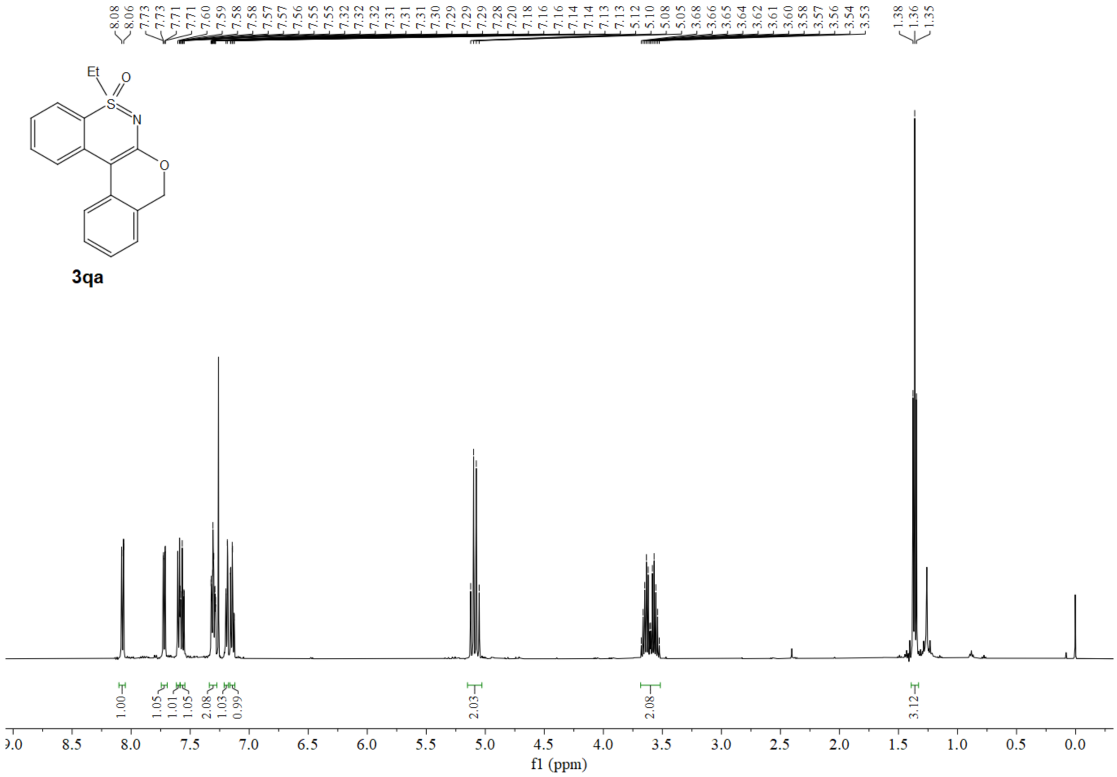

^{1}H NMR spectrum of **3qa**

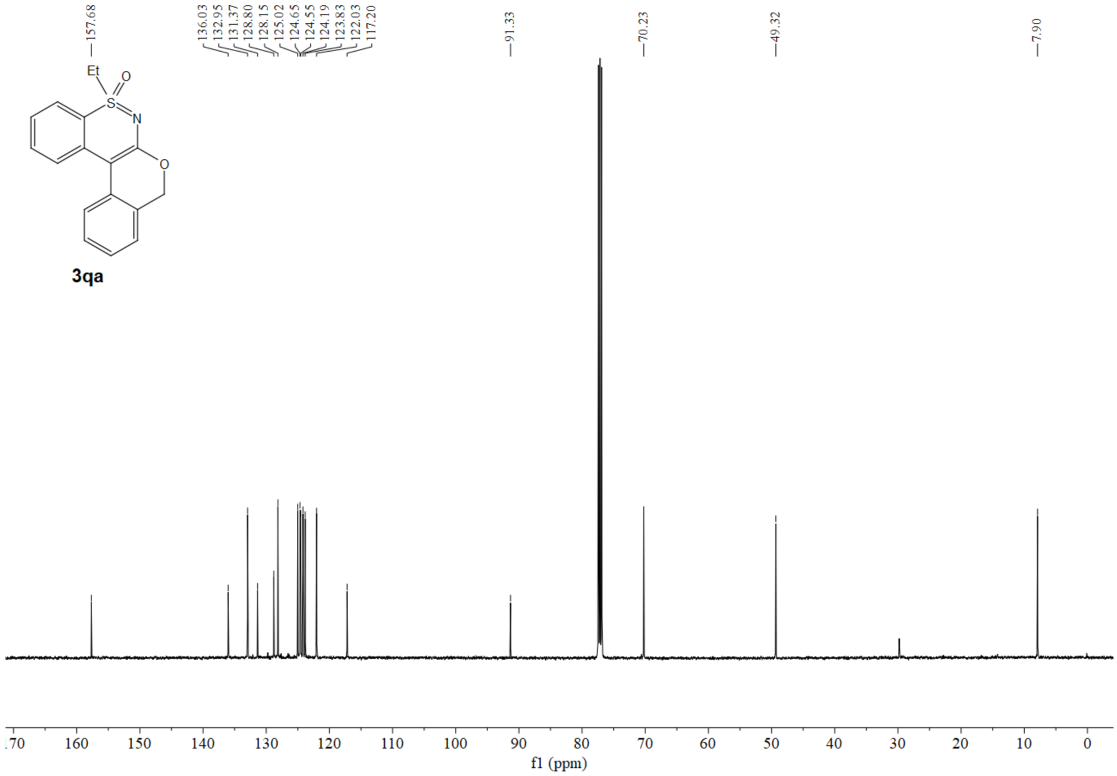

^{13}C NMR spectrum of **3qa**

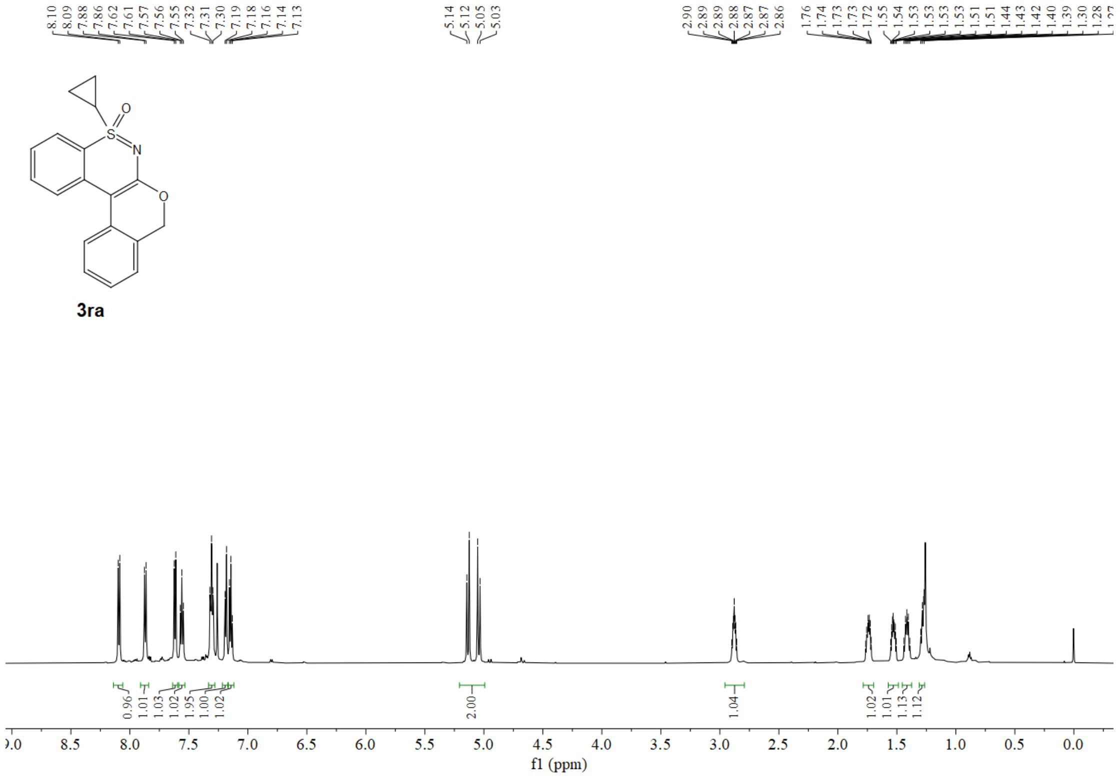

^{1}H NMR spectrum of **3ra**

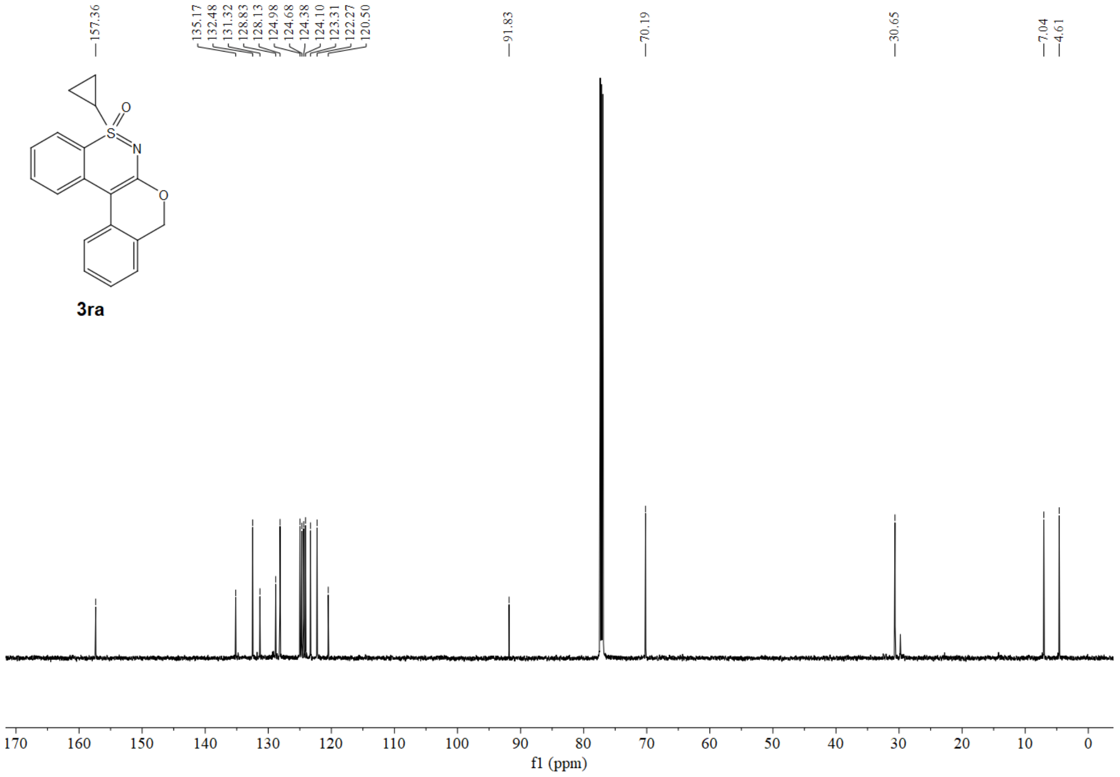

^{13}C NMR spectrum of **3ra**

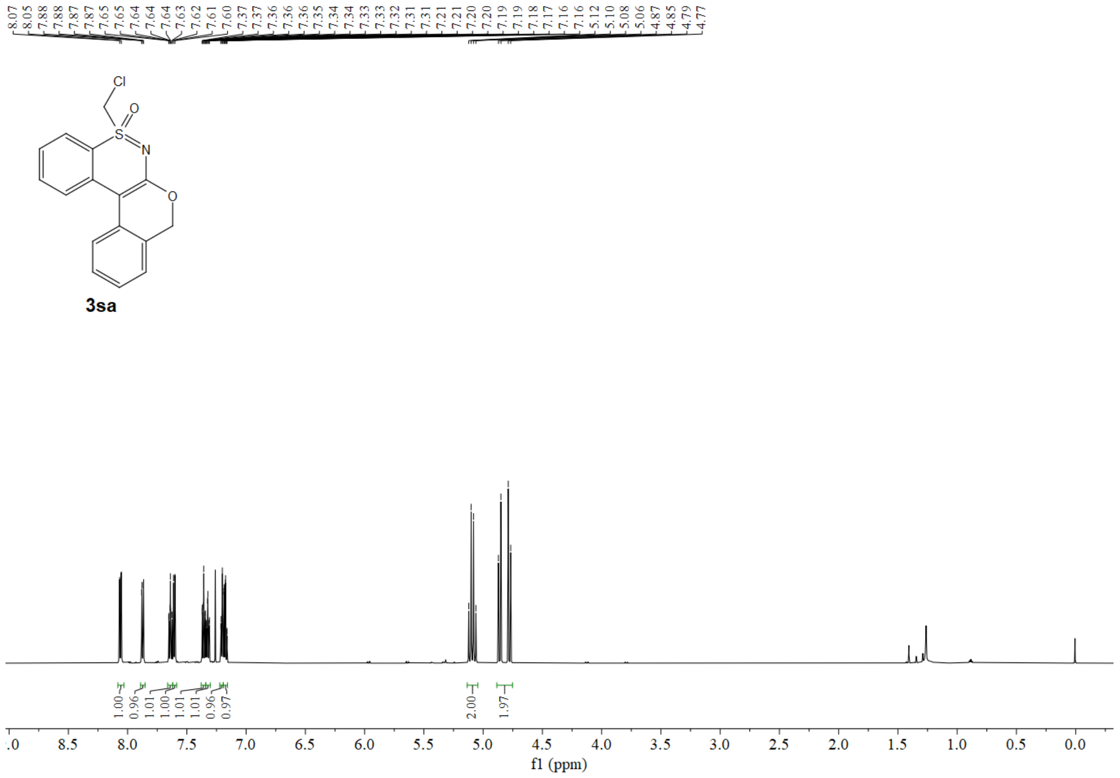

^{1}H NMR spectrum of **3sa**

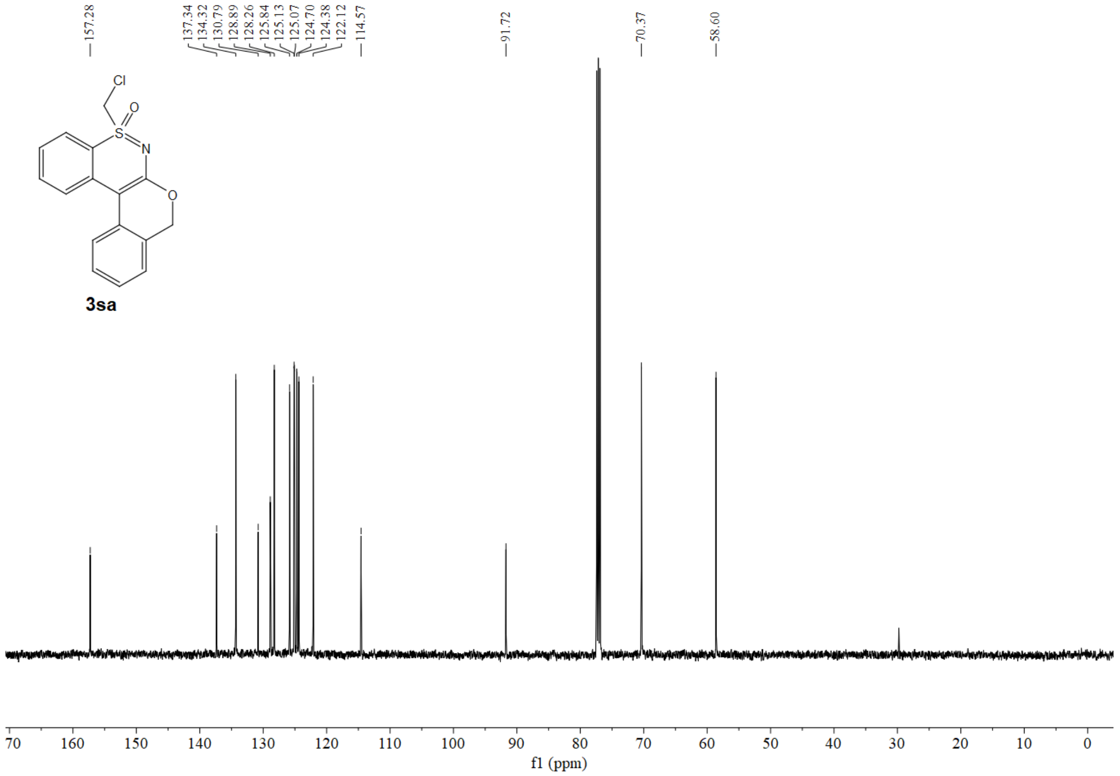

^{13}C NMR spectrum of **3sa**

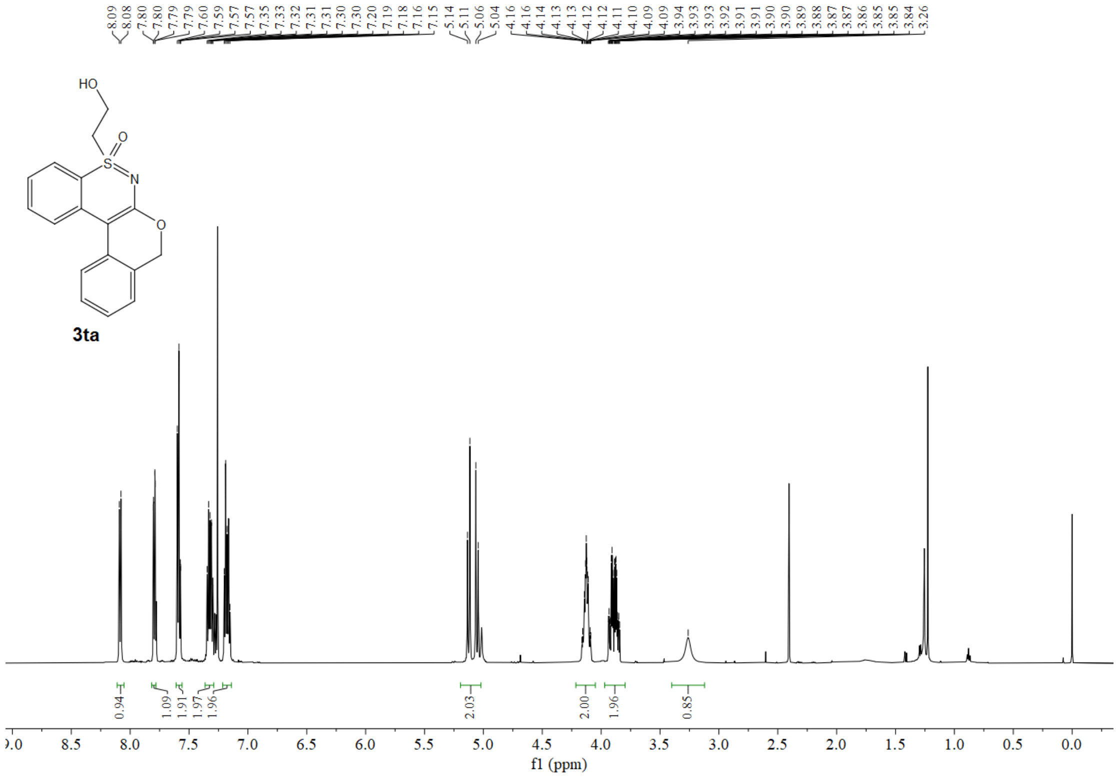

^{1}H NMR spectrum of **3ta**

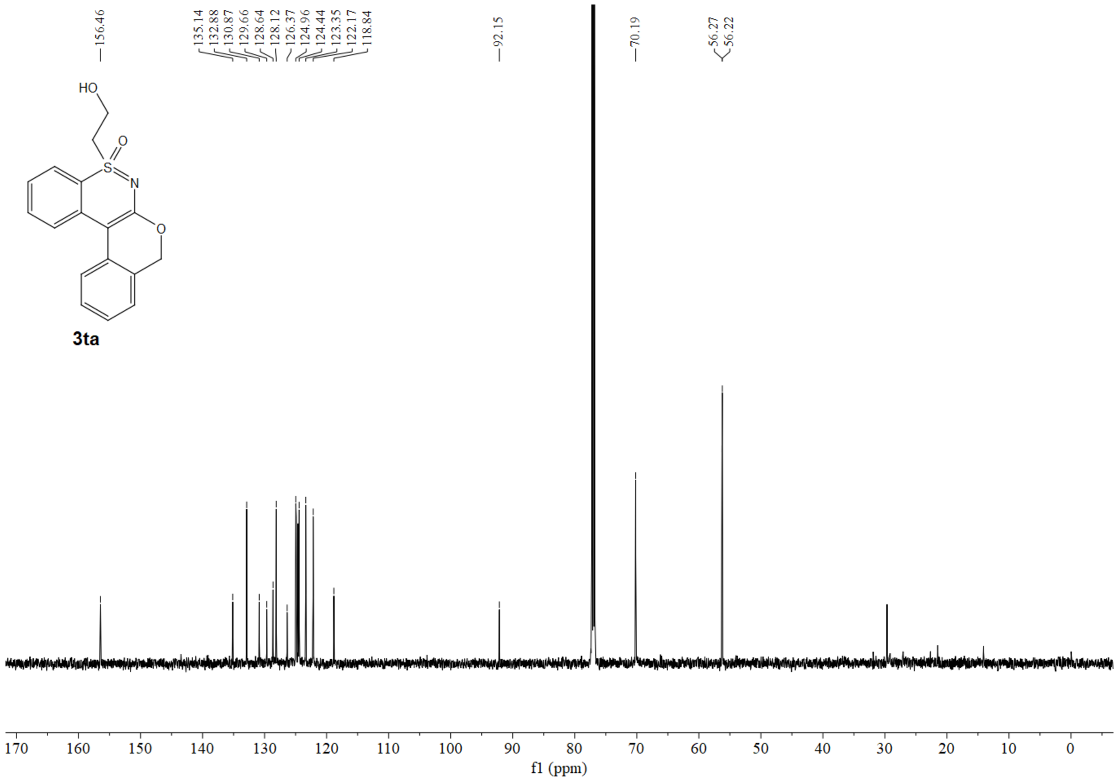

^{13}C NMR spectrum of **3ta**

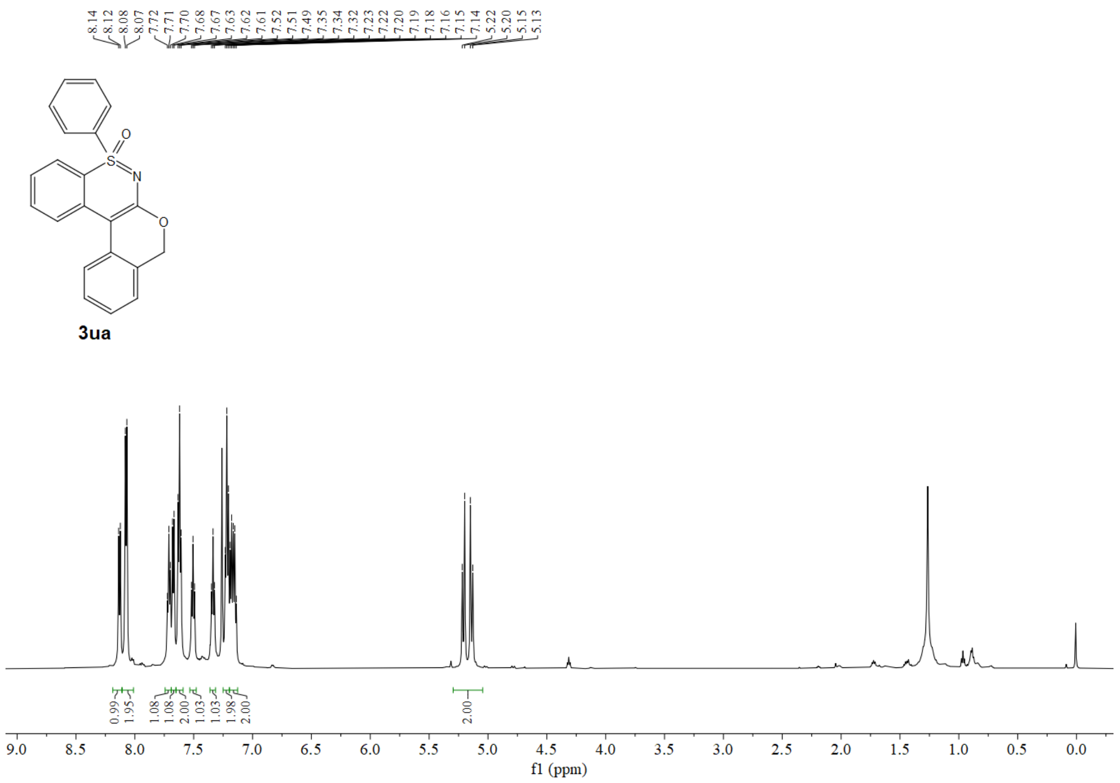

^{1}H NMR spectrum of **3ua**

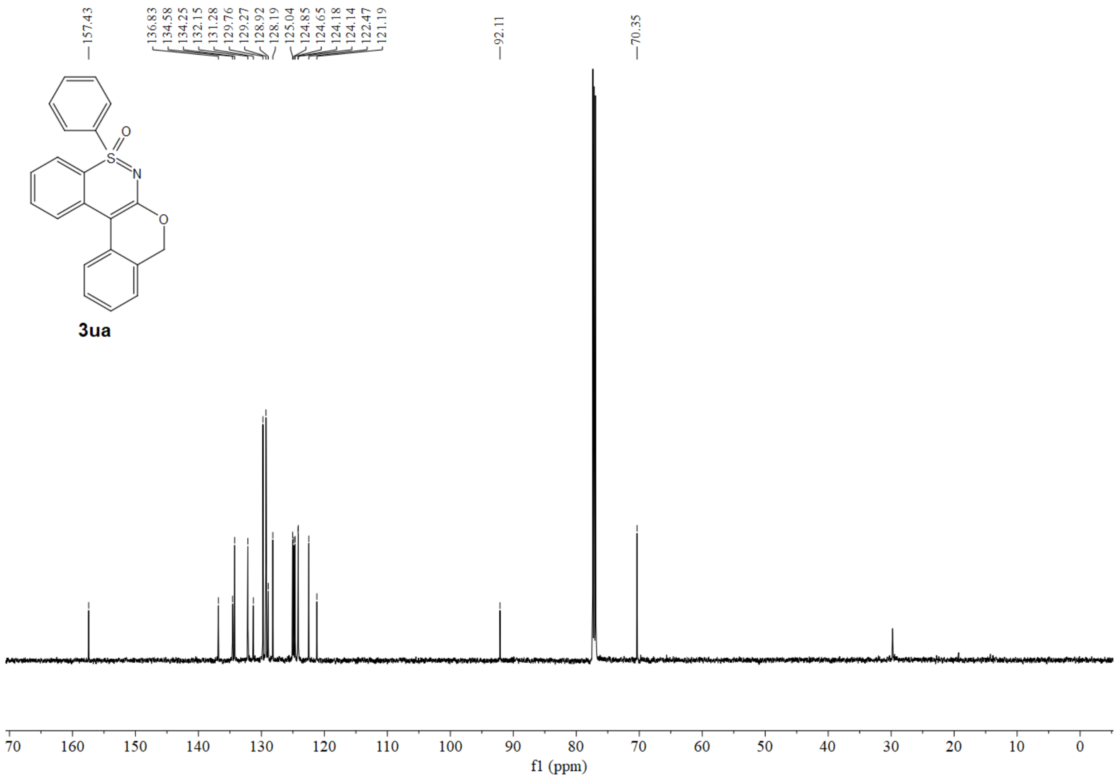

^{13}C NMR spectrum of **3ua**

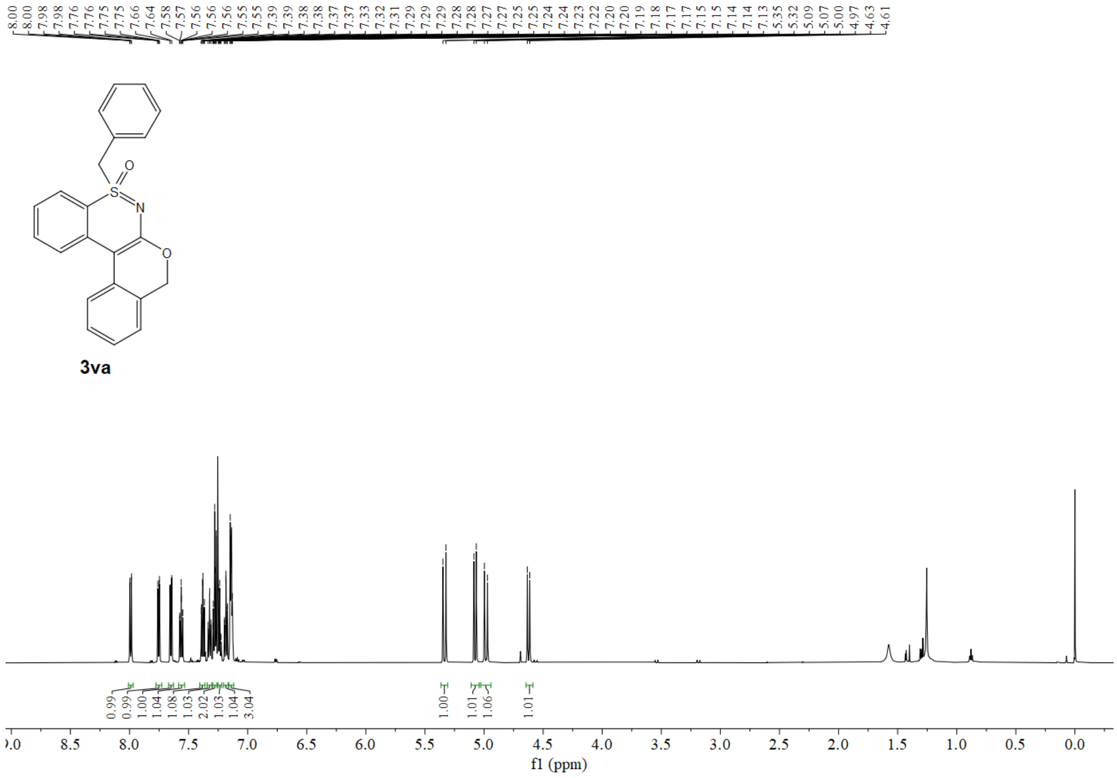

^{1}H NMR spectrum of **3va**

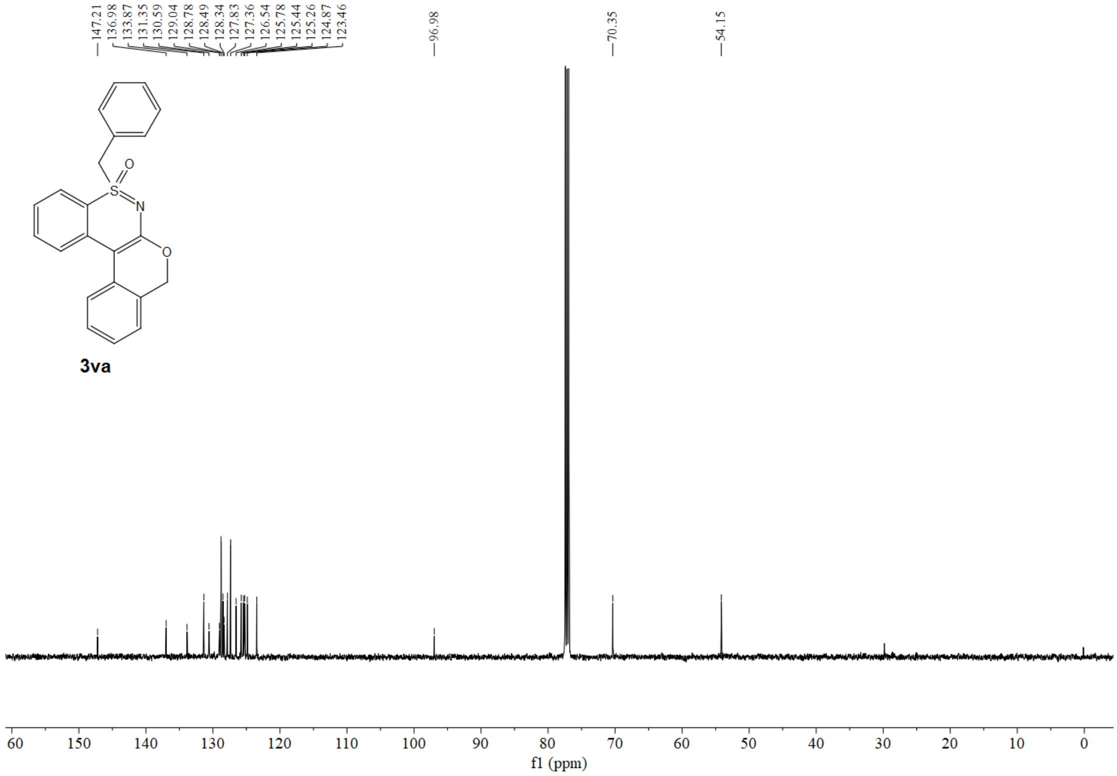

^{13}C NMR spectrum of **3va**

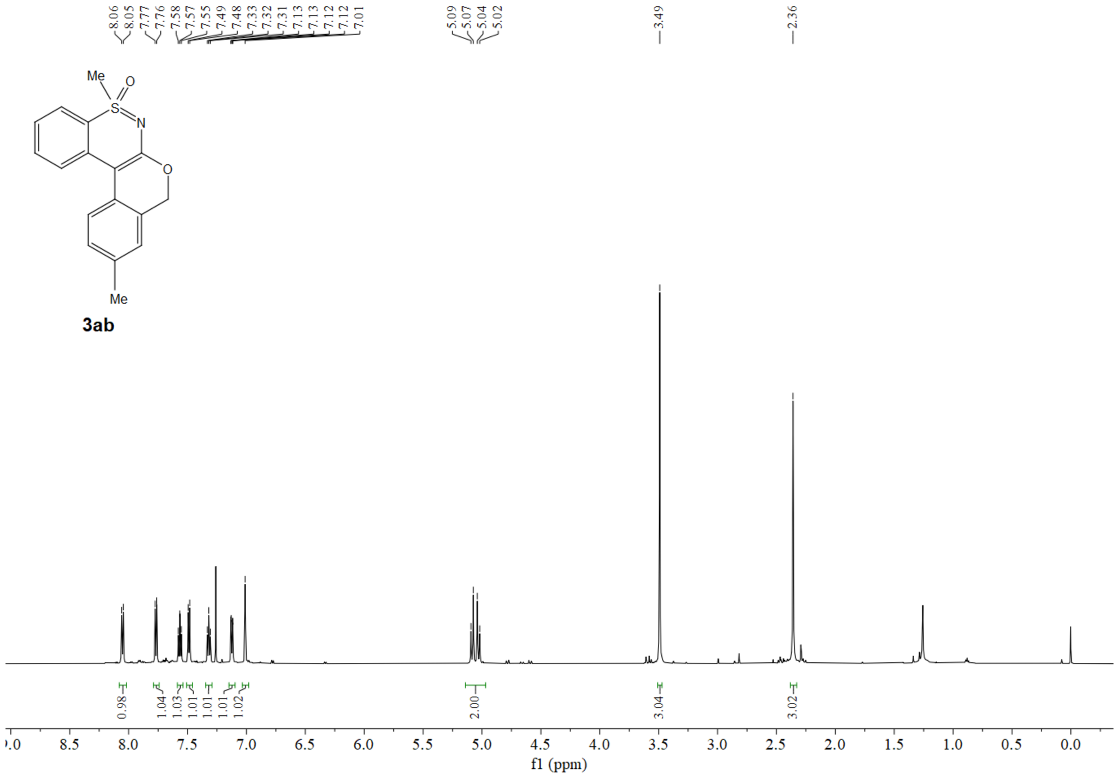

^{1}H NMR spectrum of **3ab**

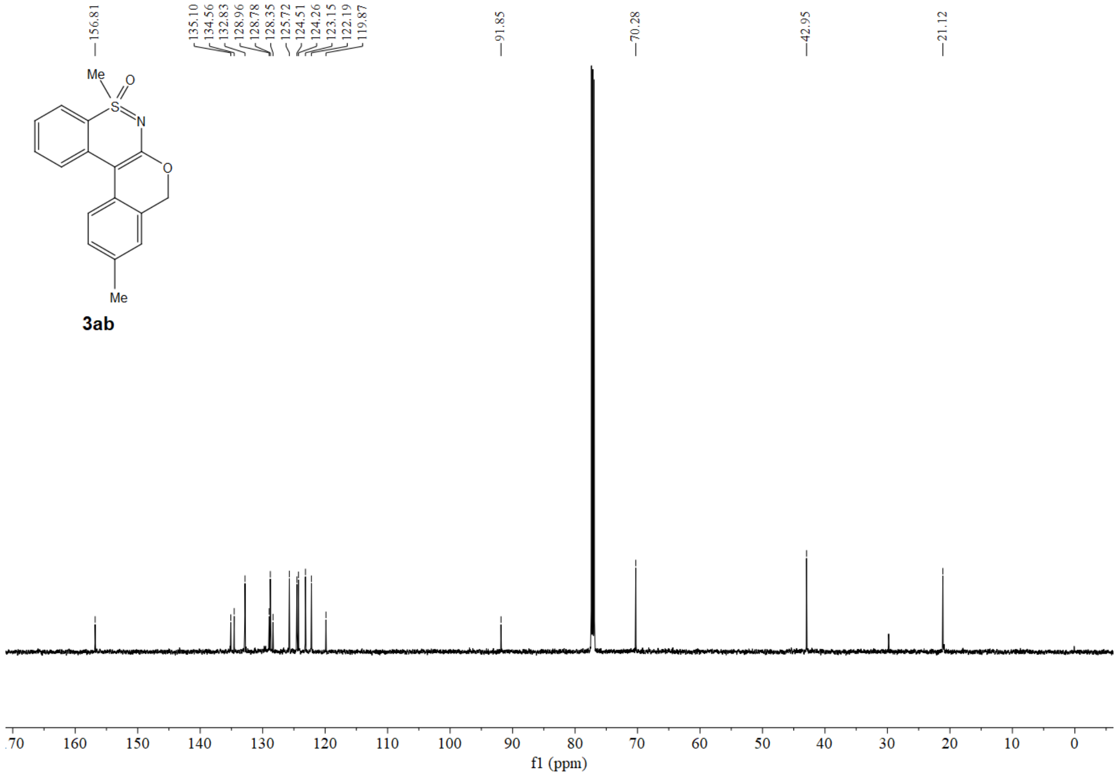

^{13}C NMR spectrum of **3ab**

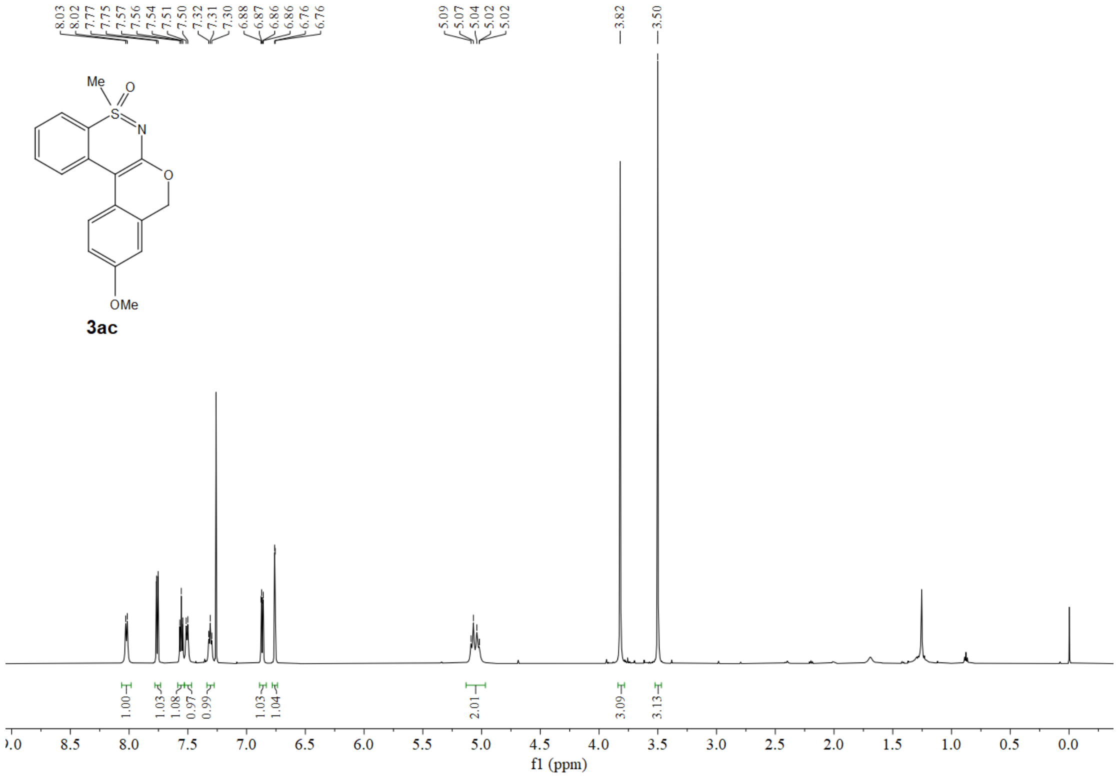

^{1}H NMR spectrum of **3ac**

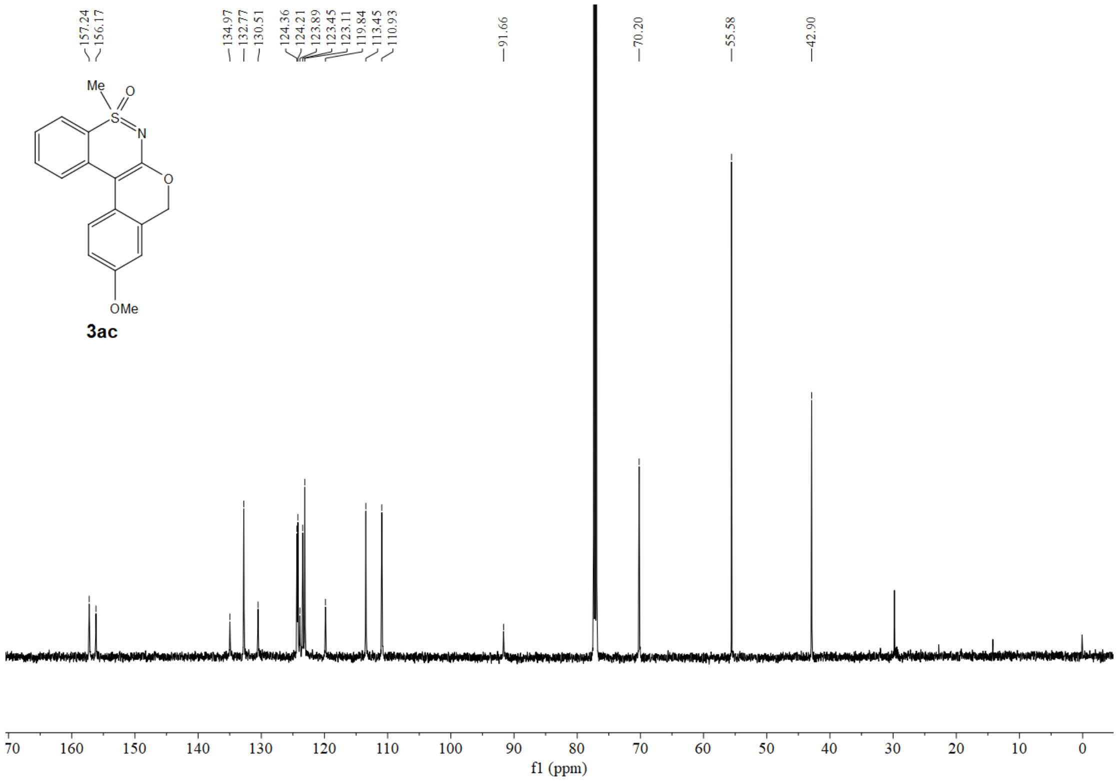

^{13}C NMR spectrum of **3ac**

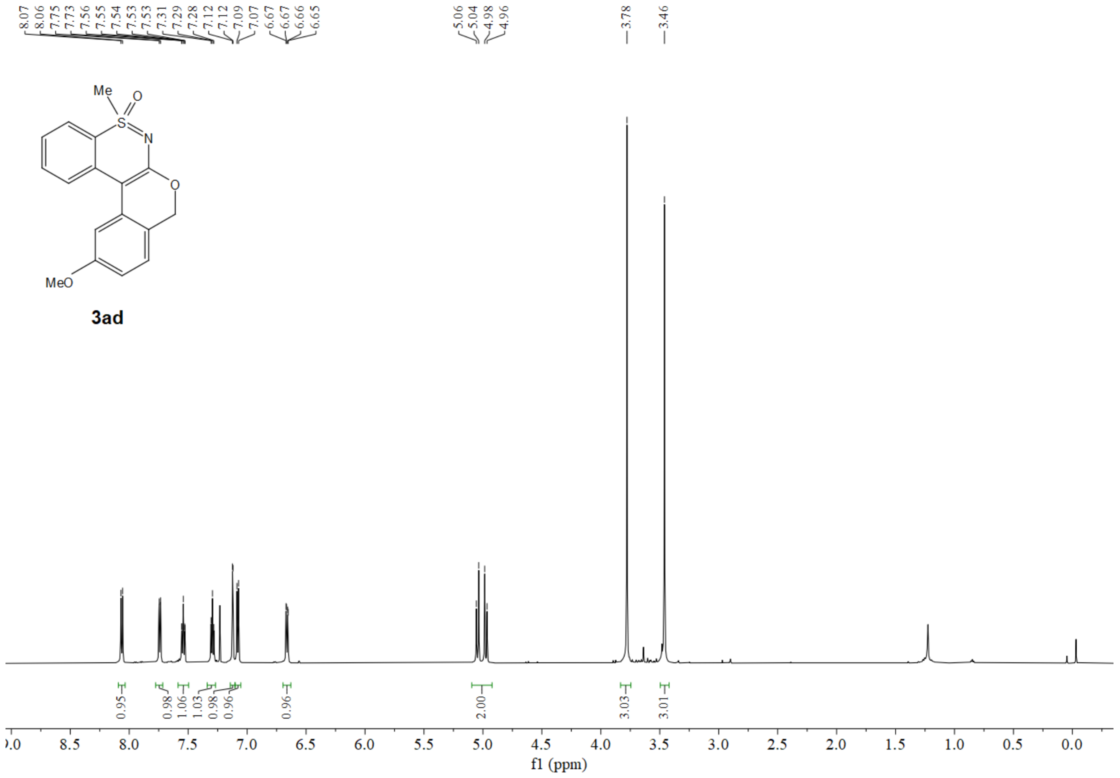

^{1}H NMR spectrum of **3ad**

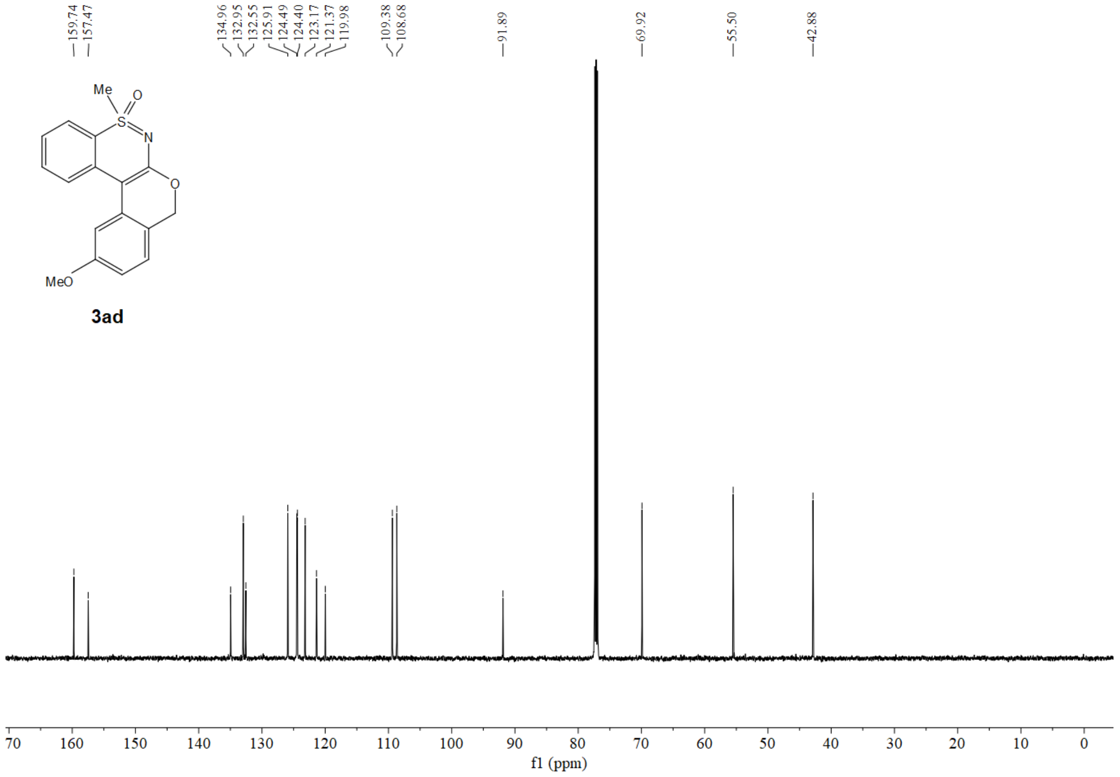

^{13}C NMR spectrum of **3ad**

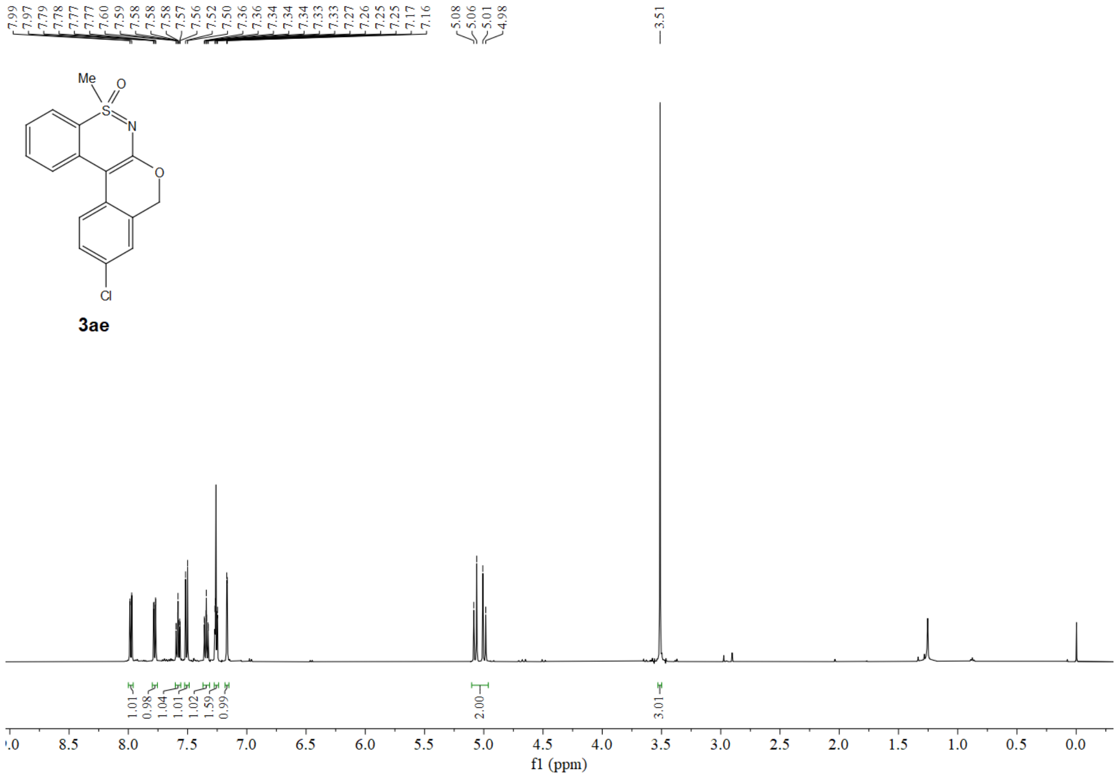

^{1}H NMR spectrum of **3ae**

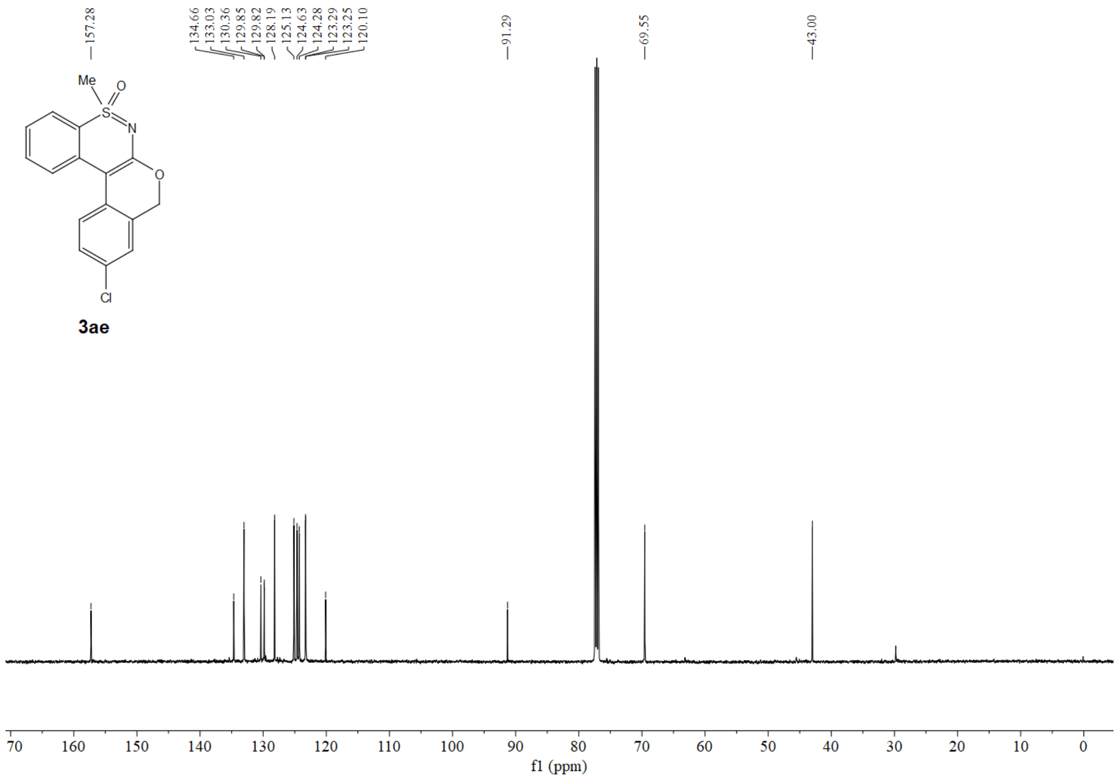

^{13}C NMR spectrum of **3ae**

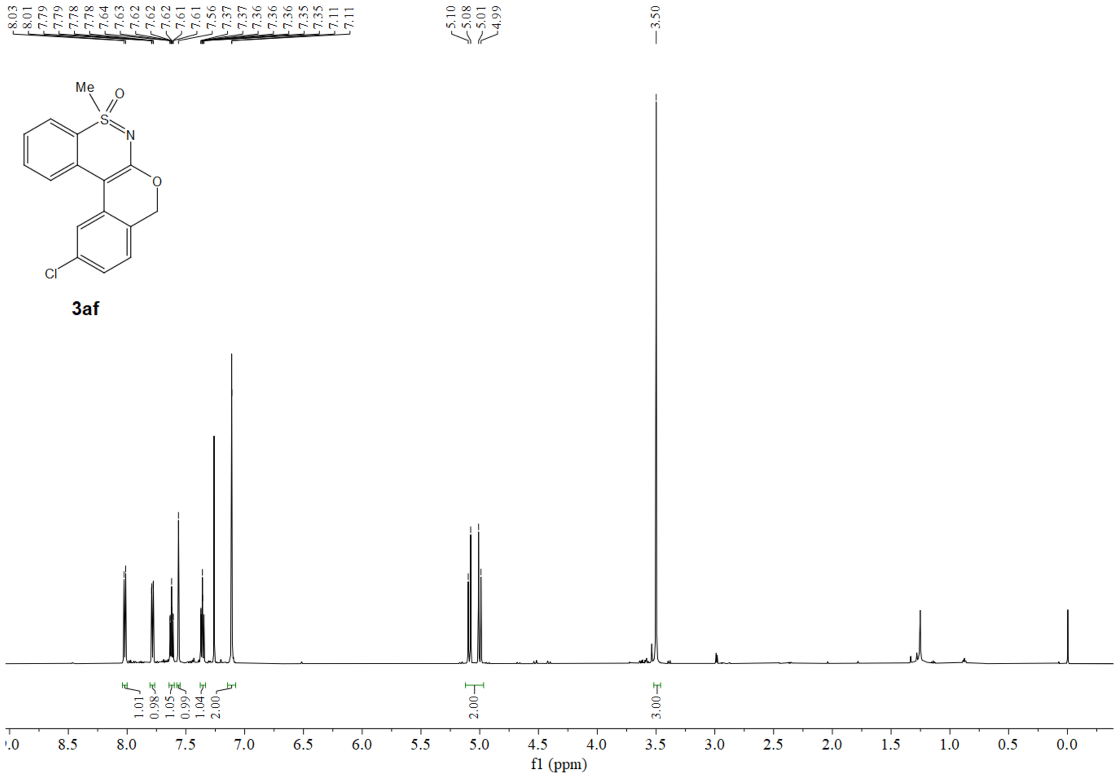

^{1}H NMR spectrum of **3af**

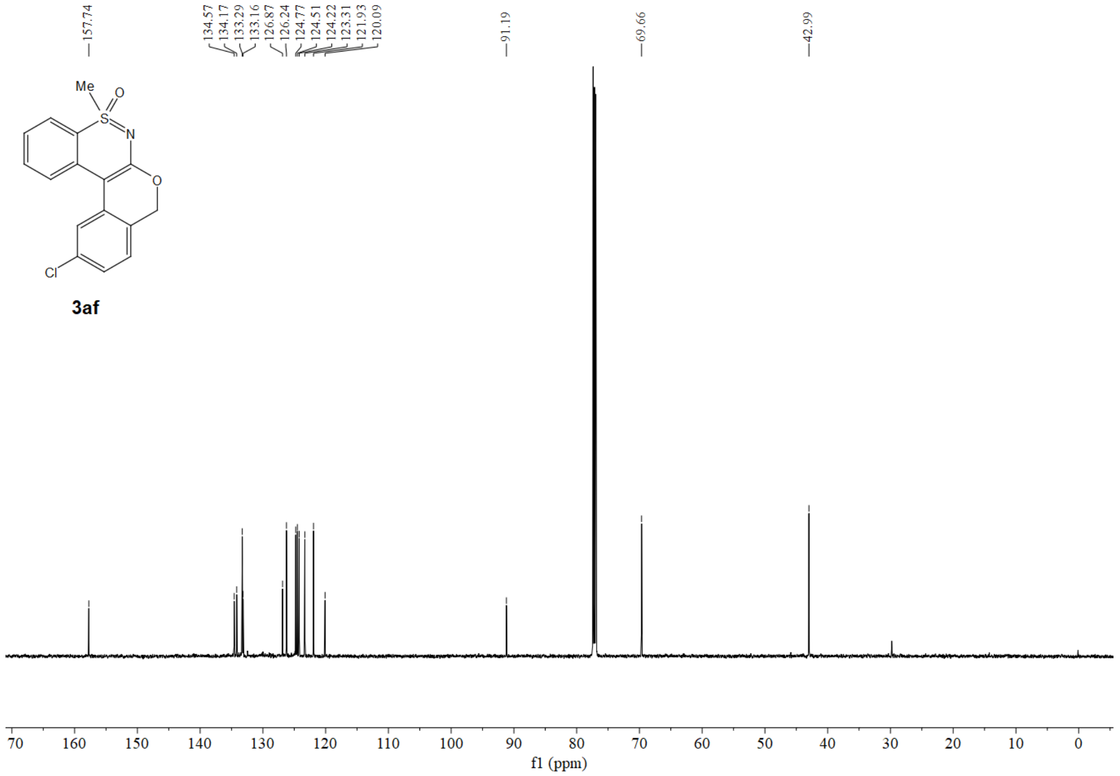

^{13}C NMR spectrum of **3af**

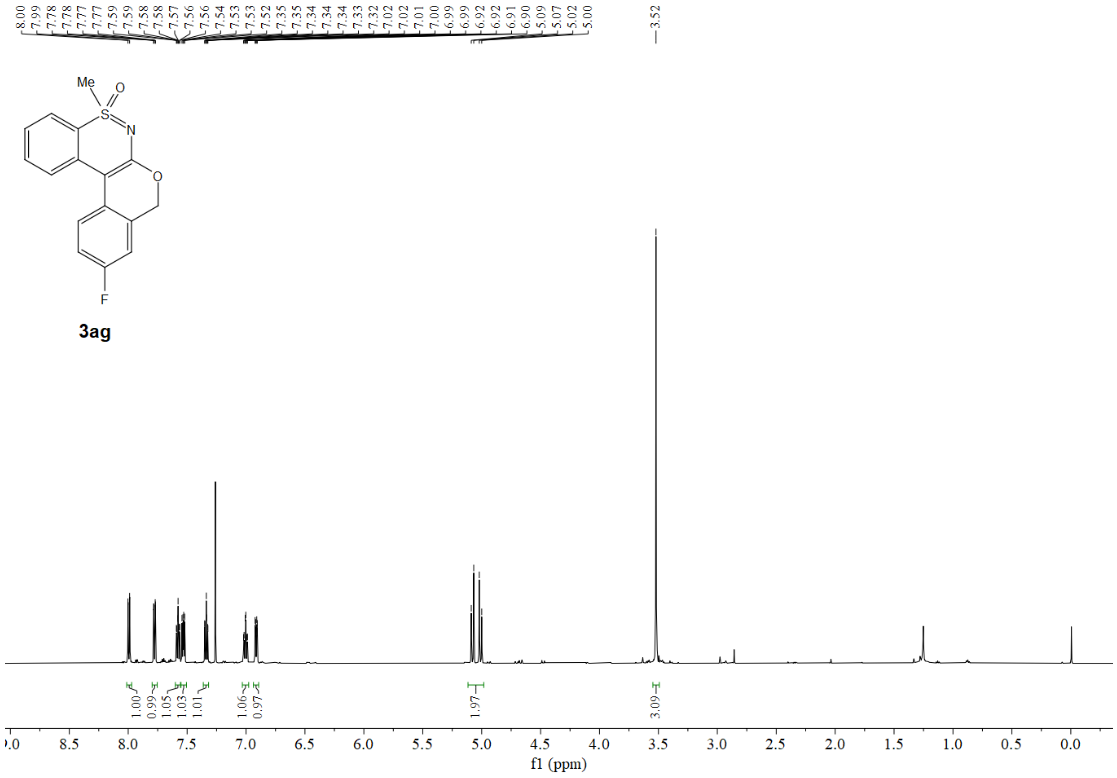

^{1}H NMR spectrum of **3ag**

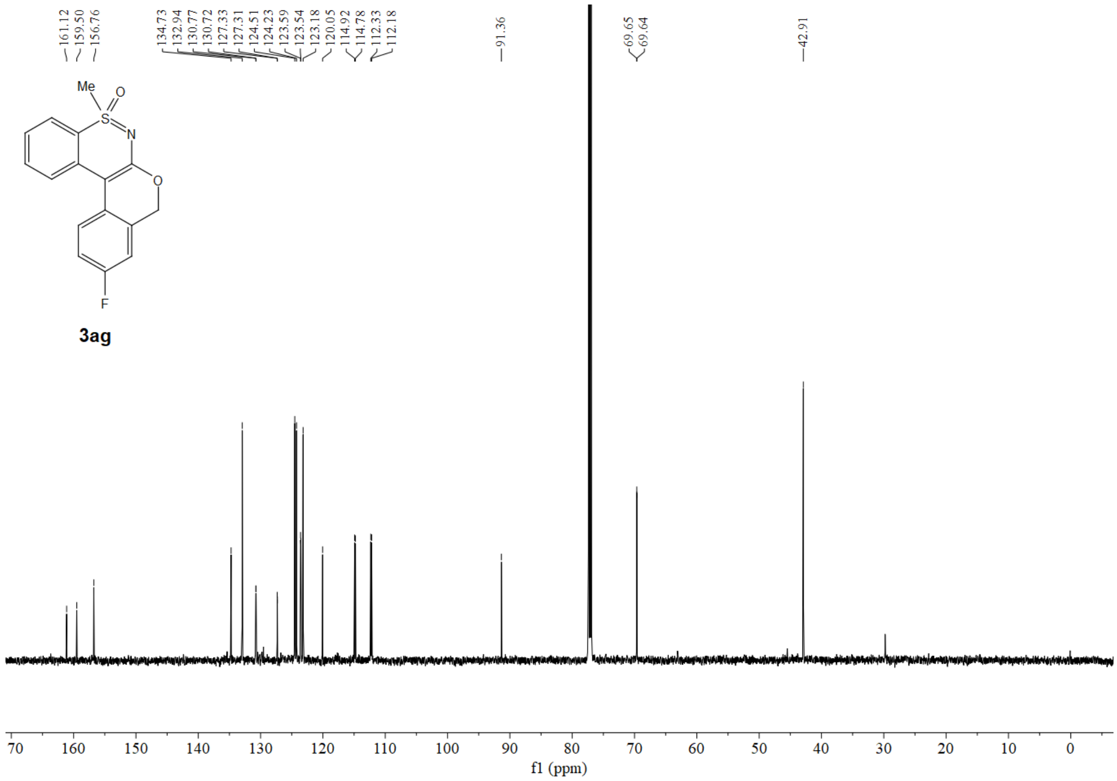

^{13}C NMR spectrum of **3ag**

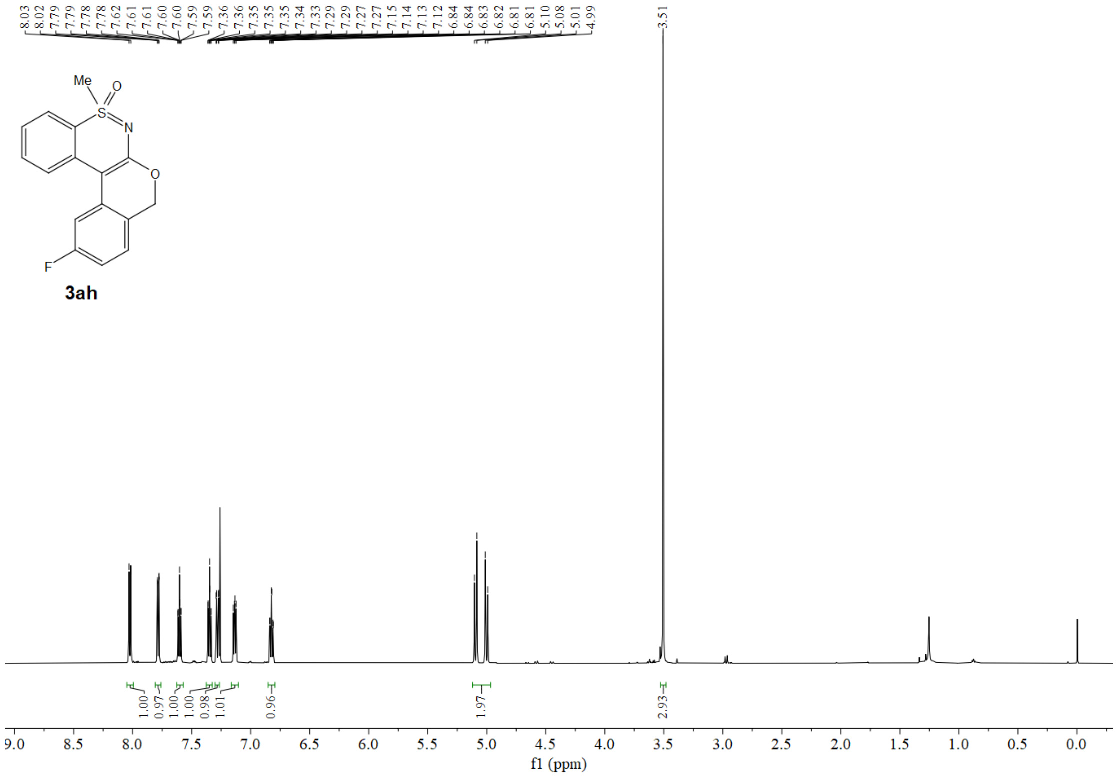

^{1}H NMR spectrum of **3ah**

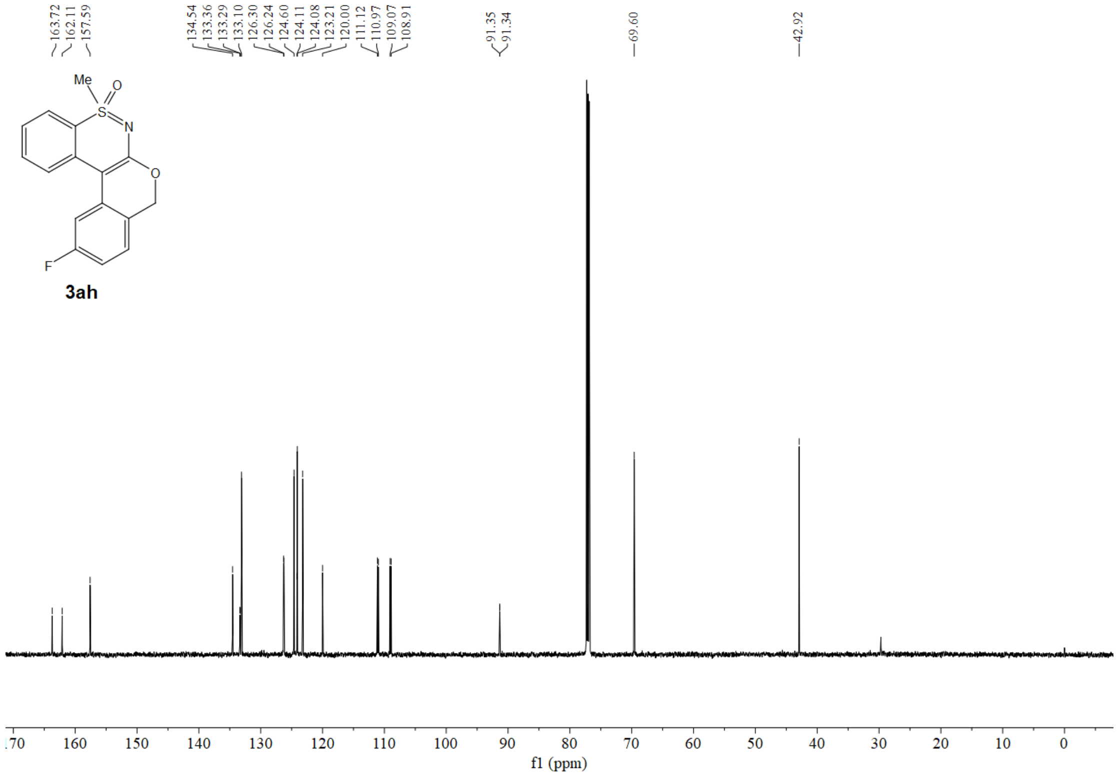

^{13}C NMR spectrum of **3ah**

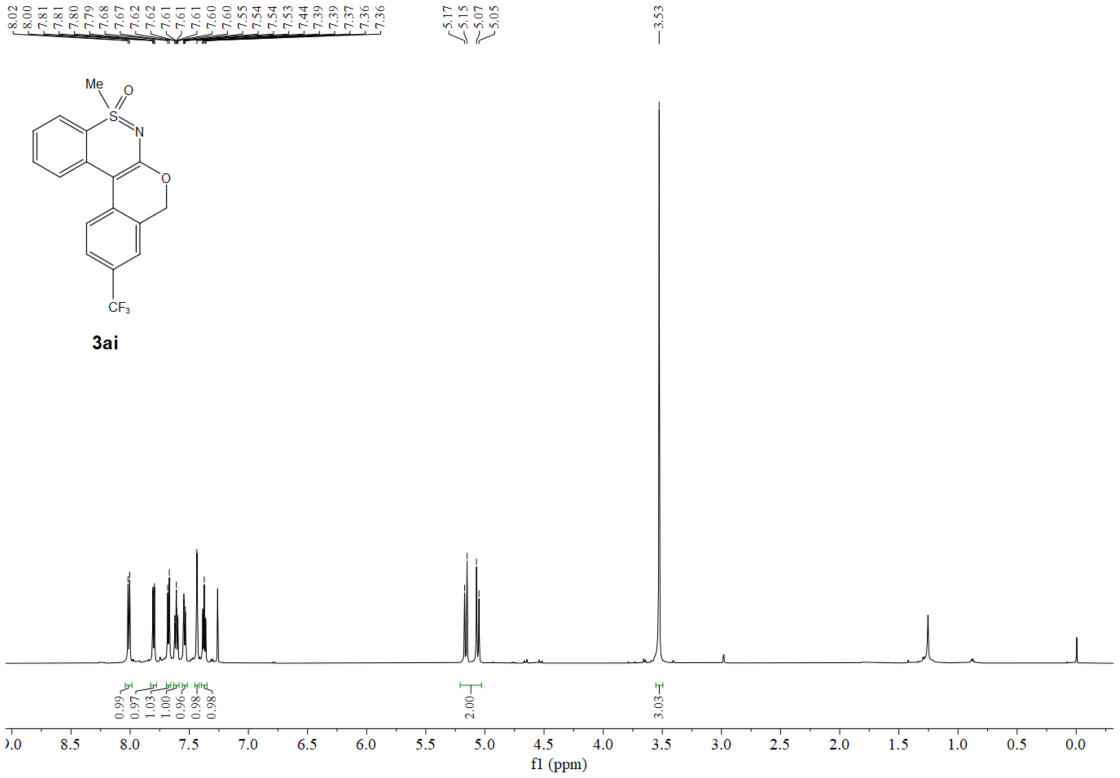

^{1}H NMR spectrum of **3ai**

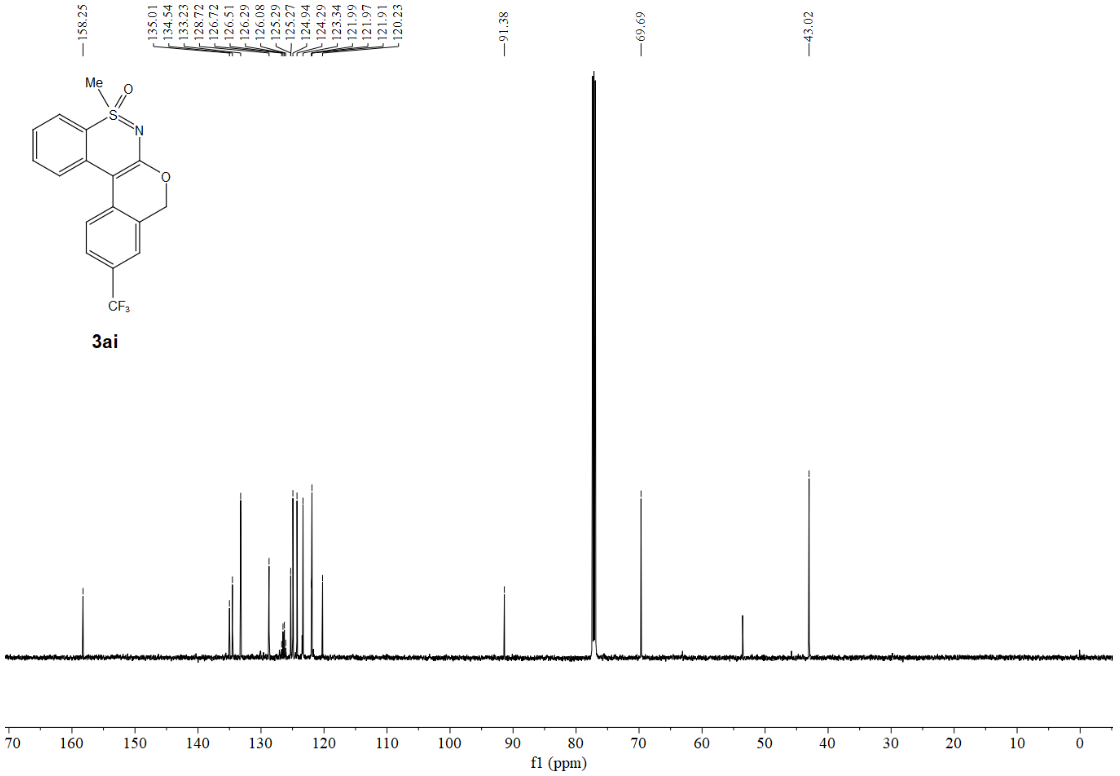

^{13}C NMR spectrum of **3ai**

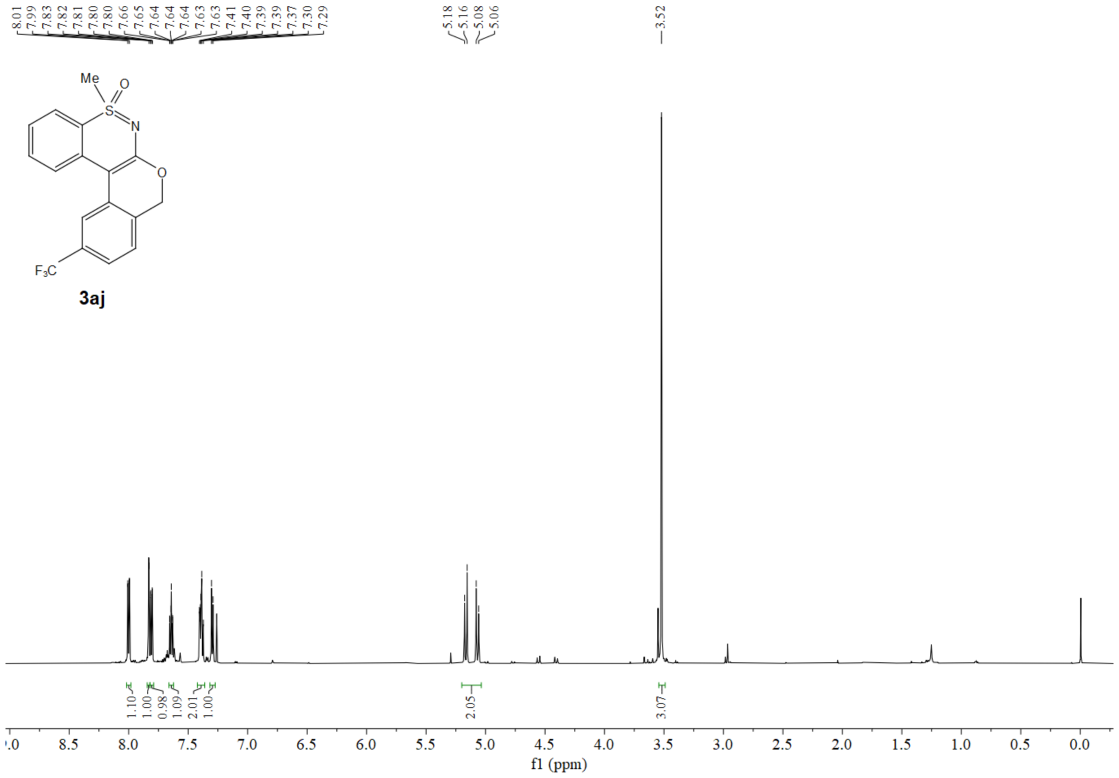

^{1}H NMR spectrum of **3aj**

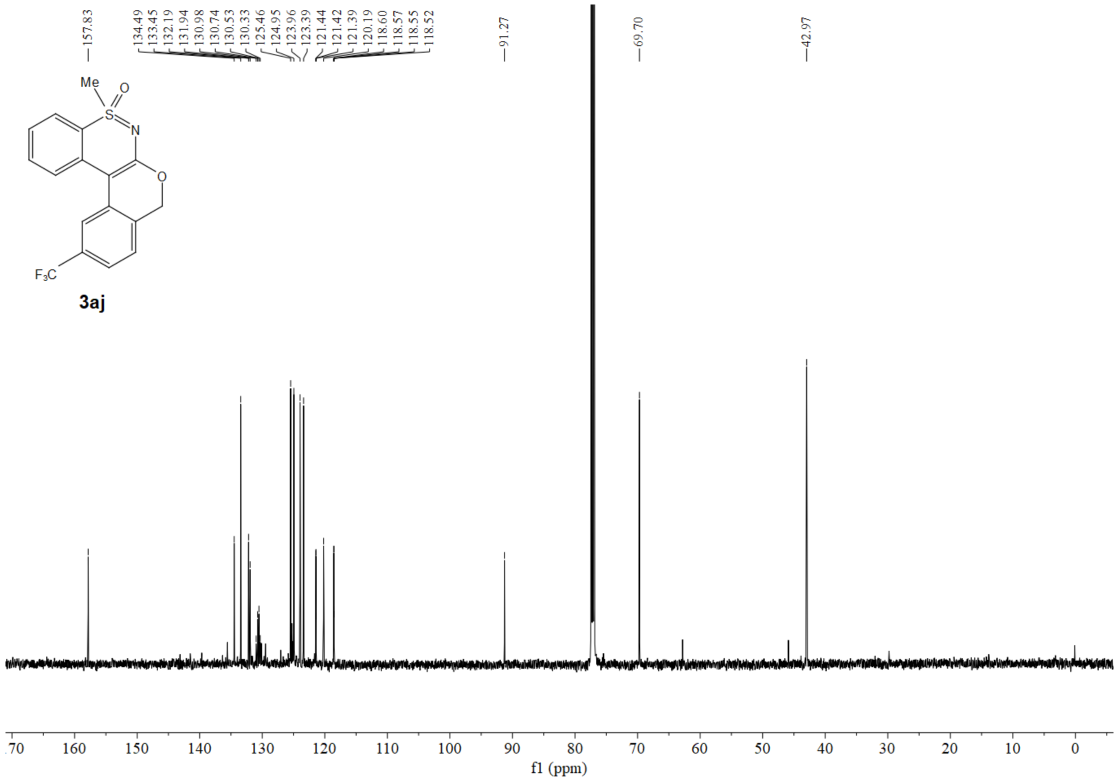

^{13}C NMR spectrum of **3aj**
